# Nickel-catalyzed cyanation of aryl halides and triflates using acetonitrile *via* C–CN bond cleavage assisted by 1,4-bis(trimethylsilyl)-2,3,5,6-tetramethyl-1,4-dihydropyrazine†
†Electronic supplementary information (ESI) available: Experimental details for the synthesis of *Si*–Me_4_-DHP and **6**, screening of reaction conditions, identification of the products, and crystal data for **6**. CCDC 1852145. For ESI and crystallographic data in CIF or other electronic format see DOI: 10.1039/c8sc04437f


**DOI:** 10.1039/c8sc04437f

**Published:** 2018-11-26

**Authors:** Yohei Ueda, Nagataka Tsujimoto, Taiga Yurino, Hayato Tsurugi, Kazushi Mashima

**Affiliations:** a Department of Chemistry , Graduate School of Engineering Science , Osaka University , Toyonaka , Osaka 560-8531 , Japan . Email: tsurugi@chem.es.osaka-u.ac.jp ; Email: mashima@chem.es.osaka-u.ac.jp

## Abstract

We developed a non-toxic cyanation reaction of various aryl halides and triflates in acetonitrile using a catalyst system of [Ni(MeCN)_6_](BF_4_)_2_, 1,10-phenanthroline, and 1,4-bis(trimethylsilyl)-2,3,5,6-tetramethyl-1,4-dihydropyrazine (*Si*–Me_4_-DHP). *Si*–Me_4_-DHP was found to function as a reductant for generating nickel(0) species and a silylation reagent to achieve the catalytic cyanation *via* C–CN bond cleavage.

## Abstract 2

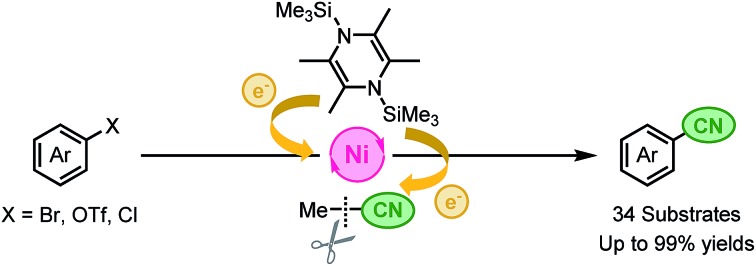
A catalyst system of [Ni(MeCN)_6_](BF_4_)_2_, 1,10-phenanthroline, and 1,4-bis(trimethylsilyl)-2,3,5,6-tetramethyl-1,4-dihydropyrazine (Si–Me_4_-DHP) assisted cyanation of aryl halides in acetonitrile to give the corresponding aryl nitriles.

## 

Nitriles are highly versatile functional compounds often used to prepare the corresponding amines, amides, and carboxylic acids.^1^ Several useful synthetic protocols have been established as not only stoichiometric reactions, such as the Sandmayer reaction and Rosenmund-von Braun reaction using CuCN,^2^ but also metal-catalyzed cyanation reactions of aryl halides or aryl triflates using toxic cyanide reagents,1f,^3^ such as NaCN,^4^ KCN,^5^ CuCN,^6^ Zn(CN)_2_,^7^ and Me_3_SiCN.^8^ Recent efforts have been aimed at developing non-toxic cyanation reactions using alkylnitriles,5h,^9^ despite the relatively high bond dissociation energy of the C–CN bond (*e.g.* 133 kcal mol^–1^ for the C–CN bond of acetonitrile) compared with that of the typical C(sp^3^)–C(sp^3^) bond (*ca.* 83 kcal mol^–1^).^10^ In fact, pivotal studies using acetonitrile as a cyano source were recently achieved at high temperature (120–160 °C): Cheng *et al.* used phosphine complexes of palladium and nickel for the catalytic cyanation of *ortho*-mono and *ortho*-disubstituted aryl halides with acetonitrile in the presence of zinc powder at elevated temperature (160 °C) (Fig. 1a),9*d* and Shen *et al.* applied a catalyst system of copper(ii) nitrate with 2,2,6,6-tetramethylpiperidine-1-oxyl (TEMPO) for the cyanation of aryl iodides in acetonitrile at high temperature (150 °C) (Fig. 1b).9e,f Quite recently, Morandi *et al.* demonstrated that a combination of bis(acetylacetonato)nickel(ii) with Xantphos became a catalyst for the cyanation of aryl chlorides and aryl triflates with *n*-butyronitrile as a cyano source in the presence of highly reactive Lewis acids such as tri(isobutyl)aluminum at high temperature (120 °C) (Fig. 1c).9*g*

**Fig. 1:**
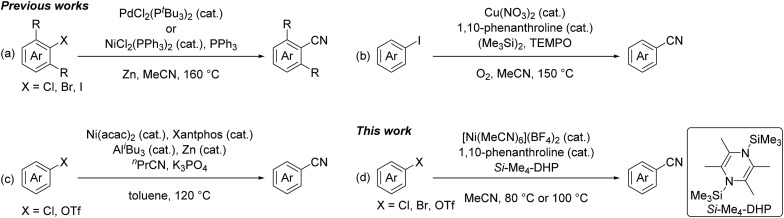
Recent catalytic cyanation reactions using alkylnitriles.

In this context, we have continuously focused our efforts on finding a catalyst system active for such cyanation using acetonitrile under milder reaction conditions. Herein, we report a nickel-catalyzed cyanation of aryl halides and aryl triflates with acetonitrile at 80 °C or 100 °C in the presence of an organosilicon compound, 1,4-bis(trimethylsilyl)-2,3,5,6-tetramethyl-1,4-dihydropyrazine (*Si*–Me_4_-DHP), which served as both an effective reductant and a silylating reagent for nickel species (Fig. 1d).^11^ It is noteworthy that C–CN bond cleavage of acetonitrile was achieved by α-methyl elimination from an [*N*-(trimethylsilyl)iminoacyl]nickel intermediate, which was generated by insertion of acetonitrile into Ni–SiMe_3_ species.

We started by searching for the best nickel catalyst system for the cyanation of 4-bromoanisole (**1a**) in acetonitrile, and the results are summarized in Table 1. The reaction mixture of **1a** and *Si*–Me_4_-DHP (2.5 equiv.) in the presence of catalytic amounts of [Ni(MeCN)_6_](BF_4_)_2_ (**2**, 5 mol%) and 2,2′-bipyridine (**L1**, 5 mol%) in acetonitrile was heated at 80 °C for 24 h to give *p*-anisonitrile (**3a**) in 33% yield (entry 1). We further examined other nitrogen-based bidentate ligands: 4,4′-disubstituted-2,2′-bipyridine **L2–L4** afforded **3a** in low yields (entries 2–4), whereas 1,10-phenanthroline (**L5**) drastically improved the yield of **3a** up to 91% (entry 5). Other 1,10-phenanthroline-based ligands bearing substituents at 4,7-positions, such as methyl (**L6**), methoxy (**L7**), and phenyl (**L8**), were less effective than **L5**, giving **3a** in 58%, 30%, and 59% yields, respectively (entries 6–8). Accordingly, we selected **L5** as the best ligand. Furthermore, we found that the catalytic activity was quite sensitive to the amounts of **L5**: 2 equiv. of **L5** slightly decreased the yield (86%) of **3a** and 3 equiv. of **L5** significantly suppressed the activity to 54% yield (entries 9 and 10), the latter of which was consistent with the observation that an isolated dicationic tris(1,10-phenanthroline)nickel complex, [Ni(**L5**)_3_](BF_4_)_2_, afforded **3a** in 61% yield (entry 11). Upon varying the reaction temperature at 60 °C and 100 °C, we obtained **3a** in lower yields (75% and 86% yields, respectively) compared with the reaction conducted at 80 °C, giving **3a** in 91% yield (entries 12 and 13 *vs.* entry 5). The addition of **L5** was essential: the catalyst system without **L5** gave **3a** in a low yield (10%) (entry 14). The selection of the reducing reagents is the most significant factor for the cyanation reaction: no cyanation was observed when applying typical metal-based reducing reagents, such as zinc and manganese powder, as well as a typical organic reductant, tetrakis(dimethylamino)ethylene (TDAE) (entries 15–17), and the reaction without *Si*–Me_4_-DHP did not produce **3a** (entry 18), indicating that *Si*–Me_4_-DHP was indispensable for the catalytic cyanation reaction (*vide infra*). As a result, we selected the mixture of catalytic amounts of **2** (5 mol%) and **L5** (5 mol%) in combination with an excess amount of *Si*–Me_4_-DHP (2.5 equiv.) as the best catalyst system.^12^
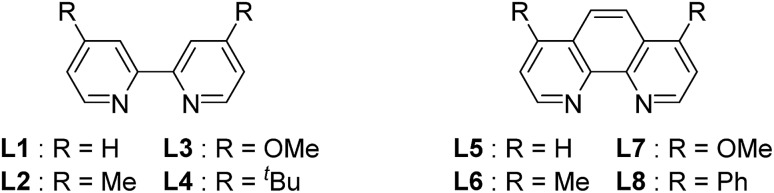


**Table 1:** Optimization for the cyanation of **1a** with acetonitrile^*a*^

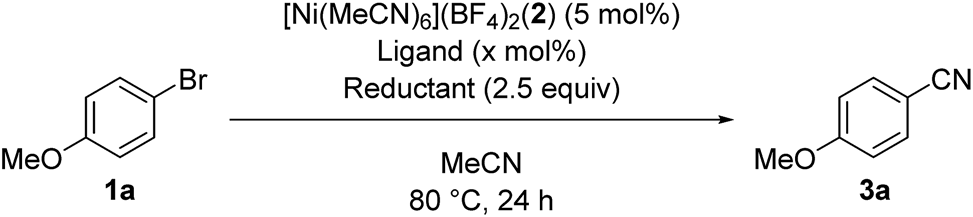			
Entry	Ligand (x mol%)	Reductant	Yield^*b*^ (%)
1	**L1** (5)	*Si*–Me_4_-DHP	33
2	**L2** (5)	*Si*–Me_4_-DHP	19
3	**L3** (5)	*Si*–Me_4_-DHP	17
4	**L4** (5)	*Si*–Me_4_-DHP	14
5	**L5** (5)	*Si*–Me_4_-DHP	91
6	**L6** (5)	*Si*–Me_4_-DHP	58
7	**L7** (5)	*Si*–Me_4_-DHP	30
8	**L8** (5)	*Si*–Me_4_-DHP	59
9	**L5** (10)	*Si*–Me_4_-DHP	86
10	**L5** (15)	*Si*–Me_4_-DHP	54
11^*c*^	—	*Si*–Me_4_-DHP	61
12^*d*^	**L5** (5)	*Si*–Me_4_-DHP	75
13^*e*^	**L5** (5)	*Si*–Me_4_-DHP	86
14	—	*Si*–Me_4_-DHP	10
15	**L5** (5)	Zn	0
16	**L5** (5)	Mn	0
17	**L5** (5)	TDAE	0
18	**L5** (5)	—	0

^*a*^Reaction conditions: **1a**, 0.10 mmol (0.025 M).

^*b*^ ^1^H NMR yield using 1,3,5-trimethoxybenzene as an internal standard.

^*c*^[Ni(**L5**)_3_](BF_4_)_2_ was used instead of **2**.

^*d*^60 °C.

^*e*^100 °C.

With the best catalyst system in hand, we examined the substrate scope of this nickel-catalyzed cyanation reaction (Table 2). Aryl bromides with electron-donating groups such as methoxy (**1a**), dialkylamino (**1b–1d**), and alkyl groups (**1e** and **1f**) at the *para*-position gave the corresponding aryl nitriles **3a–3f** in excellent to good yields (70–88%). Additionally, aryl bromides with electronically neutral *para*-substituents such as trimethylsilyl (**1g**), phenyl (**1h**), and *p*-methoxyphenyl (**1i**) were efficiently transformed into the corresponding aryl nitriles **3g–3i** in good yields (61–77%). On the other hand, aryl bromides with electron-withdrawing groups significantly decreased the yield of the corresponding aryl nitriles though the aryl bromides were completely consumed; 4-fluorobromobenzene (**1j**) afforded 4-fluorobenzonitrile (**3j**) in 36% yield together with unidentified byproducts, while aryl bromides with cyano, methoxycarbonyl, nitro, and trifluoromethyl groups resulted in a complex mixture. When the amount of *Si*–Me_4_-DHP was reduced to 1.1 equiv. for the cyanation of **1j**, **3j** was obtained in 50% yield together with 4-fluoroacetophenone (14% yield) as the main byproduct after acidic work-up. Typical protecting groups such as benzyloxycarbonyl (Cbz) and benzyl (Bn) groups were well tolerated to give the corresponding aryl nitriles in 54–86% yields (**3k–3m**). Notably, this nickel catalyst system was less sensitive to the steric hindrance around the bromide group: 4-methoxybromoarenes **1n–1p**, which respectively contained 3-Me, 2-Me, and 3,5-Me_2_ substituents, afforded the corresponding arylnitriles **3n–3p** in 65–88% yields. In addition, the cyanation of a polycyclic compound, 1-bromo-4-methoxynaphthalene (**1q**), gave **3q** in 83% yield. We further investigated the catalytic cyanation of several aryl triflates (**4a**, **4h**, **4r**, and **4s**), which were effectively converted to the corresponding aryl nitriles **3a**, **3h**, **3r**, and **3s** in 59–97% yields by increasing the catalytic loadings (15 mol%) and temperature (100 °C). Moreover, the cyanation of aryl chlorides (**5a** and **5t–5v**) afforded **3a** and **3t–3v** in 59–77% yields, whereas the cyanation of 4-iodoanisole resulted in complex mixtures.

**Table 2:** Substrate scope of substituted aryl halides and triflates^*a*^

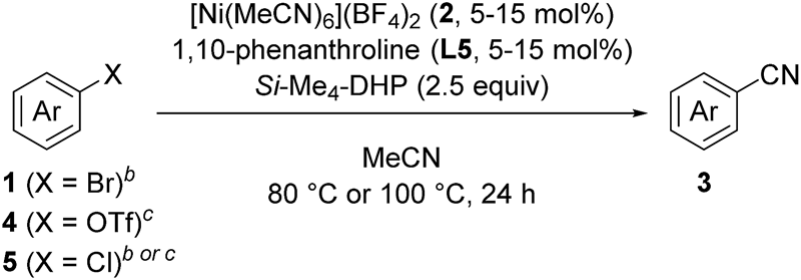
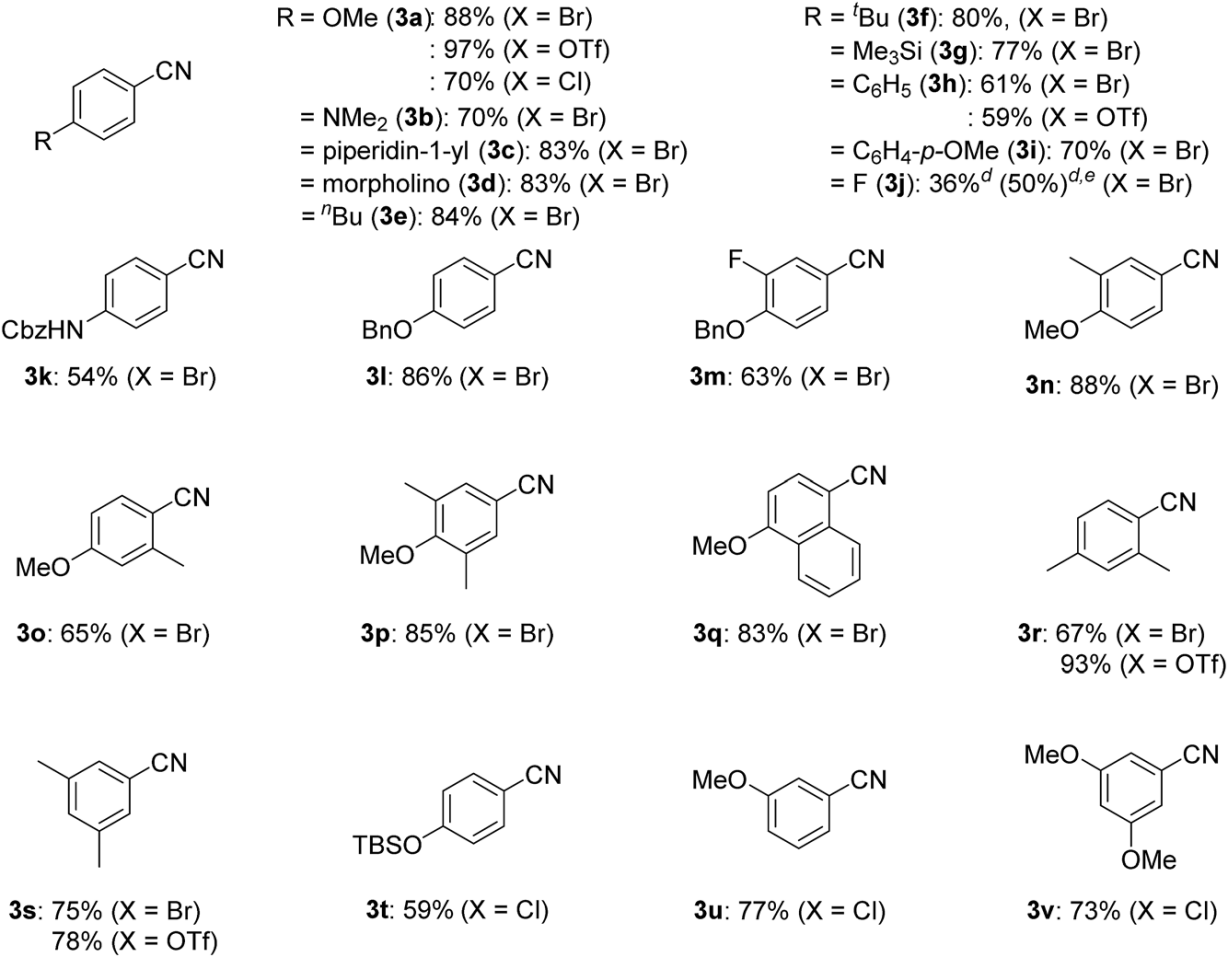

^*a*^Isolated yields.

^*b*^Reaction conditions for aryl bromides **1a–1s** and aryl chloride **5a**: **1** or **5**, 0.40 mmol (0.027 M); **2** (5 mol%); **L5** (5 mol%); 80 °C.

^*c*^Reaction conditions for aryl triflates **4a**, **4h**, **4r**, and **4s** and aryl chlorides **5t–5v**: **4** or **5**, 0.40 mmol (0.027 M); **2** (15 mol%); **L5** (15 mol%); 100 °C.

^*d*^ ^1^H NMR yield.

^*e*^ *Si*–Me_4_-DHP (1.1 equiv.) was used.

This nickel catalyst system was applicable to a wide range of heterocyclic compounds (Table 3): excellent to moderate yields were obtained for the cyanation of five- and six-membered heterocycles such as indoles (**3w–3z**) (53–99%), carbazole (**3aa**) (86%), indoline (**3ab**) (70%), dibenzofuran (**3ac**) (88%), benzothiophene (**3ad**) (48%), benzoxazole (**3ae**) (61%), pyrazole (**3af**) (52%), and pyrimidine (**3ag**) (62%). In all cases, no decomposition of the heterocycle motif was observed.

**Table 3:** Substrate scope of heterocyclic aryl bromides^*a*^

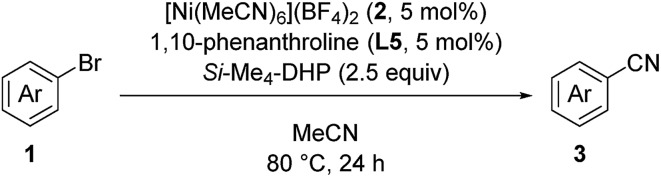
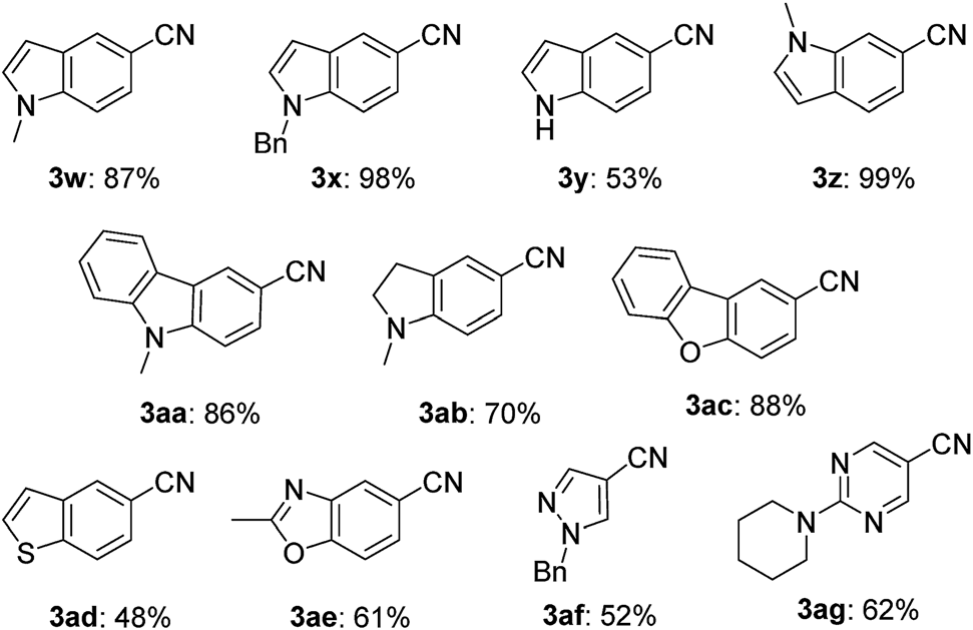

^*a*^Reaction conditions: **1**, 0.40 mmol (0.027 M). Isolated yield for all the products.

Regarding the reaction mechanism, we conducted some controlled experiments. The reaction of Ni(cod)_2_ with 4-methoxy-2-methylbromobenzene (**1o**) in the presence of 1 equiv. of **L5** afforded a nickel(ii) σ-aryl complex, Ni(C_6_H_3_-4-OMe-2-Me)Br(**L5**) (**6**), in 76% yield,^13^ which was characterized by spectral data as well as X-ray diffraction study of its single crystal. We then monitored the solution of **6** in acetonitrile with or without *Si*–Me_4_-DHP at 80 °C for 24 h: the reaction of **6** with acetonitrile in the presence of *Si*–Me_4_-DHP proceeded to give **3n** in a substantial yield (24%), whereas no reaction of **6** with acetonitrile was observed in the absence of *Si*–Me_4_-DHP or in the presence of other reducing reagents (eqn (1)), clearly indicating that complex **6** was a key intermediate and *Si*–Me_4_-DHP played a key role for giving **3o**. In fact, cyanation of **1a** in the presence of 10 mol% of **6** produced **3a** in 80% yield with stoichiometric amounts of **3o** from **6** (eqn (2)). It is noteworthy that *Si*–Me_4_-DHP was consumed in the presence of Ni(cod)_2_ (10 mol%) at room temperature to produce (Me_3_Si)_2_ and 2,3,5,6-tetramethylpyrazine (Me_4_-pyrazine).^14^ We thus assume that *Si*–Me_4_-DHP acts as a silylation reagent of nickel species.1
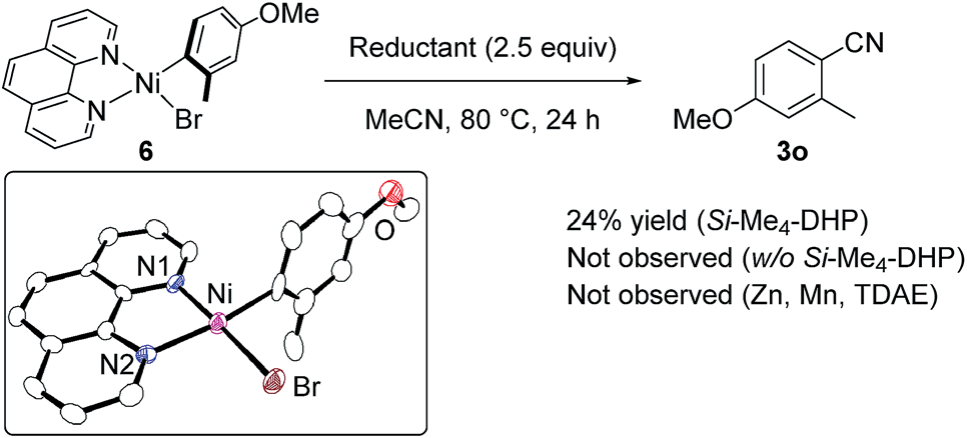

2
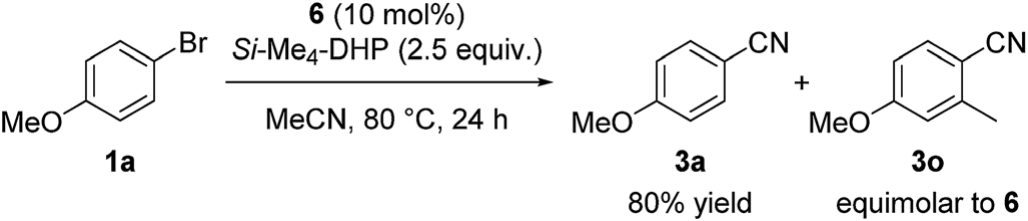



Scheme 1 shows a plausible catalytic cycle for the nickel-catalyzed cyanation reaction with acetonitrile as the sole cyano source based on the results of the controlled experiments shown in eqn (1) and (2). The initial step is the reduction of **2** by *Si*–Me_4_-DHP in the presence of **L5**, producing a 1,10-phenanthroline-coordinated Ni(0) species **A**.11*b* Subsequently, oxidative addition of aryl halides or aryl triflates (ArX) to **A** gives a Ni(ii) species, Ni(Ar)X(**L5**) (**B**), X of which is silylated by the reaction with *Si*–Me_4_-DHP to afford Ni(Ar) (SiMe_3_) (**L5**) (**C**) with a release of Me_3_SiX and Me_4_-pyrazine. Insertion of acetonitrile into the Ni–Si bond of species **C** forms *N*-(trimethylsilyl)iminoacyl species **D**.^15^ α-Methyl elimination from **D** generates isonitrile species **E**, and the subsequent 1,1-insertion of CNSiMe_3_ into the nickel-aryl moiety results in the formation of **F**. β-Silyl elimination of **F** spontaneously induces a liberation of aryl nitriles accompanied by the generation of Ni(Me) (SiMe_3_) (**L5**) (**G**).^16^ Finally, the second insertion of acetonitrile into the Ni–Si bond of **G** followed by reductive elimination of *N*-(trimethylsilyl)propan-2-imine from **H** regenerates **A**. Notably, this is consistent with the formation of 4-fluoroacetophenone after acidic work-up when aryl bromide **1j** with an electron-withdrawing substituent is used as a substrate; reductive elimination from species **D** or **F** is competitive with the formation of **G** and **3j**.

**Scheme 1:**
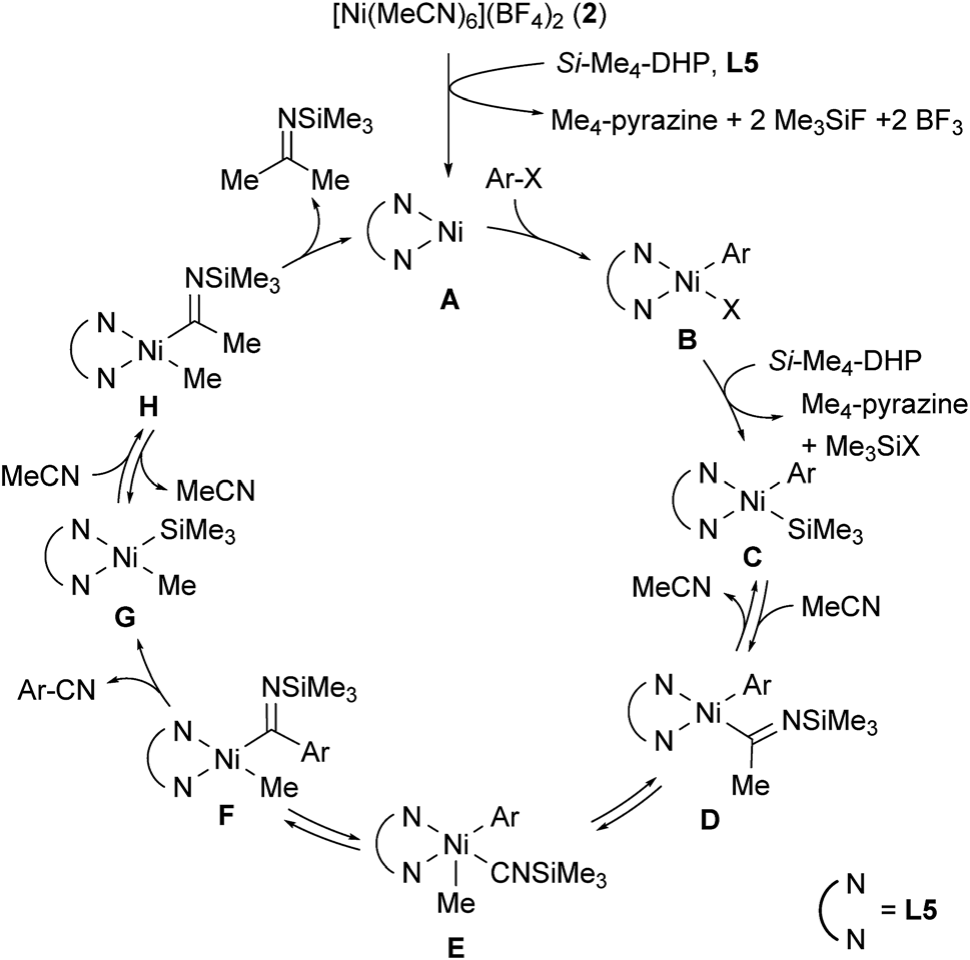
Plausible reaction mechanism.

Furthermore, we conducted the catalytic reaction in benzylnitrile under the same catalytic conditions, and we obtained a mixture of **3a** (46%), 1-benzyl-4-methoxybenzene (33%), and dibenzylketone (12%) (eqn (3)).^17^ Thus, the formation of 1-benzyl-4-methoxybenzene corresponded to reductive elimination from a similar intermediate, Ni(Ar) (Bn) (CNSiMe_3_) (**L5**) (**E′** in SI), and the formation of dibenzylketone corresponded to the formation of acetone after hydrolysis of *N*-(trimethylsilyl)propan-2-imine derived during the reaction course.3
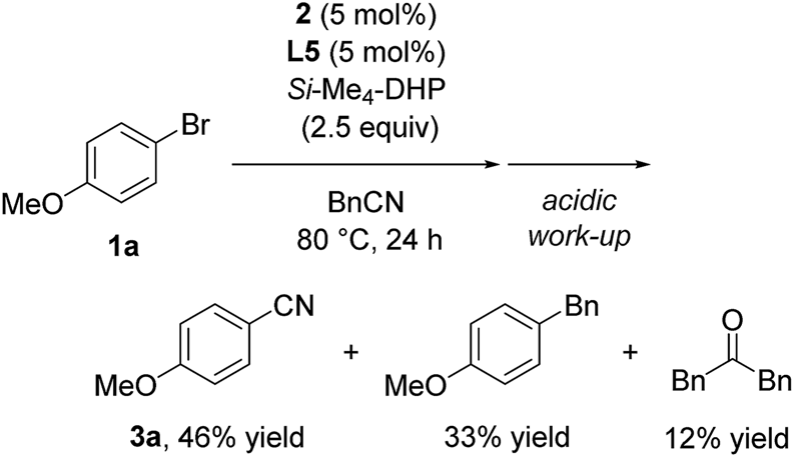



## Conclusions

We developed a toxic cyanide-free cyanation of aryl halides and aryl triflates using a nickel catalyst system of [Ni(MeCN)_6_](BF_4_)_2_ and 1,10-phenanthroline along with a stoichiometric amount of an organosilicon reductant, *Si*–Me_4_-DHP, in acetonitrile. Noteworthy was that the mechanism of our catalytic system differs from the well-established direct C–CN bond cleavage involving Lewis acid-assisted oxidative addition to electron-rich low-valent group 10 metal complexes.9g,^18^,^19^ In this catalytic system, *Si*–Me_4_-DHP had dual functions to reduce the nickel(ii) catalyst precursors but also to serve as a silylation reagent for nickel(ii) σ-aryl species. Further application of *Si*–Me_4_-DHP as a silylating reagent to transition metals is ongoing in our laboratory.

## Conflicts of interest

The authors declare no conflict of interest.

## Supplementary Material

Supplementary information

Crystal structure data
