# Supplementary material for: Nickel-catalyzed cyanation of aryl halides and triflates using acetonitrile via C–CN bond cleavage assisted by 1,4-bis(trimethylsilyl)-2,3,5,6-tetramethyl-1,4-dihydropyrazine
Source: Chem Sci. 2018 Nov 26;10(4):994–9. doi: 10.1039/c8sc04437f (PMC6349056; doi:10.1039/c8sc04437f)
Supplement: Supplementary file 1 [file SC-010-C8SC04437F-s001.pdf]

Electronic Supplementary Material (ESI) for Chemical Science.
This journal is © The Royal Society of Chemistry 2018

Supporting Information

for

Nickel-catalyzed Cyanation of Aryl Halides and Triflates Using Acetonitrile via C—CN Bond Cleavage Assisted by 1,4-Bis(trimethylsilyl)-2,3,5,6-tetramethyl-1,4-dihydropyrazine

Yohei Ueda, Nagataka Tsujimoto, Taiga Yurino, Hayato Tsurugi,* and Kazushi Mashima*

Department of Chemistry, Graduate School of Engineering Science, Osaka University, Toyonaka, Osaka, 560-8531, Japan

Contents:

1. General Information
2. Modified Procedure for Large-scale Synthesis of *Si*-Me$_4$-DHP
3. General Procedure for Catalytic Cyanation of Aryl Halides and Aryl Triflates
4. Preparation and Characterization of Ni(C_6H_3-4-OMe-2-Me)Br(L5) (6)
5. Stoichiometric Reaction of Complex 6 in the Presence of Reductants
6. Catalytic Reaction of *Si*-Me$_4$-DHP with Ni(cod)$_2$
7. Catalytic Cyanation of 4-Bromoanisole (1a) in Benzylnitrile and Its Reaction Mechanism
8. Characterization of Aryl Nitriles
9. X-Ray Crystallographic Analysis
10. ^{1}H- and ^{13}C-NMR Spectra of Aryl Nitriles
11. References

1. General Information

All manipulations for involving air- and moisture-sensitive compounds were carried out under argon atmosphere using the standard Schlenk technique and argon-filled glove box. *Si*-Me_4-DHP was prepared by the modified procedure of the literature:[S1] the reaction details for the scale-up synthesis is described in Section 2. Other organosilicon reducing reagents,[S1] $[Ni(MeCN)_6](BF_4)_2$,[S2] [Ni(**L5**)$_3$]$(BF_4)_2$,[S3] 4,7-di(9H-carbazol-9-yl)-1,10-phenanthroline,[S4] dipyrido[3,2-*a*:2',3'-*c*]phenazine,[S5] and 11,12-difluorodipyrido[3,2-*a*:2',3'-*c*]phenazine[S5] were prepared by following the literatures. The other nickel precursors ($NiCl_2$, $NiBr_2$, $Ni(acac)_2$, $Ni(OAc)_2·4H_2O$, $Ni(NO_3)_2·6H_2O$, and $Ni(cod)_2$) were purchased and used as received. Aryl halides were purchased and, if necessary, purified by distillation over CaH_2. Aryl triflates were prepared by following the literature.[S6] MeCN, toluene, hexane, diethyl ether, and THF were dried and deoxygenated by using Grubbs colomn (Glass Counter Solvent Dispending System, Nikko Hansen & Co., Ltd.). Benzylnitrile, benzene, C_6D_6, THF-d_8, acetone-d_6, and CD_3CN were distilled over CaH_2 and thoroughly degassed by trap-to-trap distillation before use. $CDCl_3$ was used as received. ^{1}H NMR spectra were measured on a Bruker AV400M (400 MHz) spectrometer at 303 K in 5 mm NMR tubes. Data were reported as follows: chemical shifts in ppm from tetramethylsilane or the residual solvent as an internal standard, integration, multiplicity (s = singlet, d = doublet, t = triplet, q = quartet, m = multiplet, dd = doublet of doublets, tt = triplet of triplets, tq = triplet of quartets, ddd = doublet of doublet of doublets, br = broad, and app = apparent), coupling constants (Hz), and assignment. ^{13}C NMR spectra were measured on a Bruker AV400M (100 MHz) spectrometer at 303 K with complete proton decoupling. Mass spectra were recorded on a JEOL JMS-700 spectrometer. GC analyses were recorded on a Shimadzu GC-2014 gas chromatograph with J&W Scientific DB-5 column (Length: 30 m, Diam.: 0.250 mm). The elemental analysis was recorded by using Perkin Elmer 2400 at the Faculty of Engineering Science, Osaka University. Flash column chromatography was performed using silica gel 60 (0.040—0.063 mm, 230—400 mesh ASTM).

2. Modified Procedure for Large-scale Synthesis of *Si*-Me$_4$-DHP

Caution: All manipulations should be conducted under inert atmosphere.

In an oven-dried 500 mL round-bottom flask, freshly cutted potassium pieces (15.8 g, 0.404 mol) was suspended in THF (150 mL). Chlorotrimethylsilane (51.3 mL, 0.404 mol) was added to the THF suspension. In a 200 mL Schlenk, 2,3,5,6-tetramethylpyrazine (18.4 g, 0.135 mol) was dissolved in THF (100 mL), and transferred slowly to the THF suspension containing potassium pieces using cannula at rt. After stirring for 1 week, all the solvent was removed under vacuum. The residue was suspended in hexane (100 mL), filtered through Celite®, then extracted with hexane (50 mL×4). The filtrate was concentrated, giving the target compound, *Si*-Me$_4$-DHP as a white crystalline solid in 78% yield (29.8 g). The Celite® pad must be carefully quenched due to the small contamination of potassium.

3. General Procedure for Catalytic Cyanation of Aryl Halides and Aryl Triflates

3-1. Optimization of the Reaction Conditions

To a solution of 4-bromoanisole (**1a**) (18.7 mg, 0.100 mmol), nickel precursor (5.00 μmol) and ligand in MeCN (4.00 mL), was added 2.5 equiv of *Si*-Me$_4$-DHP (70.6 mg, 0.250 mmol) in a 30 mL Schlenk. This solution was stirred at 80 ºC for 24 h, and quenched by adding EtOAc (1.00 mL). The obtained mixture was concentrated under vacuum and poured onto short pat of Celite® with EtOAc, and collected eluent was concentrated. The ^{1}H NMR yield was determined by the measurement of the obtained crude mixture in $CDCl_3$ using 1,3,5-trimethoxybenzene as an internal standard.

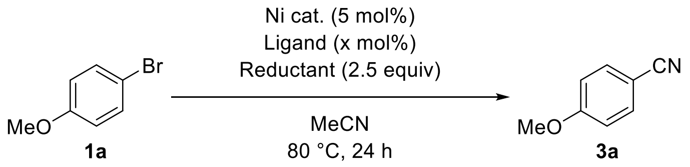

Table S1. Screening of Ligands

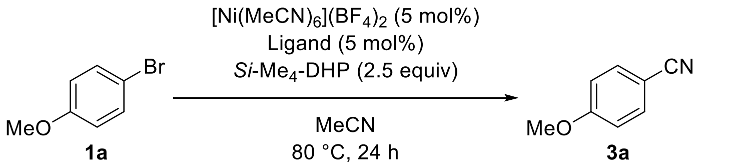

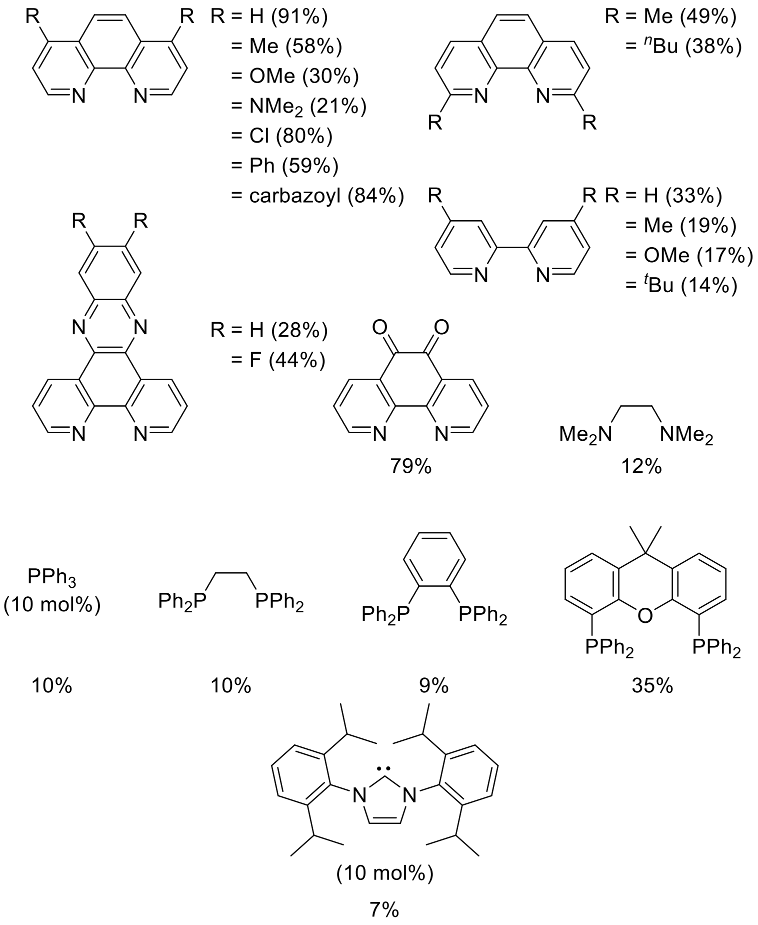

Reaction conditions: **1a**; 0.10 mmol (0.025 M), *Si*-Me$_4$-DHP; 0.25 mmol.
^{1}H NMR yields using 1,3,5-trimethoxybenzene as an internal standard.

Table S2. Screening of Nickel Catalysts

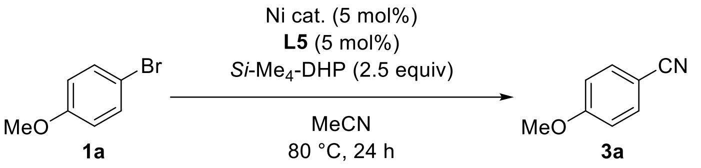

Entry	Ni cat.	Yield (%)[a]
1	$[Ni(MeCN)_6](BF_4)_2$	91
2	$NiCl_2$	37
3	$NiBr_2$	15
4	$Ni(acac)_2$	81
5	$Ni(OAc)_2·4H_2O$	0
6	$Ni(NO_3)_2·6H_2O$	38
7	$Ni(cod)_2$	68

Reaction conditions: **1a**; 0.10 mmol (0.025 M), reductant; 0.25 mmol.
[a] ^{1}H NMR yields using 1,3,5-trimethoxybenzene as an internal standard.

Table S3. Screening of Reductants

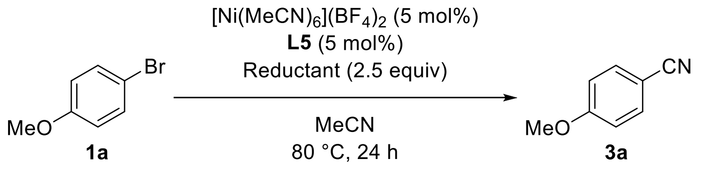

Entry	Reductant	Yield (%)[a]
1	*Si*-Me$_4$-DHP	91
2	*Si*-Me$_2$-DHP	21
3	*Si*-DHP	0
4	*Si*-DHBP	0
5	Mn	0
6	Zn	0
7	TDAE	0

Reaction conditions: **1a**; 0.10 mmol (0.025 M), reductant; 0.25 mmol.
[a] ^{1}H NMR yield using 1,3,5-trimethoxybenzene as an internal standard.

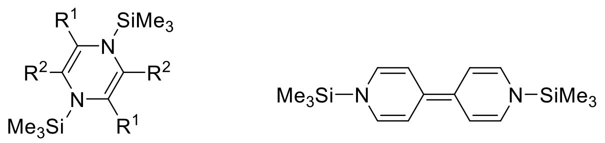

Si-Me$_4$-DHP : R^1 = R^2 = Me
Si-Me$_2$-DHP : R^1 = Me, R^2 = H
Si-DHP : R^1 = R^2 = H

Si-DHBP

Table S4. Effect of Catalyst Concentration

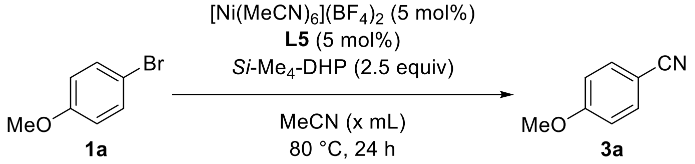

Entry	MeCN (x mL)	Yield (%)[a]
1	2.0	80
2	3.0	84
3	4.0	91
4	5.0	81

Reaction conditions: **1a**; 0.10 mmol, *Si*-Me_4-DHP; 0.25 mmol.

[a] ^{1}H NMR yield using 1,3,5-trimethoxybenzene as an internal standard.

3-2. Substrate Scope of Catalytic Cyanation of Aryl Halides and Aryl Triflates

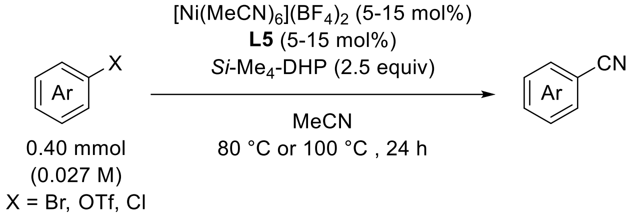

[Condition A for Aryl Bromides 1]

To a solution of aryl bromides (**1**) (0.400 mmol), $[Ni(MeCN)_6](BF_4)_2$ (**2**) (0.0200 mmol) and **L5** (0.0200 mmol) in MeCN (15.0 mL) was added 2.5 equiv of *Si*-Me_4-DHP (282.6 mg, 1.00 mmol) in a 80 mL Schlenk. This solution was stirred at 80 ºC for 24 h, and then quenched by adding EtOAc (5.00 mL). The obtained crude mixture was concentrated under vacuum. The nitrile product was purified by silica gel flush column chromatography. All nitrile compounds were known, and their NMR data were superimposed to the literature.

Cyanation of electron-deficient bromoarenes listed in Table S5 was unsuccessful because of the undesired side reactions.

Table S5. Limitations of Substrate Scope

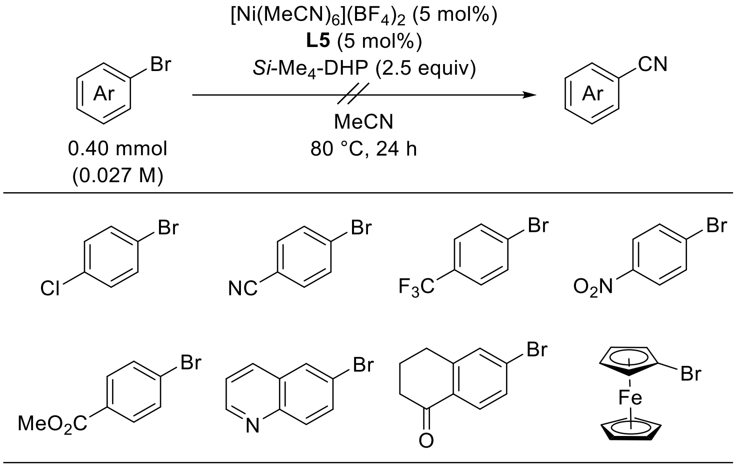

[Condition B for Aryl Triflates 4]

To a solution of aryl triflate (**4**) (0.400 mmol), $[Ni(MeCN)_6](BF_4)_2$ (**2**) (0.0600 mmol, 15 mol% to **4**) and **L5** (0.0600 mmol, 15 mol% to **4**) in MeCN (15.0 mL) was added 2.5 equiv of *Si*-Me_4-DHP (282.6 mg, 1.00 mmol) in a 80 mL Schlenk. This solution was stirred at 100 ºC for 24 h, and then quenched by adding EtOAc (5.00 mL). The obtained crude mixture was concentrated under vacuum. The nitrile product was purified by silica gel flush column chromatography. All nitrile compounds were known, and their NMR data were superimposed to the literature.

[Condition C for Aryl Chlorides 5]

For 4-chloroanisole (**5a**), the reaction was conducted under the same conditions for aryl bromides (Condition A). For other aryl chlorides (**5t**—**5v**), the reactions were conducted under the same conditions for aryl triflates (Condition B)

4. Preparation and Characterization of Ni(C_6H_3-4-OMe-2-Me)Br(L5) (6)

To a yellow solution of Ni(cod)$_2$ (600 mg, 2.18 mmol) in toluene (15.0 mL), were added 1,10-phenanthroline (432 mg, 2.40 mmol, 1.1 equiv) and 4-methoxy-2-methylbromobenzene (2.10 mL, 15.0 mmol, 6.9 equiv) at - 40 °C. The reaction mixture was gradually warmed up to rt and stirred for 10 h. The resulting dark purple suspension was washed with toluene (10.0 mL) for three times, with diethyl ether (10.0 mL), and hexane (10.0 mL). Drying the purple precipitates *in vacuo* gave complex **6** as purple powders (693 mg, 76% yield). Single crystals of **6** suitable for X-ray diffraction study were obtained by vapor diffusion of hexane into a concentrated THF / benzene solution at rt. Because of low solubility of complex **6** in THF-d_8, benzene-d_6, acetone-d_6 (solubility: ~1.0 mg in 0.5 mL of solvent), resonances for ^{13}C NMR were not clearly detected.

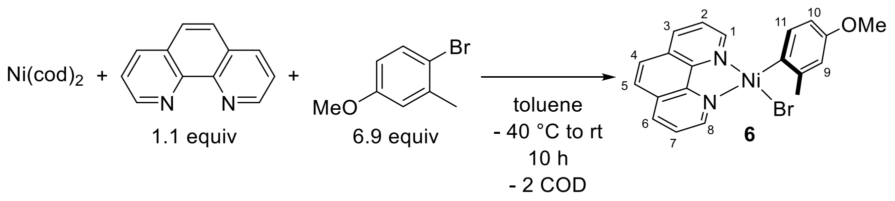

mp 140 °C (decompose).

^{1}H NMR (400 MHz, THF-*d*$_8$) 9.71 (d, ^3J(H^1—H^2) = 3.7 Hz, 1H, H^1), 8.60 (d, ^3J(H^3—H^2) = 8.5 Hz, 1H, H^3), 8.55 (d, ^3J(H^6—H^7) = 7.0 Hz, 1H, H^6), 8.04 (d, ^3J(H^4—H^5) = 8.5 Hz, 1H, H^5), 7.99 (d, ^3J(H^5—H^4) = 8.5 Hz, 1H, H^5), 7.92 (app t, 1H, H^2), 7.59 (app t, 1H, H^7), 7.58 (s, 1H, H^8), 7.36 (d, ^3J(H^{11}—H^{10}) = 8.2 Hz, 1H, H^{11}), 6.51 (s, 1H, H^9), 6.48 (d, ^3J(H^{10}—H^{11}) = 8.2 Hz, 1H, H^{10}), 3.87 (s, 3H, OMe), 3.00 (s, 3H, Me).

Anal. Calcd for $C_{20}H_{17}N_2OBrNi$: C, 54.60; H, 3.89; N, 6.37. Found: C, 53.84; H, 3.76; N, 6.77. We were unable to obtain reasonable elemental analyses because of contamination by nickel black.

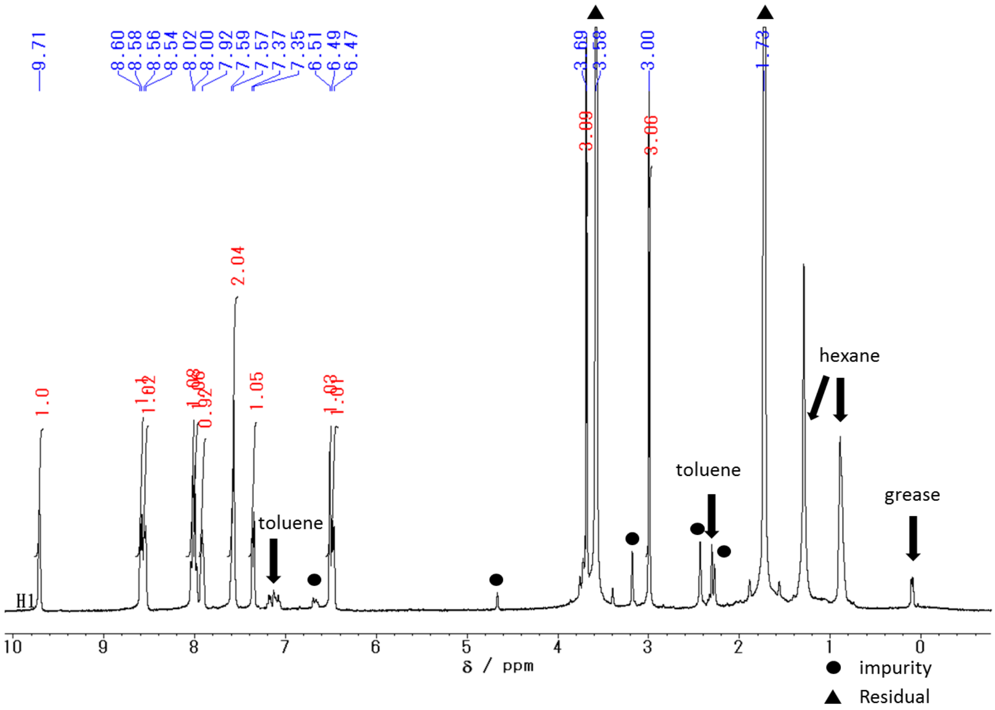

Figure S1. ^{1}H NMR spectrum of complex **6** in THF-d_8.

5. Stoichiometric Reaction of Complex 6 in the Presence of Reductants

To a solution of complex **6** (22.0 mg, 50 μmol) in MeCN (4.00 mL), was added a reductant (2.5 equiv) in a 30 mL Schlenk. This mixture was stirred at 80 °C for 24 h, and quenched by adding EtOAc(1.0 mL). ^{1}H NMR yield was determined by adding 1,3,5-trimethoxybenzene as an internal standard.

Table S6. Stoichiometric Reaction of Complex **6** in the Presence of Reductants

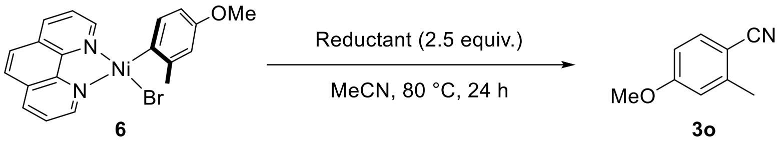

6 → 3o

Entry	Reductant	Yield[a] [%]
1	1	24
2	Zn	0
3	Mn	0
4	TDAE	0
5	-	0

[a] ^{1}H NMR yields using 1,3,5-trimethoxybenzene as an internal standard.

6. Catalytic Reaction of *Si*-Me$_4$-DHP with Ni(cod)$_2$

To a solution of *Si*-Me$_4$-DHP (8.5 mg, 30 μmol) and hexamethylbenzene (1.0 mg, 6.2 μmol) in CD_3CN (0.50 mL), was added catalytic amount of Ni(cod)$_2$ (0.8 mg, 3 μmol). This solution was stand at rt for 24 h, leading to the decomposition of *Si*-Me$_4$-DHP to give hexamethyldisilane (39% ^{1}H NMR yield) and Me$_4$-pyrazine (39% ^{1}H NMR yield). *Si*-Me$_4$-DHP was stable in CD_3CN at 80 °C in the absence of Ni(cod)$_2$.

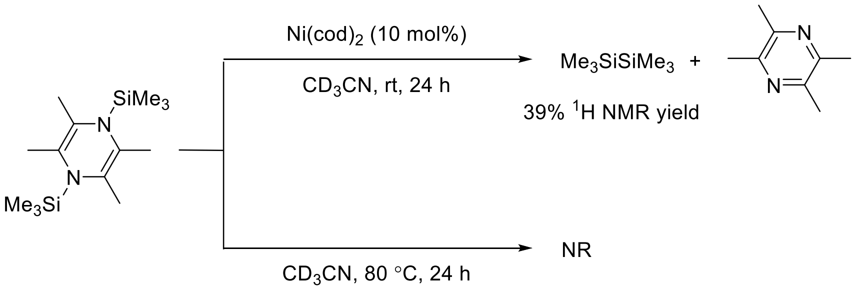

7. Catalytic Cyanation of 4-Bromoanisole (1a) in Benzylnitrile and Its Reaction Mechanism

To a solution of 4-bromoanisole (**1a**) (18.7 mg, 0.100 mmol), [Ni(MeCN)$_6$](BF$_4$)$_2$ (**2**) (5.00 µmol) and **L5** (5.00 µmol) in benzylnitrile (4.00 mL) was added 2.5 equiv of *Si*-Me$_4$-DHP (70.6 mg, 0.250 mmol) in a 30 mL Schlenk. This solution was stirred at 80 °C for 24 h, and quenched by adding EtOAc (1.0 mL). GC yield was determined by the measurement of the crude mixture using pentadecane as an internal standard. In this mixture, we found **3a**, 1-benzyl-4-methoxybenzene, and dibenzylketone. The ratio of **3a** (46%), 1-benzyl-4-methoxybenzene (33%), and dibenzylketone (12%) is rationally explained by Scheme S1. In this cycle, benzylnitrile further reacts with **G'** to afford **H'**, from which 12% of dibenzylketone is formed via reductive elimination from **H'** and subsequent hydrolysis. Additionaly, 33% of 1-benzyl-4-methoxybenzene is formed by the reductive elimination from **E'** to afford **A** with a release of 33% of trimethylsilylisocyanide. Trimethylsilylisocyanide also serves as cyanide source by the reaction with **B**, giving 33% of **3a** with a recovery of **A**. Total amount of **3a** (46%) is consistent with the reaction mechanism as shown in Scheme S1.

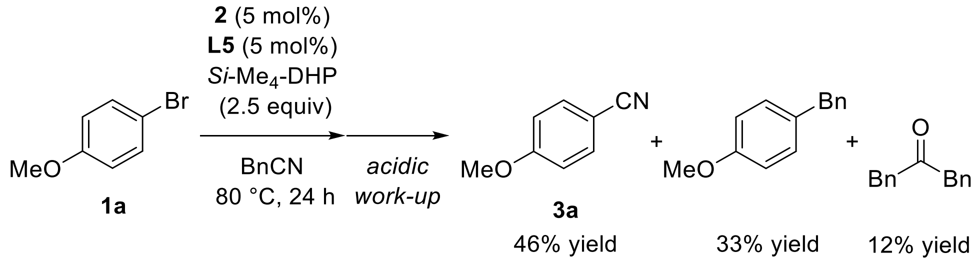

Scheme S1. Reaction Mechanism of Ni-catalyzed Cyanation of **1a** in Benzylnitrile

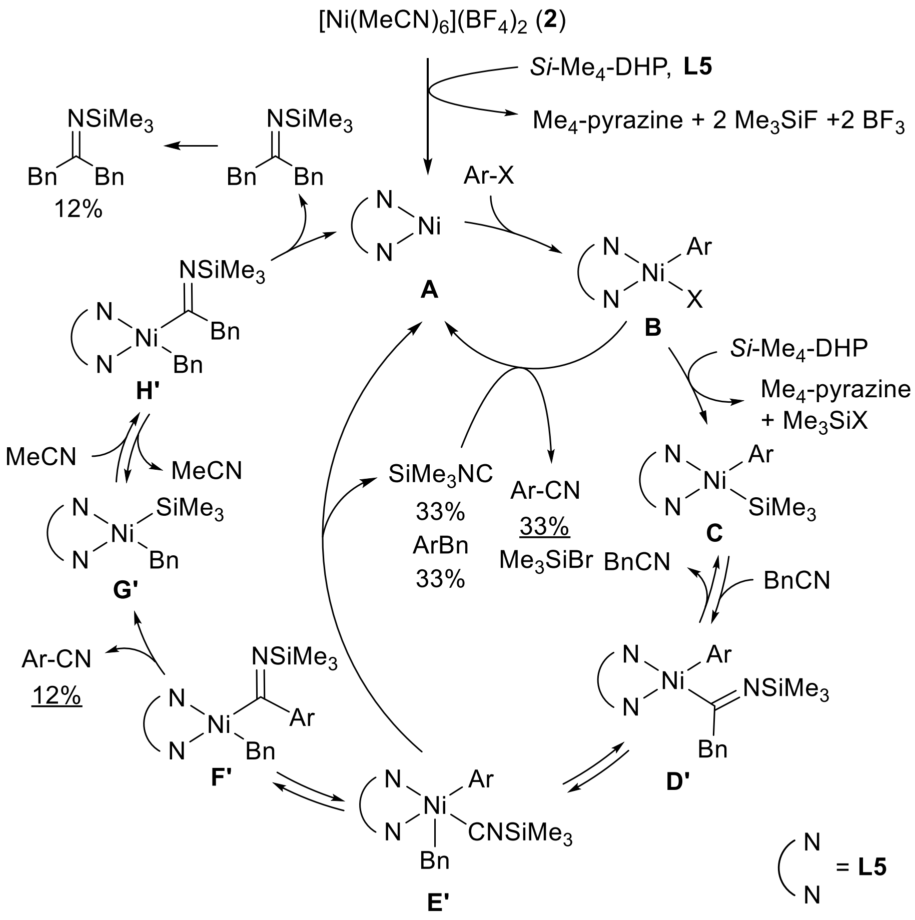

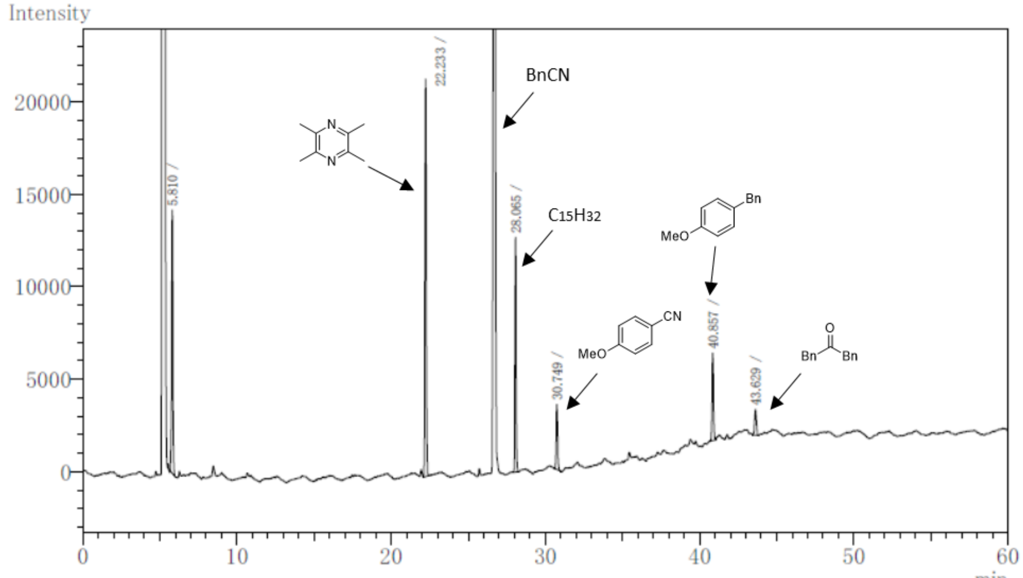

Peak No. ピーク番号	Retention time 保持時間	Area 面積	Height 高さ	Concentration 濃度	単位 Unit	Mark マーク	ID#	Chemical Name 化合物名
1	5.810	80270	14211	23.577				
2	22.233	128303	21469	37.686				
3	28.065	66137	12681	19.426				
4	30.749	23295	3519	6.842				
5	40.857	31721	4735	9.317				
6	43.629	10729	1389	3.151				
合計 Sum		340455	58004					

Figure S2. GC spectrum for the reaction mixture in benzylnitrile using pentadecane as an internal standard.

8. Characterization of Aryl Nitriles

anisonitrile (3a; CAS: 874-90-8) [S8]

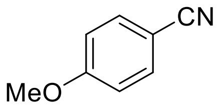

Isolated as white powder (46.9 mg, 88% yield (condition A); 51.8 mg, 97% yield (condition B); 37.3 mg, 70% yield (condition C)).

^{1}H-NMR (400 MHz, $CDCl_3$): δ 7.56 (d, *J* = 8.8 Hz, 2H), 6.93 (d, *J* = 8.8 Hz, 2H), 3.84 (s, 3H).

^{13}C-NMR (100 MHz, $CDCl_3$): δ 163.0, 134.0, 119.3, 114.8, 104.0, 55.6.

4-(dimethylamino)benzonitrile(3b; CAS: 1197-19-9)[S9]

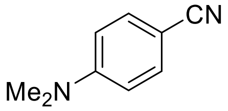

Isolated as white powder (40.7 mg, 70% yield (condition A)).

^{1}H-NMR (400 MHz, $CDCl_3$): δ 7.44 (d, *J* = 8.7 Hz, 2H), 6.62 (d, *J* = 8.7 Hz, 2H), 3.02 (s, 6H).

^{13}C-NMR (100 MHz, $CDCl_3$): δ 152.6, 133.4, 120.8, 111.5, 97.4, 40.0.

4-(piperidin-1-yl)benzonitrile (3c)[S10]

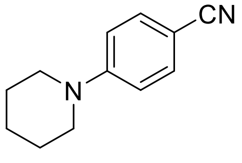

Isolated as pale-yellow powder (61.5 mg, 83% yield (condition A)).

^{1}H-NMR (400 MHz, $CDCl_3$): δ 7.43 (d, *J* = 8.2 Hz, 2H), 6.81 (d, *J* = 8.2 Hz, 2H), 3.31 (app s, 4H), 1.64 (app s, 6H).

^{13}C-NMR (100 MHz, $CDCl_3$): δ 153.6, 133.5, 120.4, 114.1, 98.9, 48.4, 25.3, 24.3.

HRMS (EI) (*m*/*z*): $[M]^+$ calced. for $C_{12}H_{14}N_2$, 186.1157; found, 186.1150.

4-morpholinobenzonitrile (3d)[S10]

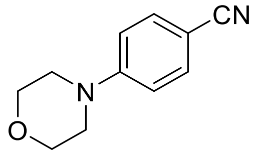

Isolated as yellow oil (66.3 mg, 88% yield (condition A)).

^{1}H-NMR (400 MHz, $CDCl_3$):δ 7.50 (d, *J* = 8.4 Hz, 2H), 6.85 (d, *J* = 8.4 Hz, 2H), 3.88-3.82 (m, 4H), 3.30-3.24 (m, 4H).

^{13}C-NMR (100 MHz, $CDCl_3$):δ 153.6, 133.6, 120.0, 114.2, 101.1, 66.6, 47.4.

HRMS (EI) (*m*/*z*): [M]$^+$ calced. for $C_{11}H_{12}N_2O$, 188.0950; found, 188.0949.

4-butylbenzonitrile (3e)[S10]

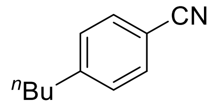

Isolated as colorless oil (53.5 mg, 84% yield (condition A)).

^{1}H-NMR (400 MHz, $CDCl_3$ (TMS)):δ 7.56 (d, *J* = 8.1 Hz, 2H), 7.27 (d, *J* = 8.1 Hz, 2H), 2.66 (t, *J* = 7.6 Hz, 2H), 1.61 (tt, *J* = 7.6 Hz, 7.5 Hz, 2H), 1.36 (tq, *J* = 7.5 Hz, 7.4 Hz, 2H), 0.93 (t, *J* = 7.4 Hz, 3H).

^{13}C-NMR (100 MHz, $CDCl_3$ (TMS)):δ 148.7, 132.2, 129.3, 119.3, 109.7, 35.9, 33.2, 22.4, 14.0.

HRMS (EI) (*m*/*z*): [M]$^+$ calced. for $C_{11}H_{13}N$, 159.1048; found, 159.1046.

4-(*tert*-butyl)benzonitrile (3f; CAS: 4210-32-6)[S10]

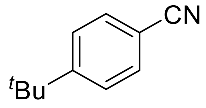

Isolated as pale-yellow oil (50.9 mg, 80% yield (condition A)).

^{1}H-NMR (400 MHz, $CDCl_3$):δ 7.58 (d, *J* = 8.4 Hz, 2H), 7.48 (d, *J* = 8.4 Hz, 2H), 1.32 (s, 9H).

^{13}C-NMR (100 MHz, $CDCl_3$):δ 156.7, 132.0, 126.2, 119.2, 109.4, 35.4, 31.0.

4-(trimethylsilyl)benzonitrile (3g)[S11]

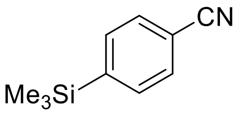

Isolated as pale-yellow oil (53.8 mg, 77% yield (condition A)).

^{1}H-NMR (400 MHz, $CDCl_3$):δ 7.67-7.55 (app s, 4H), 0.29 (s, 9H).

^{13}C-NMR (100 MHz, $CDCl_3$):δ 147.4, 133.9, 131.0, 119.1, 112.5, -1.4.

HRMS (EI) (*m/z*): [M]$^+$ calced. for $C_{14}H_{11}NO$, 209.0841; found, 209.0839.

4-cyano-1,1'-biphenyl (3h; CAS: 2920-38-9)[S8]

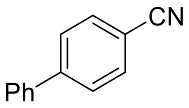

Isolated as white powder (43.4 mg, 61% yield (condition A); 42.3 mg,59% yield (condition B)).

^{1}H-NMR (400 MHz, $CDCl_3$):δ 7.73 (d, *J* = 8.4 Hz, 2H), 7.68 (d, *J* = 8.4 Hz, 2H), 7.59 (app d, *J* = 7.0 Hz, 2H), 7.49 (app t, *J* = 7.0 Hz, 2H), 7.43 (app t, *J* = 7.1 Hz, 1H).

^{13}C-NMR (100 MHz, $CDCl_3$):δ 145.8, 139.3, 132.7, 129.2, 128.8, 127.9, 127.4, 119.1, 111.1.

4-cyano-4'-methoxy-1,1'-biphenyl (3i)[S12]

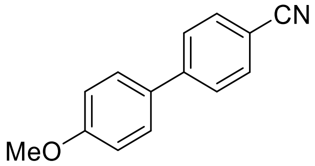

Isolated as white powder (58.5 mg, 70% yield (condition A)).

^{1}H-NMR (400 MHz, $CDCl_3$):δ 7.69 (d, *J* = 8.2 Hz, 2H,), 7.64 (d, *J* = 8.2 Hz, 2H), 7.54 (d, *J* = 8.6 Hz, 2H) 7.01 (d, *J* = 8.6 Hz, 2H), 3.86 (s, 3H).

^{13}C-NMR (100 MHz, $CDCl_3$):δ 160.4, 145.4, 132.7, 131.6, 128.5, 127.2, 119.2, 114.7, 110.3, 55.5.

HRMS (EI) (*m/z*): [M]$^+$ calced. for $C_{10}H_{11}NO$, 161.0841; found, 161.0840.

benzyl (4-cyanophenyl)carbamate (3k)[S13]

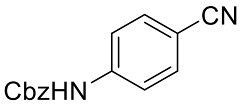

Isolated as pale-yellow powder (54.4 mg, 54% yield (condition A)).

^{1}H-NMR (400 MHz, CDCl$_3$):δ 7.58 (d, J = 8.8 Hz, 2H), 7.51 (d, J = 8.8 Hz, 2H), 7.40-7.35 (m, 5H), 6.92 (br s, 1H), 5.22 (s, 2H).

^{13}C-NMR (100 MHz, CDCl$_3$):δ 152.8, 142.1, 135.6, 128.9, 128.8, 128.6, 119.0, 118.5, 106.6, 67.8.

HRMS (EI) (*m*/*z*): [M]$^+$ calced. for $C_{15}H_{12}N_2O_2$, 252.0899; found, 252.0900.

4-(benzyloxy)benzonitrile (3l; CAS: 52805-36-4)[S8]

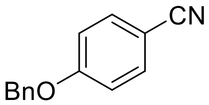

Isolated as white powder (71.9 mg, 86% yield (condition A)).

^{1}H-NMR (400 MHz, CDCl$_3$):δ 7.58 (d, J = 8.0 Hz, 2H), 7.41-7.30 (m, 5H), 7.02 (d, J = 8.0 Hz, 2H), 5.12 (s, 2H).

^{13}C-NMR (100 MHz, CDCl$_3$):δ 162.0, 135.8, 134.0, 128.8, 128.4, 127.5, 119.2, 115.6, 104.3, 70.3.

4-(benzyloxy)-3-fluorobenzonitrile (3m)[S10]

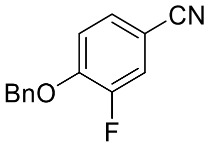

Isolated as white powder (57.0 mg, 63% yield (condition A)).

^{1}H-NMR (400 MHz, CDCl$_3$):δ 7.43-7.32 (m, 7H), 7.05 (t, J = 8.3 Hz, 1H), 5.21 (s, 2H).

^{13}C-NMR (100 MHz, CDCl$_3$):δ 152.2 (d, $J_{C\text{-}F}$ = 249.0 Hz), 151.0 (d, $J_{C\text{-}F}$ = 10.2 Hz), 135.3, 129.7 (d, $J_{C\text{-}F}$ = 3.6 Hz), 129.0, 128.7, 127.5, 119.9 (d, $J_{C\text{-}F}$ = 21.1 Hz), 118.1 (d, $J_{C\text{-}F}$ = 2.7 Hz), 115.5 (d, $J_{C\text{-}F}$ = 2.2 Hz), 104.5 (d, $J_{C\text{-}F}$ = 8.2 Hz), 71.4.

HRMS (EI) (*m*/*z*): [M]$^+$ calced. for $C_{14}H_{10}OF$, 227.0746; found, 227.0743.

4-methoxy-3-methylbenzonitrile (3n)[S14]

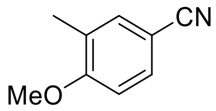

Isolated as pale-yellow powder (51.8 mg, 88% yield (condition A)).

^{1}H-NMR (400 MHz, CDCl$_3$):δ 7.46 (d, J = 8.1 Hz, 1H) 7.37 (s, 1H), 6.83 (d, J = 8.1 Hz, 1H), 3.86 (s, 3H), 2.19 (s, 3H).

^{13}C-NMR (100 MHz, CDCl$_3$):δ 161.2, 133.9, 132.0, 128.2, 119.5, 110.2, 103.4, 55.6, 16.0.

HRMS (EI) (*m*/*z*): [M]$^+$ calced. for C_9H_9NO, 147.0684; found, 147.0680.

4-methoxy-2-methylbenzonitrile (3o)[S14]

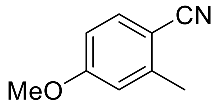

Isolated as yellow powder (38.2 mg, 65% yield (condition A)).

^{1}H-NMR (400 MHz, CDCl$_3$):δ 7.49 (d, J = 8.6 Hz, 1H, Ph) 6.79 (s, 1H), 6.76 (d, J = 8.6 Hz, 1H, Ph), 3.82 (s, 3H), 2.49 (s, 3H).

^{13}C-NMR (100 MHz, CDCl$_3$):δ 162.8, 144.1, 134.2, 118.6, 115.8, 112.2, 104.6, 55.5, 20.7.

HRMS (EI) (*m*/*z*): [M]$^+$ calced. for C_9H_9NO, 147.0684; found, 147.0681.

4-methoxy-3,5-dimethylbenzonitrile (3p)[S14]

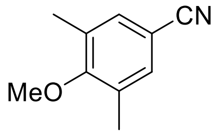

Isolated as pale-yellow powder (54.9 mg, 85% yield (condition A)).

^{1}H-NMR (400 MHz, CDCl$_3$):δ 7.29 (s, 2H), 3.74 (s, 3H), 2.28 (s, 6H).

^{13}C-NMR (100 MHz, CDCl$_3$):δ 160.9, 132.8, 132.6, 119.1, 107.4, 59.4, 16.0.

HRMS (EI) (*m*/*z*): [M]$^+$ calced. for $C_{10}H_{11}NO$, 161.0841; found, 161.0840.

4-methoxy-1-naphthonitrile (3q)[S15]

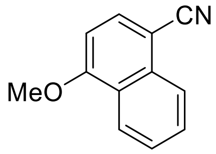

Isolated as white powder (61.2 mg, 83% yield (condition A)).

^{1}H-NMR (400 MHz, CDCl$_3$):δ 8.32 (d, J = 8.4 Hz, 1H), 8.17 (d, J = 8.4 Hz, 1H), 7.85 (d, J = 8.1 Hz, 1H), 7.69 (app t, J = 7.5 Hz, 1H), 7.58 (app t, J = 7.5 Hz, 1H), 6.83 (d, J = 8.1 Hz, 1H), 4.07 (s, 3H).

^{13}C-NMR (100 MHz, CDCl$_3$):δ 159.6, 134.2, 133.7, 129.1, 126.9, 125.4, 125.1, 122.9, 118.6, 103.5, 102.1, 56.1.

HRMS (FAB) (*m*/*z*): [M+H]$^+$ calced. for $C_{12}H_{10}ON$, 184.0762; found, 184.0758.

2,4-dimethylbenzonitrile (3r)[S10]

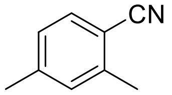

Isolated as white powder (35.4 mg, 67% yield (condition A); 48.8 mg, 93% yield (condition B)).

^{1}H-NMR (400 MHz, CDCl$_3$):δ 7.47 (d, J = 7.6 Hz, 1H), 7.12 (s, 1H), 7.06 (d, J = 7.6 Hz, 1H), 2.50 (s, 3H), 2.37 (s, 3H).

^{13}C-NMR (100 MHz, CDCl$_3$):δ 143.6, 141.8, 132.5, 131.1, 127.1, 118.5, 109.8, 21.8, 20.4.

HRMS (EI) (*m*/*z*): [M]$^+$ calced. for C_9H_9N, 133.0735; found, 133.0731.

3,5-dimethylbenzonitrile (3s)[S16]

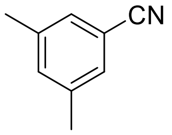

Isolated as white powder (40.0 mg, 75% yield (condition A); 41.6 mg, 78% yield (condition B)).

^{1}H-NMR (400 MHz, CDCl$_3$ (TMS)):δ 7.26 (s, 2H), 7.21 (s, 1H), 2.34 (s, 6H).

^{13}C-NMR (100 MHz, CDCl$_3$ (TMS)):δ 139.2, 134.7, 129.8, 119.3, 112.2, 21.1.

HRMS (EI) (*m*/*z*): [M]$^+$ calced. for C_9H_9N, 133.0735; found, 133.0734.

4-((*tert*-butyldimethylsilyl)oxy)benzonitrile (3t)[S17]

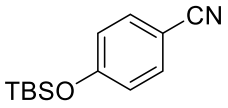

Isolated as colorless oil (55.0 mg, 59% yield (condition C)).

^{1}H-NMR (400 MHz, $CDCl_3$):δ 7.54 (d, J = 8.5 Hz, 2H), 6.88 (d, J = 8.5 Hz, 2H), 0.98 (s, 9H), 0.23 (s, 6H).

^{13}C-NMR (100 MHz, $CDCl_3$):δ 159.8, 134.1, 121.0, 119.3, 108.4, 25.6, 18.3, 4.3.

HRMS (FAB) (*m*/*z*): [M+H]$^+$ calced. for $C_{13}H_{20}NOSi$, 234.1314; found, 234.1313.

3-methoxylbenzonitrile (3u; CAS: 1527-89-5)[S18]

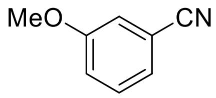

Isolated as white powder (40.9 mg, 77% yield (condition C)).

^{1}H-NMR (400 MHz, $CDCl_3$ (TMS)):δ 7.39-7.35 (m, 1H), 7.25-7.22 (m, 1H), 7.15-7.11 (m, 2H), 3.83 (s, 3H).

^{13}C-NMR (100 MHz, $CDCl_3$ (TMS)):δ 159.8, 130.4, 124.6, 119.4, 118.8, 117.0, 113.4, 55.6.

3,5-dimethylbenzonitrile (3v; CAS: 19179-31-8)[S10]

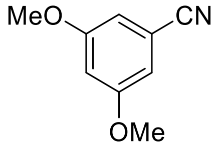

Isolated as white powder (47.6 mg, 73% yield (condition C))

^{1}H-NMR (400 MHz, $CDCl_3$):δ 6.76 (s, 2H), 6.65 (s, 1H), 3.81 (s, 6H).

^{13}C-NMR (100 MHz, $CDCl_3$):δ 161.2, 118.8, 113.6, 110.1, 105.8, 55.8.

1-methyl-*1H*-indole-5-carbonitrile (3w)[S10]

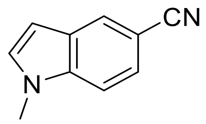

Isolated as white powder (54.3 mg, 87% yield (condition A)).

^{1}H-NMR (400 MHz, CDCl$_3$):δ 7.96 (s, 1H), 7.44 (app d, *J* = 8.5 Hz, 1H), 7.36 (d, *J* = 8.5 Hz, 1H), 7.17 (d, *J* = 3.1 Hz, 1H), 6.57 (d, *J* = 3.1 Hz, 1H), 3.83 (s, 3H).

^{13}C-NMR (100 MHz, CDCl$_3$):δ 138.3, 131.2, 128.3, 126.6, 124.6, 121.0, 110.1, 102.6, 102.3, 33.2.

HRMS (EI) (*m*/*z*): [M]$^+$ calced. for $C_{10}H_8N_2$, 156.0687; found, 156.0684.

1-benzyl-1H-indole-5-carbonitrile (3x)[S15]

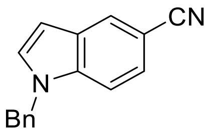

Isolated as pale-brown powder (91.6 mg, 98% yield (condition A)).

^{1}H-NMR (400 MHz, CDCl$_3$):δ 8.00 (s, 1H), 7.41-7.22 (m, 7H), 7.16-7.04 (m, 2H), 6.63 (s, 1H), 5.35 (s, 2H).

^{13}C-NMR (100 MHz, CDCl$_3$):δ 137.9, 136.5, 130.7, 129.1, 128.6, 128.2, 126.9, 126.7, 124.8, 120.8, 110.7, 102.9, 102.9, 50.6.

HRMS (FAB) (*m*/*z*): [M+H]$^+$ calced. for $C_{16}H_{13}N_2$, 233.1079; found, 233.1076.

1H-indole-5-carbonitrile (3y)[S16]

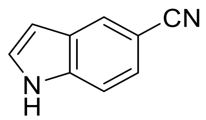

Isolated as pale-brown powder (31.2 mg, 55% yield (condition A))

^{1}H-NMR (400 MHz, CDCl$_3$):δ 8.52 (br s, 1H), 8.02-7.98 (m, 1H), 7.46 (app dt, *J* = 8.5 Hz, 0.8 Hz, 1H), 7.43 (dd, *J* = 8.5 Hz, 1.5 Hz), 7.34 (dd, *J* = 3.1 Hz, 2.6 Hz, 1H), 6.64 (ddd, *J* = 3.1 Hz, 2.1 Hz, 0.7 Hz, 1H).

^{13}C-NMR (100 MHz, CDCl$_3$):δ 137.6, 127.8, 126.6, 126.5 125.1, 120.9, 112.1, 103.7, 103.2.

HRMS (EI) (*m*/*z*): [M]$^+$ calced. for $C_9H_6N_2$, 142.0531; found, 142.0529.

1-methyl-1H-indole-6-carbonitrile (3z)[S20]

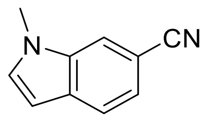

Isolated as pale-brown powder (63.0 mg, 99% yield (condition A)).

^{1}H-NMR (400 MHz, CDCl$_3$):δ 7.67 (d, *J* = 8.5 Hz, 1H), 7.66 (s, 1H), 7.33 (d, *J* = 8.5 Hz, 1H), 7.26 (s, 1H), 6.57 (s, 1H), 3.84 (s, 3H).

^{13}C-NMR (100 MHz, CDCl$_3$):δ 135.6, 132.7, 131.7, 122.3, 121.7, 120.9, 114.3, 104.1, 102.1, 33.2.

HRMS (FAB) (*m*/*z*): [M+H]$^+$ calced. for $C_{10}H_9N_2$, 157.0766; found, 157.0761.

9-methyl-9H-carbazole-3-carbonitrile (3aa)[S21]

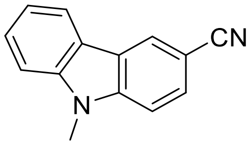

Isolated as yellow powder (70.7 mg, 86 % yield (condition A)).

^{1}H-NMR (400 MHz, CDCl$_3$):δ 8.39 (s, 1H), 8.11 (d, *J* = 7.8 Hz, 1H), 7.71 (dd, *J* = 8.5 Hz, 1.1 Hz, 1H), 7.57 (app t, *J* = 7.7 Hz, 1H), 7.45 (app t, *J* = 8.6 Hz, 2H), 7.34 (app t, *J* = 7.6 Hz, 1H), 3.89 (s, 3H).

^{13}C-NMR (100 MHz, CDCl$_3$):δ 142.5, 141.5, 128.9, 127.2, 125.0, 122.9, 121.8, 120. 7, 120.6, 120.4, 109.1, 109.1, 101.5, 29.3.

HRMS (FAB) (*m*/*z*): [M+H]$^+$ calced. for $C_{14}H_{11}N_2$, 207.0922; found, 207.0921.

1-methylindoline-5-carbonitrile (3ab)[S22]

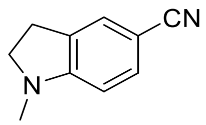

Isolated as white powder (44.3 mg, 70% yield (condition A)).

^{1}H-NMR (400 MHz, CDCl$_3$):δ 7.35 (d, *J* = 7.9 Hz, 1H), 7.22 (s, 1H), 6.33 (d, *J* = 7.9 Hz, 1H), 3.49 (t, *J* = 8.4 Hz, 2H), 2.99 (d, *J* = 8.4 Hz, 2H), 2.83 (s, 3H).

^{13}C-NMR (100 MHz, CDCl$_3$):δ 156.2, 133.6, 130.6, 127.4, 121.1, 105.6, 98.5, 55.0, 34.4, 27.9.

HRMS (EI) (*m*/*z*): [M]$^+$ calced. for $C_{10}H_{10}N_2$, 158.0844; found, 158.0840.

dibenzo[*b*,*d*]furan-2-carbonitrile (3ac)[S23]

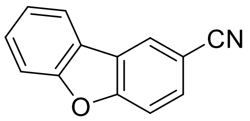

Isolated as white powder (68.0 mg, 88% yield (condition A)).

^{1}H-NMR (400 MHz, CDCl$_3$):δ 8.27 (d, *J* = 1.5 Hz, 1H), 7.98 (d, *J* = 7.8 Hz, 1H), 7.74 (dd, *J* = 8.5 Hz, *J* = 1.5 Hz, 1H), 7.67-7.60 (m, 2H), 7.56 (dd, *J* = 8.3 Hz, *J* = 7.2 Hz, 1H), 7.42 (dd, *J* = 7.8 Hz, 7.2 Hz, 1H).

^{13}C-NMR (100 MHz, CDCl$_3$):δ 158.1, 157.0, 131.0, 128.9, 125.6, 125.4, 123.0, 122.7, 121.2, 119.3, 113.0, 112.2, 106.8.

HRMS (EI) (*m*/*z*): [M]$^+$ calced. for $C_{13}H_7NO$, 193.0528; found, 193.0527.

benzo[*b*]thiophene-5-carbonitrile (3ad)[S24]

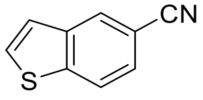

Isolated as white powder (30.7 mg, 48% yield (condition A)).

^{1}H-NMR (400 MHz, CDCl$_3$):δ 8.15 (app s, 1H), 7.97 (d, *J* = 8.3 Hz, 1H), 7.61 (d, *J* = 5.5 Hz, 1H), 7.56 (dd, *J* = 8.3 Hz, *J* = 1.0 Hz ,1H), 7.41 (d, *J* = 5.5 Hz, 1H).

^{13}C-NMR (100 MHz, CDCl$_3$):δ 144.0, 139.5, 129.2, 128.3, 126.3, 123.9, 123.6, 119.5, 108.2.

HRMS (EI) (*m*/*z*): [M]$^+$ calced. for $C_{13}H_7NO$, 159.0143; found, 159.0139.

2-methylbenzo[*d*]oxazole-5-carbonitrile (3ae)[S25]

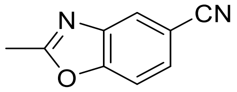

Isolated as white powder (38.9 mg, 61% yield (condition A)).

^{1}H-NMR (400 MHz, CDCl$_3$):δ 7.97 (s, 1H), 7.62-7.54 (m, 2H), 2.69 (s, 3H).

^{13}C-NMR (100 MHz, CDCl$_3$):δ 166.3, 153.5, 142.2, 128.9, 124.2, 118.9, 111.6, 108.3, 14.7.

HRMS (EI) (*m*/*z*): [M]$^+$ calced. for $C_9H_6N_2O$, 158.0480; found, 158.0482.

1-benzyl-1H-pyrazole-4-carbonitrile (3af)[S26]

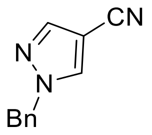

Isolated as white powder (38.9 mg, 61% yield (condition A)).

^{1}H-NMR (400 MHz, $CDCl_3$):δ 7.82 (s, 1H), 7.74 (s, 1H), 7.42-7.32 (m, 3H), 7.27-7.22 (m, 2H), 5.33 (s, 2H).

^{13}C-NMR (100 MHz, $CDCl_3$):δ 142.6, 134.5, 134.3, 129.3, 129.0, 128.3, 113.4, 92.9, 52.7.

HRMS (EI) (*m*/*z*): [M]$^+$ calced. for $C_9H_6N_2O$, 158.0480; found, 158.0482.

2-(piperidin-1-yl)pyrimidine-5-carbonitrile (3ag)[S26]

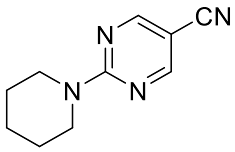

Isolated as white powder (47.0 mg, 62% yield (condition A)).

^{1}H-NMR (400 MHz, $CDCl_3$):δ 8.46 (s, 2H), 3.86 (t, J = 5.6 Hz, 4H), 1.76-1.68 (m, 2H), 1.66-1.58 (m, 4H).

^{13}C-NMR (100 MHz, $CDCl_3$):δ 160.8, 160.4, 117.2, 94.8, 45.2, 25.9, 24.7.

HRMS(EI) (*m*/*z*): [M]$^+$ calced. for $C_{10}H_{12}N_4$, 188.1062; found, 188.1056.

9. X-Ray Crystallographic Analysis

The crystals were mounted on the CryoLoop (Hampton Research Corp.) with a layer of light mineral oil and placed in a nitrogen stream at 113(1) K. All measurements were made on a Rigaku XtaLAB P200 system with graphite-monochromated Mo-Kα (0.71075 Å) radiation. Crystal data and structure refinement parameters were listed below (Table S5). The structure was solved by SHELXT-2014[S27] and refined on F^2 by full matrix least-squares method, using SHELXL-2016.[S28] Non-hydrogen atoms were anisotropically refined. H-atoms were included in the refinement on calculated positions riding on their carrier atoms. The function minimized was $[\Sigma w(Fo^2 - Fc^2)^2]$ ($w = 1 / [\sigma^2(Fo^2) + (aP)^2 + bP]$), where $P = (\mathrm{Max}(Fo^2,0) + 2Fc^2) / 3$ with $\sigma^2(Fo^2)$ from counting statistics. The function $R1$ and $wR2$ were $(\Sigma||Fo| - |Fc||) / \Sigma|Fo|$ and $[\Sigma w(Fo^2 - Fc^2)^2 / \Sigma(w(Fo^2)^2)]^{1/2}$, respectively. The ORTEP-3 program was used to draw the molecule.[S29]

Table S5. Crystal Data and Data Collection Parameters of Complex **6**

	**6**
empirical formula	$C_{20}H_{17}BrN_2NiO$
formula weight	439.97
crystal system	*monoclinic*
space group	*Cc* (No. 9)
a, Å	11.964(13)
b, Å	20.02(2)
c, Å	7.423(11)
α, deg.	-
β, deg.	90.11(4)
γ, deg.	-
V, Å^3	1778(4)
Z	4
Dcalcd, g/cm^3	1.644
μ[Mo-Kα], mm^{-1}	3.355
T, K	113
crystal size, mm	0.08 × 0.02 × 0.02
θ range for data collection (deg.)	3.384 to 27.738
no. of reflections measured	21939
unique data (Rint)	4057
data / restraints / parameters	4057 / 2 / 228
$R1$ ($I > 2.0\sigma(I)$)	0.0728
$wR2$ ($I > 2.0\sigma(I)$)	0.1620
$R1$ (all data)	0.1249
$wR2$ (all data)	0.1807
GOF on F^2	0.921
Flack Parameter	-0.02(3)
$\Delta\rho$, e Å^{-3}	1.38, -0.97

10. ^{1}H- and ^{13}C-NMR Spectra of Aryl Nitriles

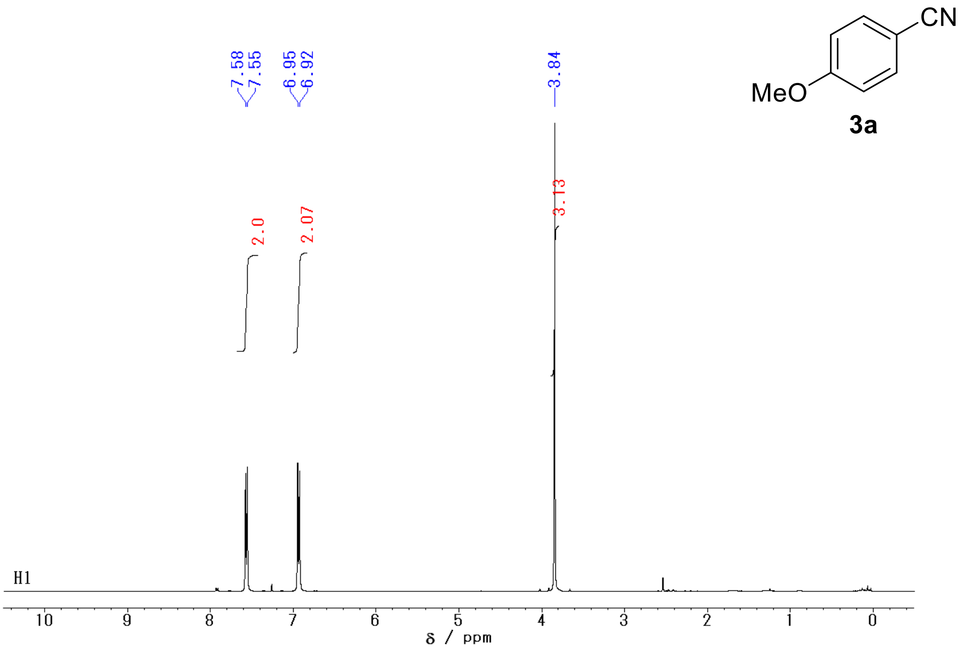

Figure S3. ^{1}H NMR spectrum of **3a**.

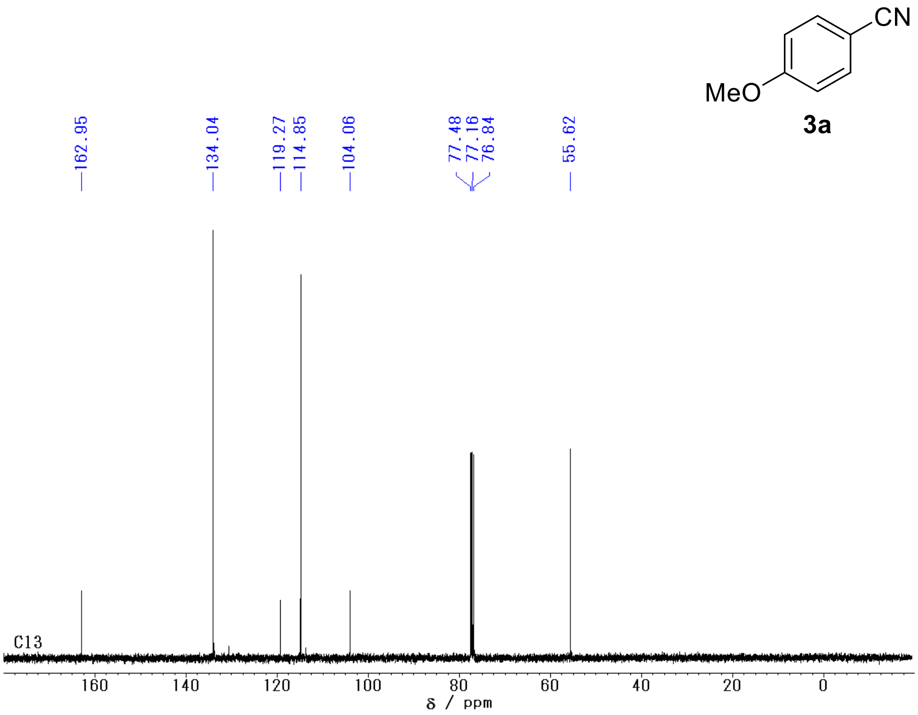

Figure S4. ^{13}C NMR spectrum of **3a**.

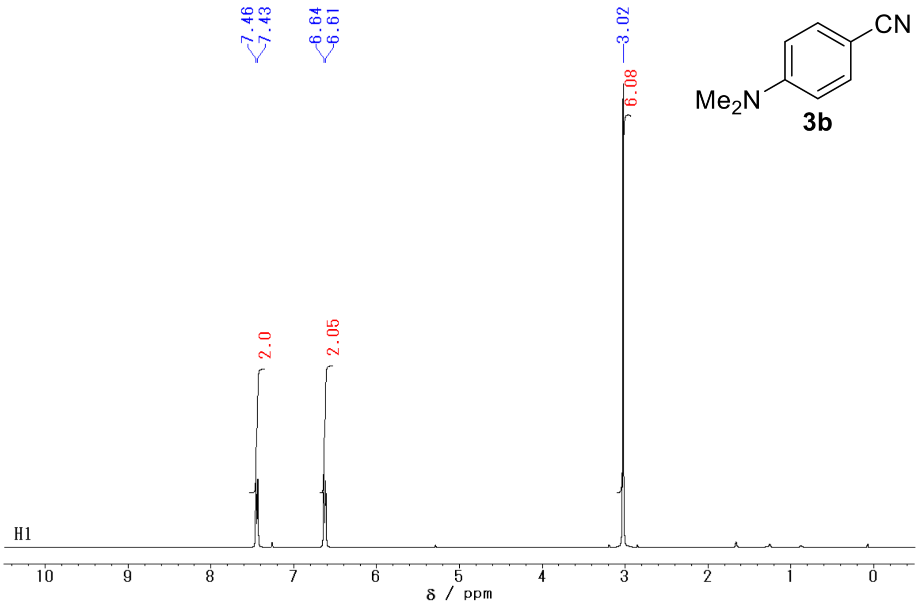

Figure S5. ^{1}H NMR spectrum of **3b**.

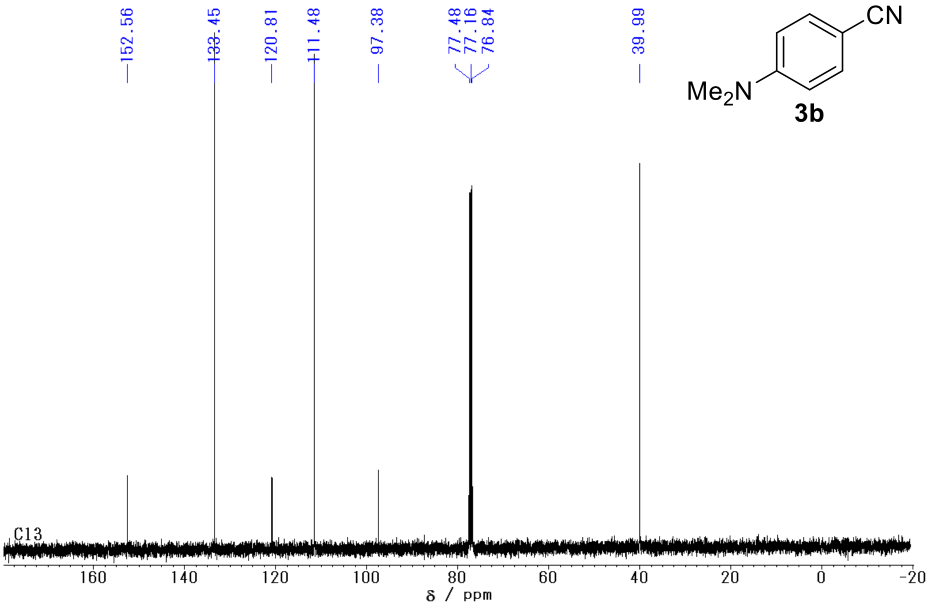

Figure S6. ^{13}C NMR spectrum of **3b**.

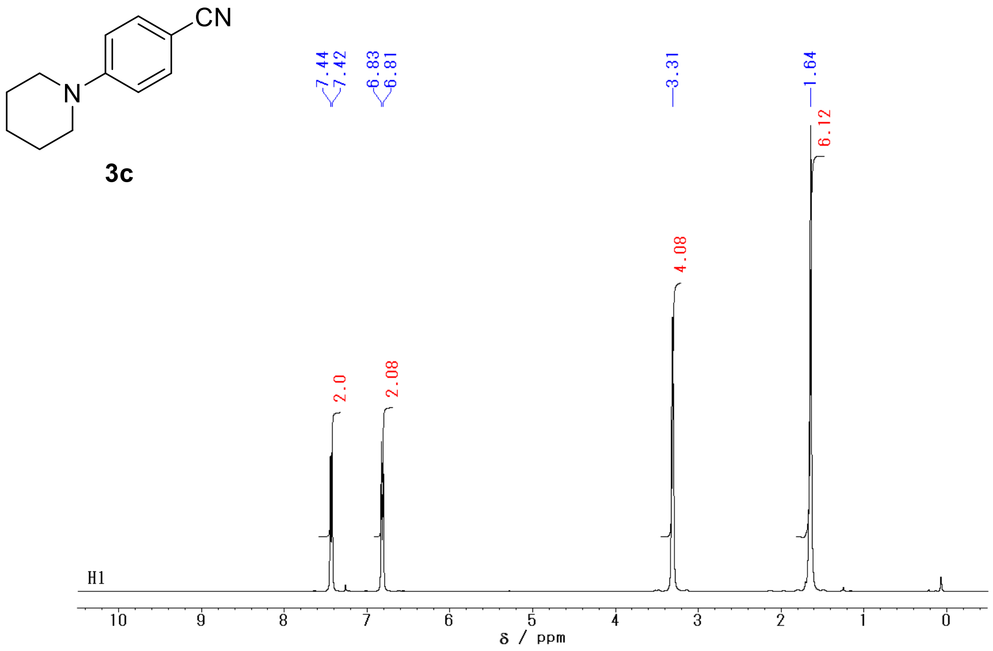

Figure S7. ^{1}H NMR spectrum of **3c**.

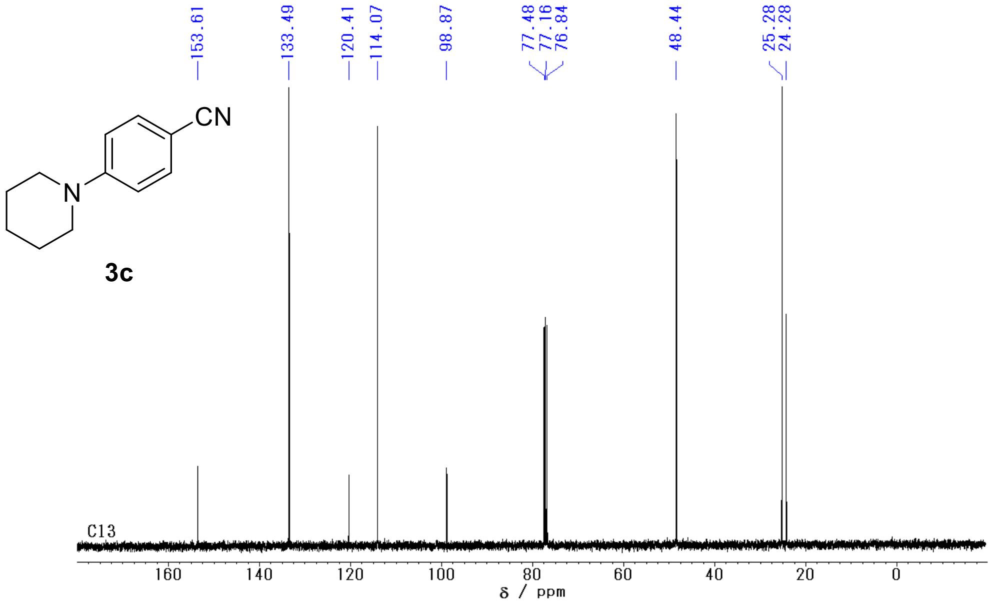

Figure S8. ^{13}C NMR spectrum of **3c**.

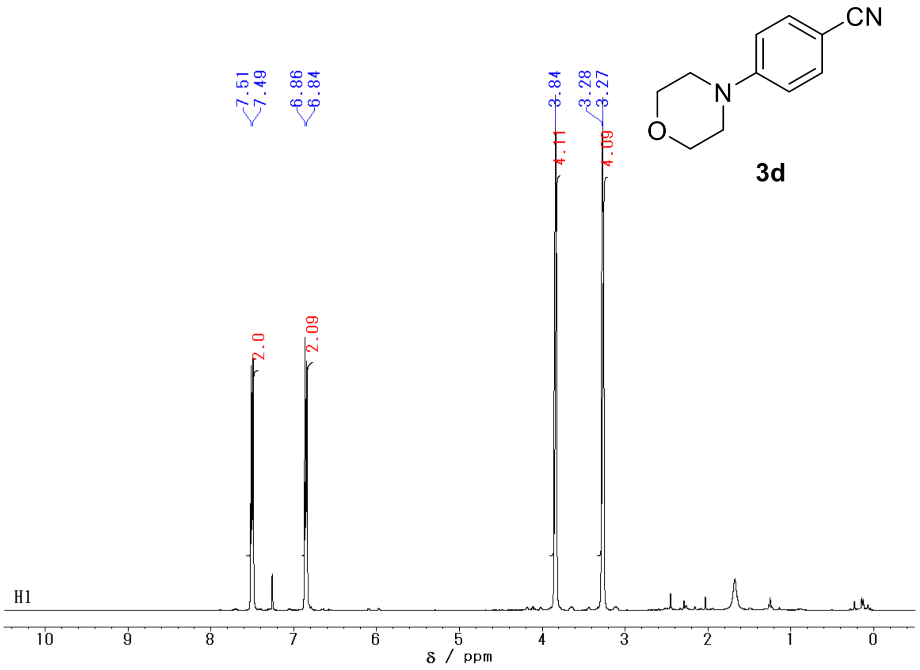

Figure S9. ^{1}H NMR spectrum of **3d**.

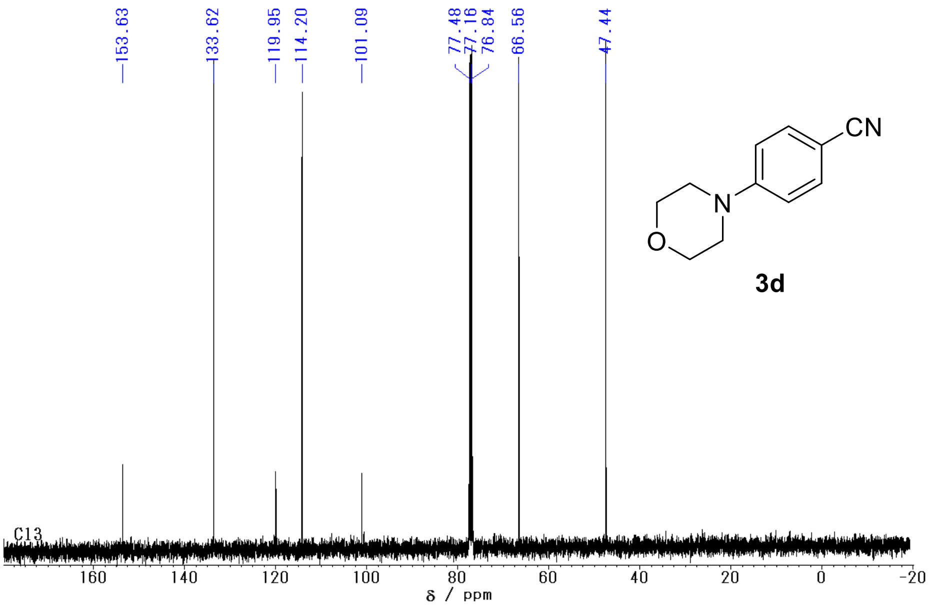

Figure S10. ^{13}C NMR spectrum of **3d**.

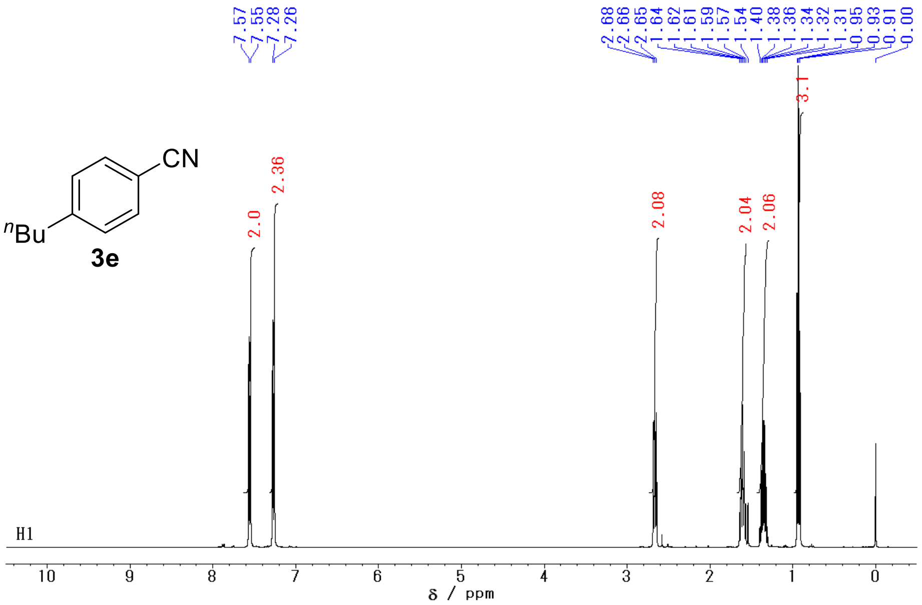

Figure S11. ^{1}H NMR spectrum of **3e**.

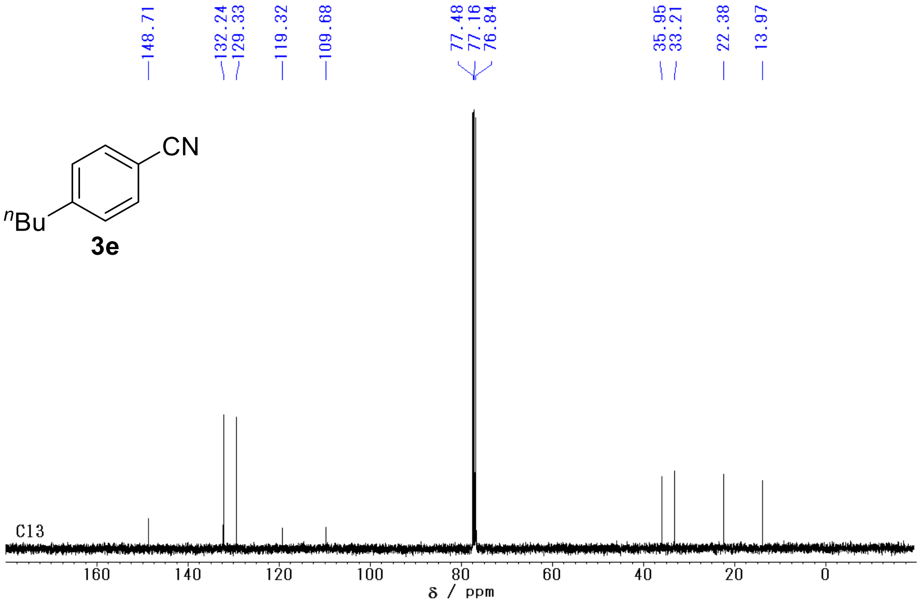

Figure S12. ^{13}C NMR spectrum of **3e**.

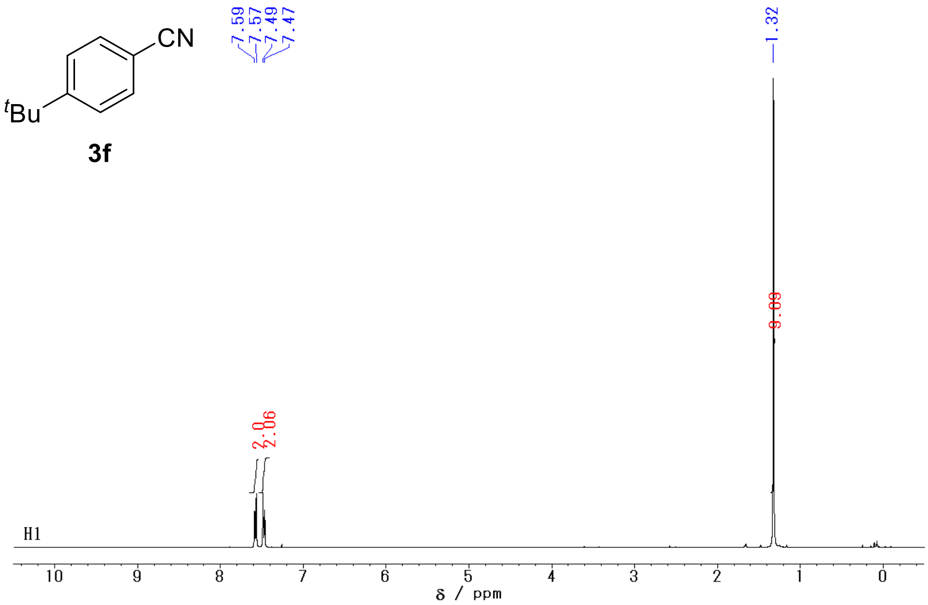

Figure S13. ^{1}H NMR spectrum of **3f**.

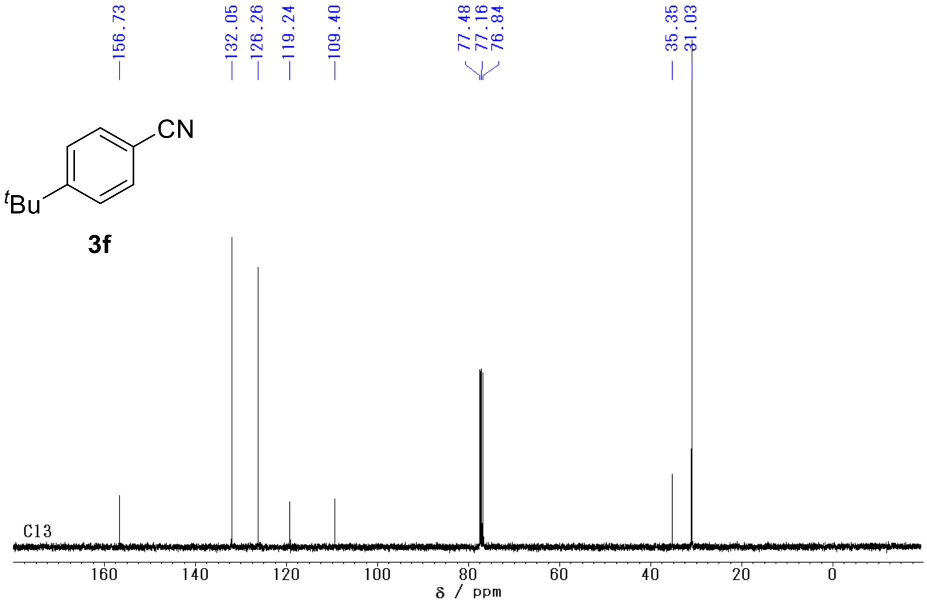

Figure S14. ^{13}C NMR spectrum of **3f**.

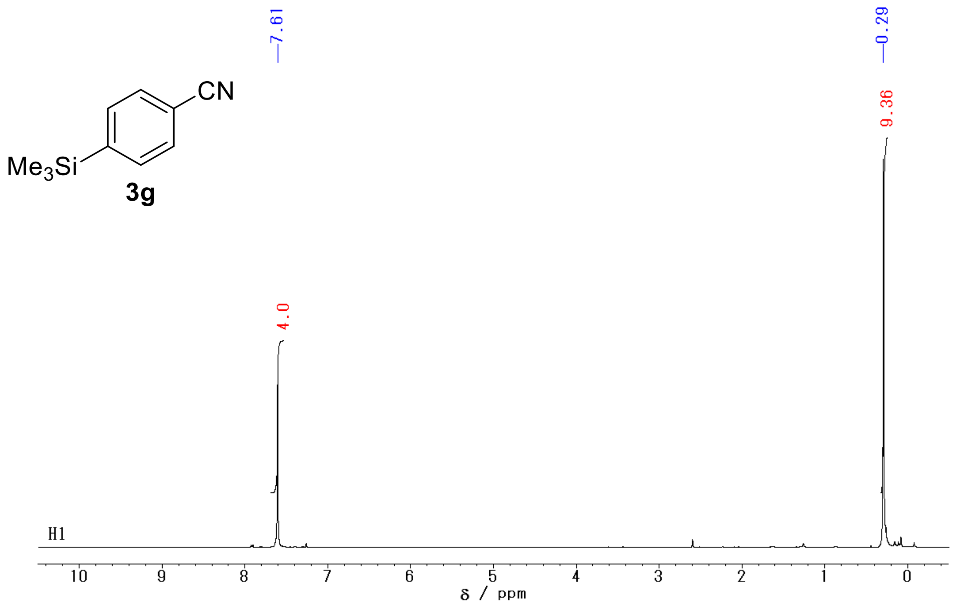

Figure S15. ^{1}H NMR spectrum of **3g**.

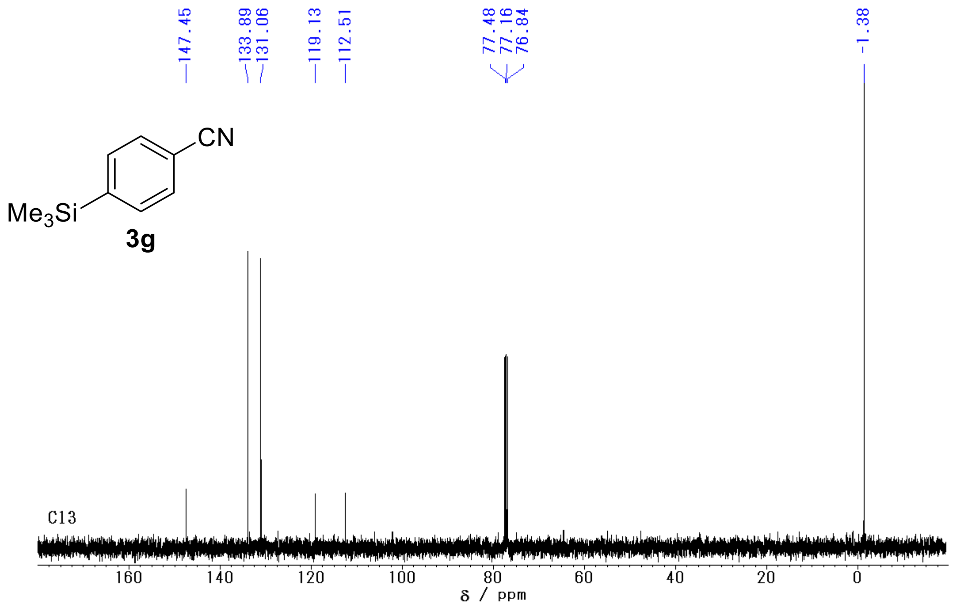

Figure S16. ^{13}C NMR spectrum of **3g**.

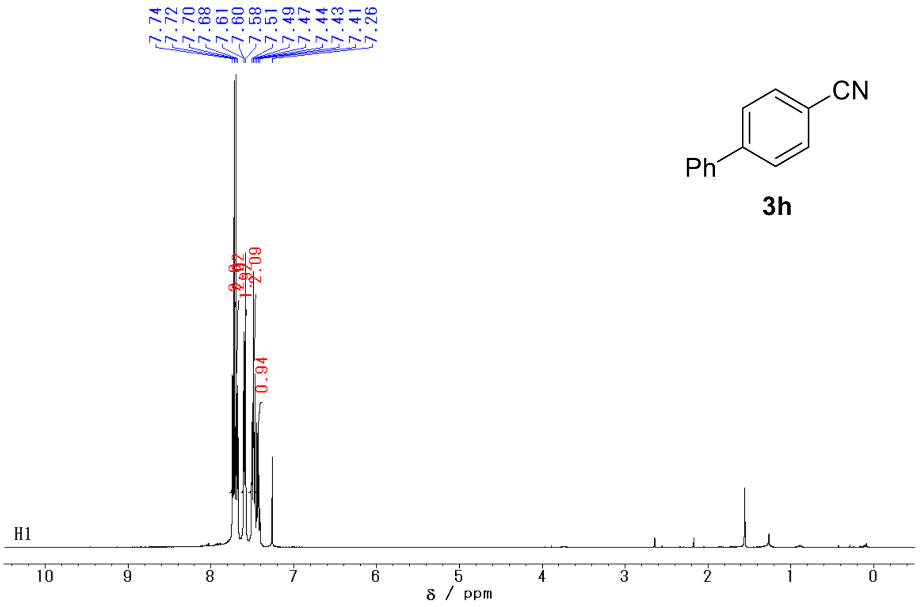

Figure S17. ^{1}H NMR spectrum of **3h**.

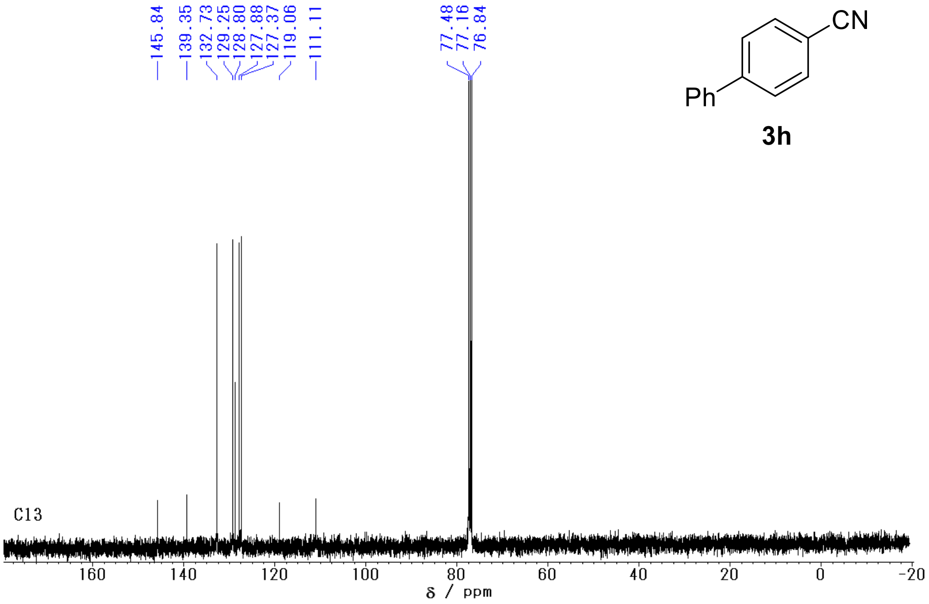

Figure S18. ^{13}C NMR spectrum of **3h**.

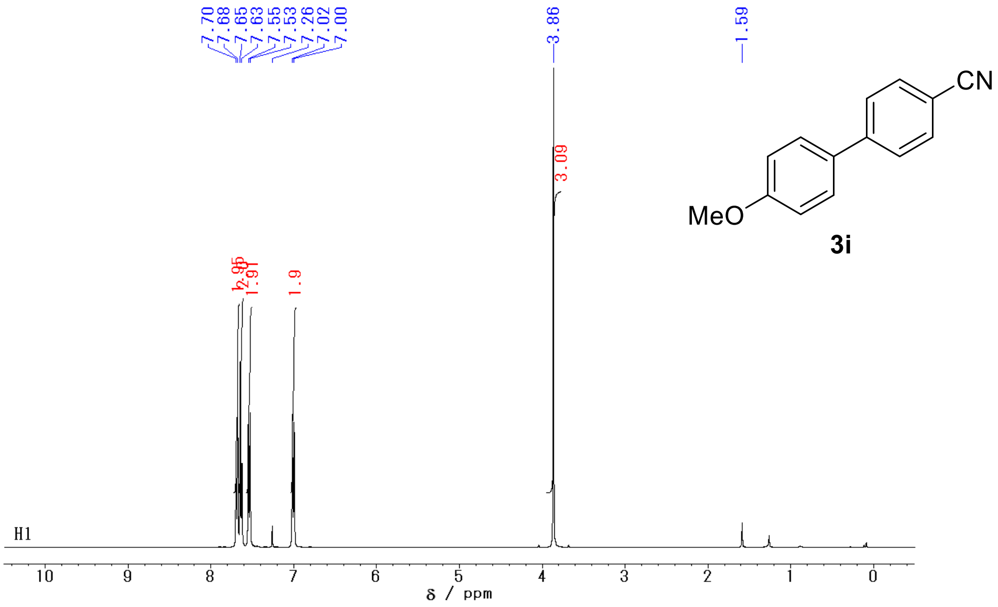

Figure S19. ^{1}H NMR spectrum of **3i**.

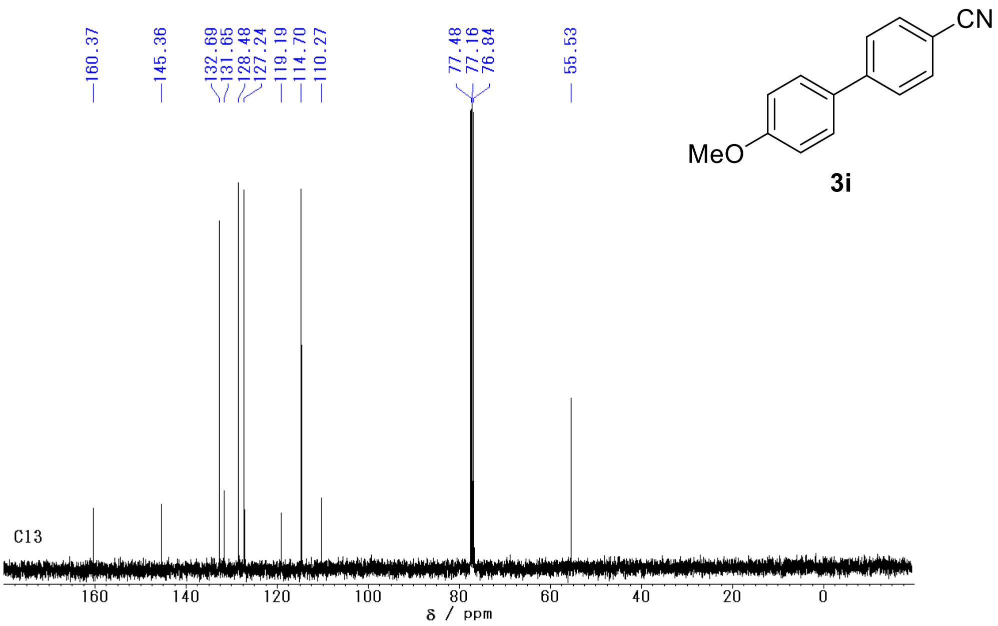

Figure S20. ^{13}C NMR spectrum of **3i**.

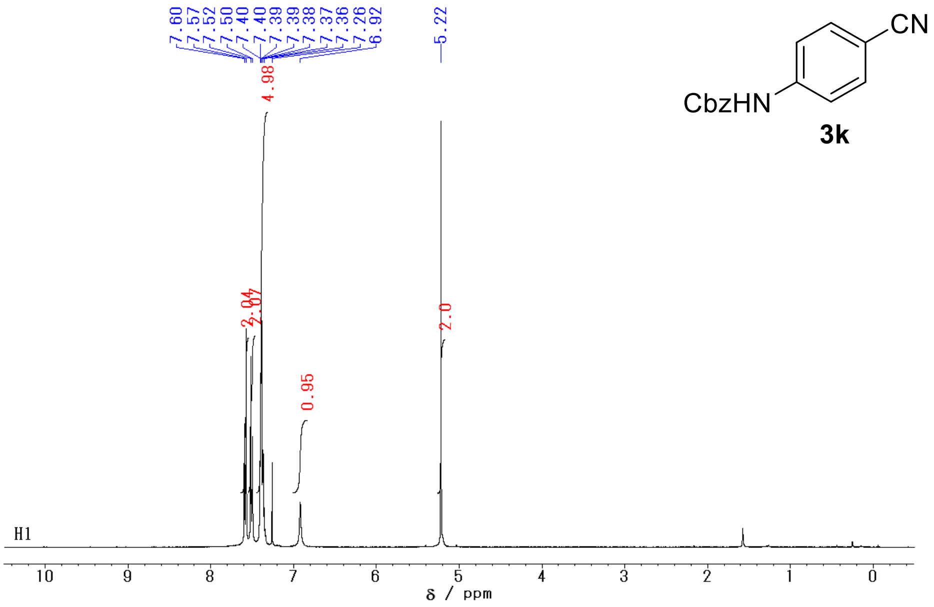

Figure S21. ^{1}H NMR spectrum of **3k**.

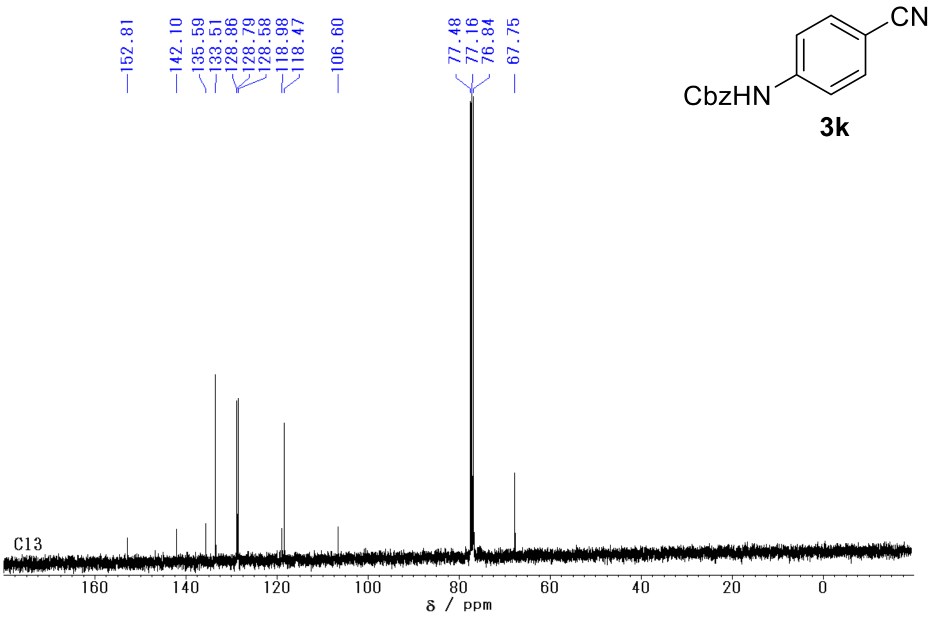

Figure S22. ^{13}C NMR spectrum of **3k**.

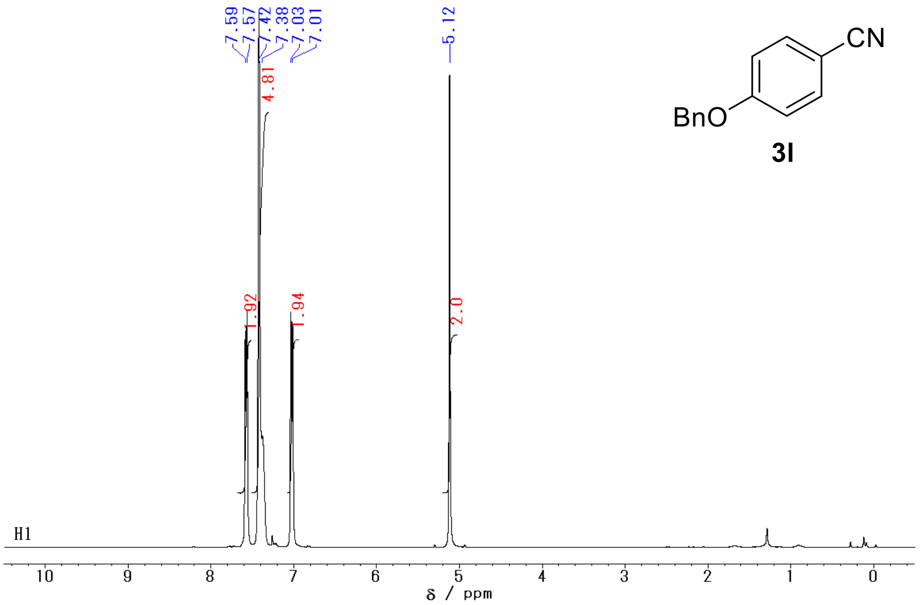

3l

Figure S23. ^{1}H NMR spectrum of **3l**.

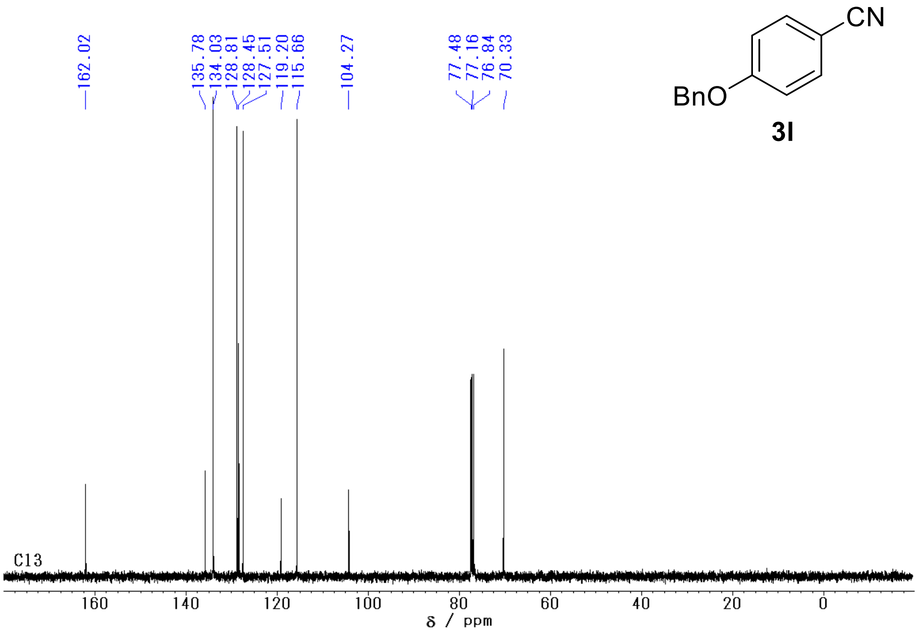

3l

Figure S24. ^{13}C NMR spectrum of **3l**.

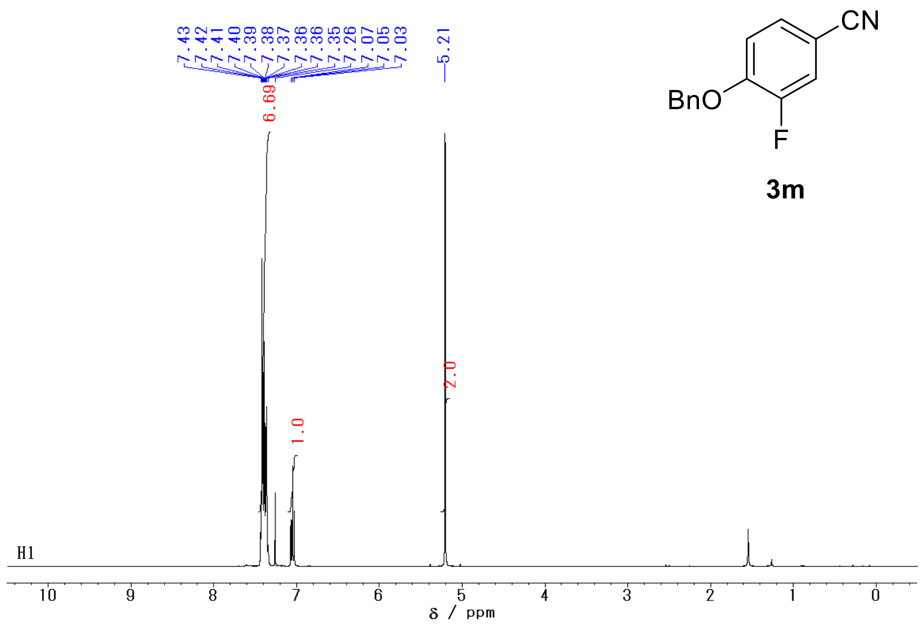

Figure S25. ^{1}H NMR spectrum of **3m**.

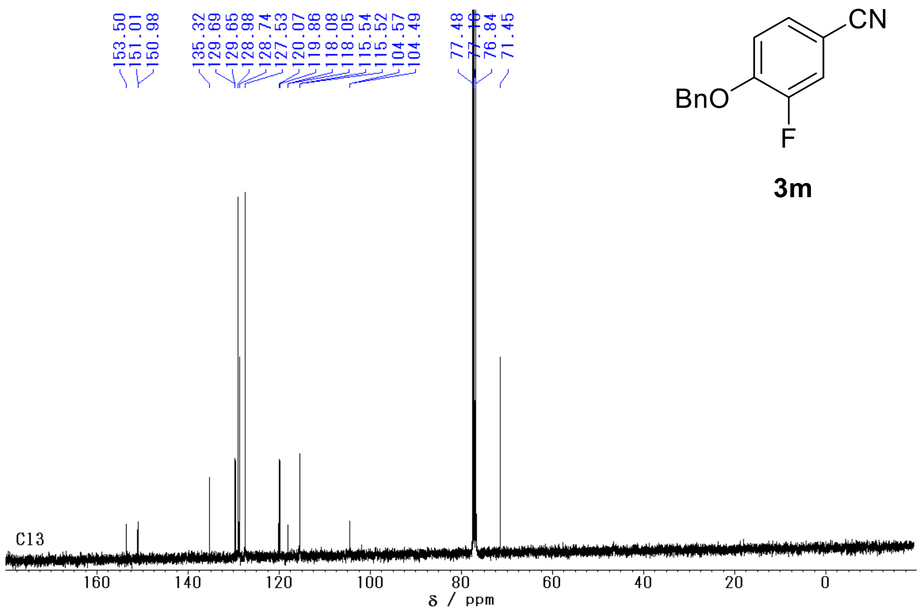

Figure S26. ^{13}C NMR spectrum of **3m**.

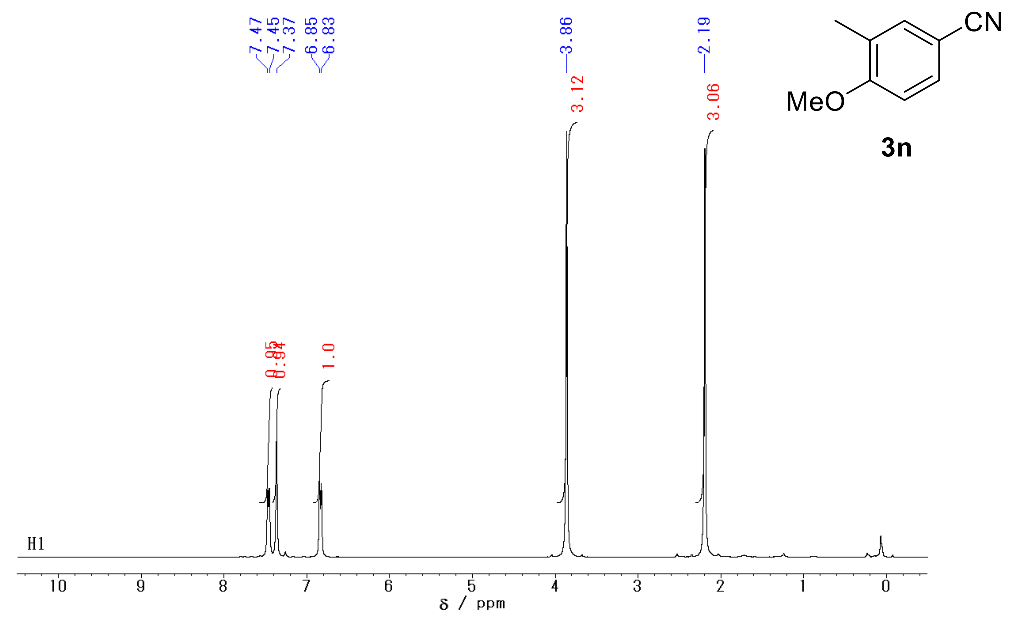

Figure S27. ^{1}H NMR spectrum of **3n**.

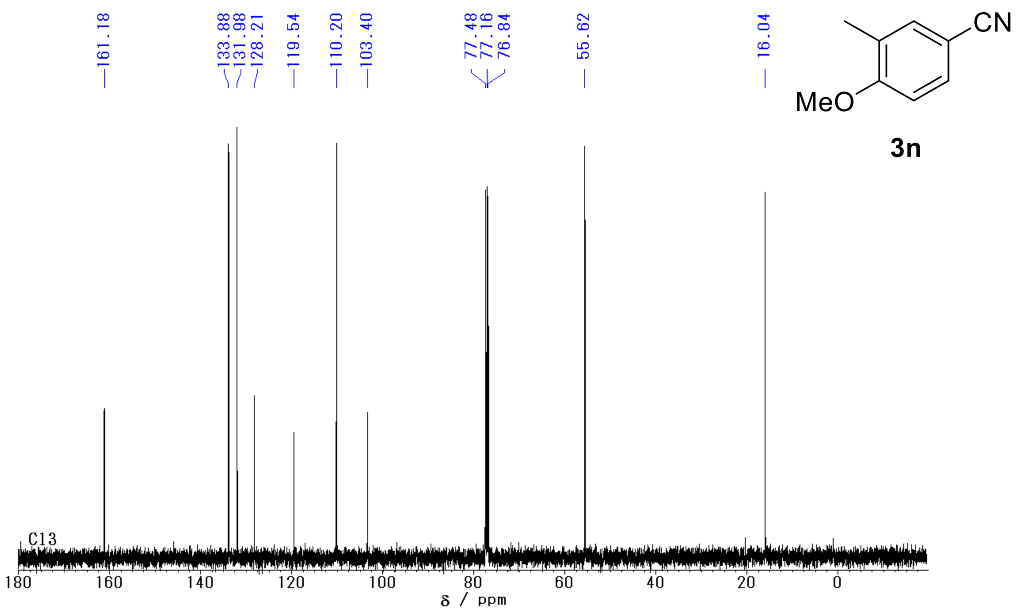

Figure S28. ^{13}C NMR spectrum of **3n**.

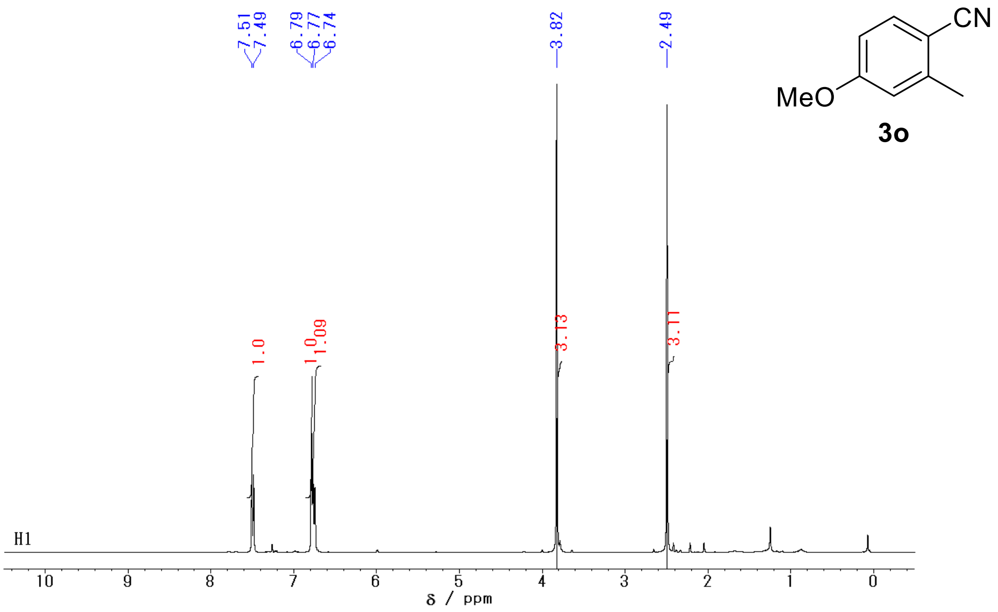

Figure S29. ^{1}H NMR spectrum of **3o**.

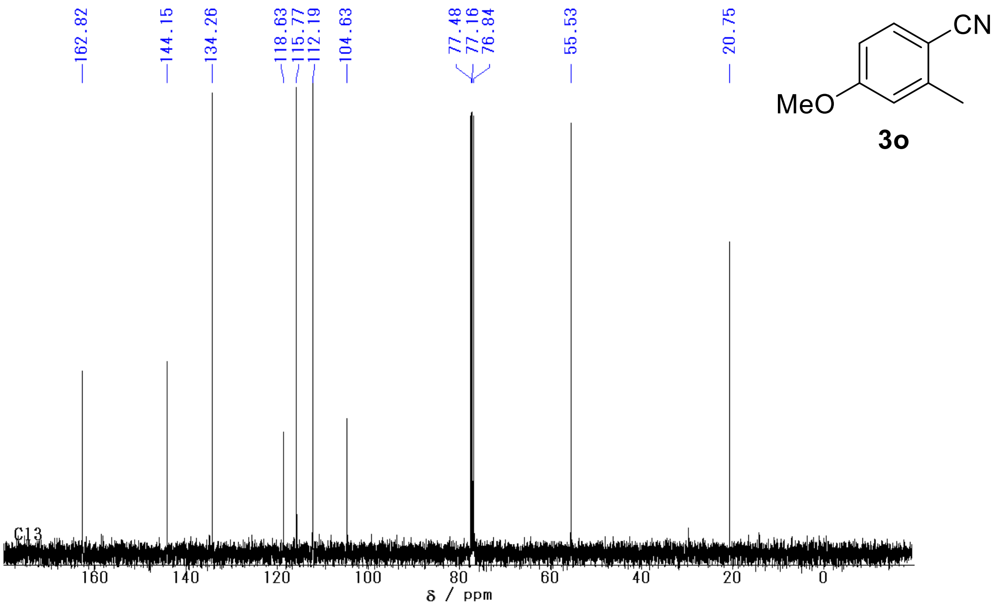

Figure S30. ^{13}C NMR spectrum of **3o**.

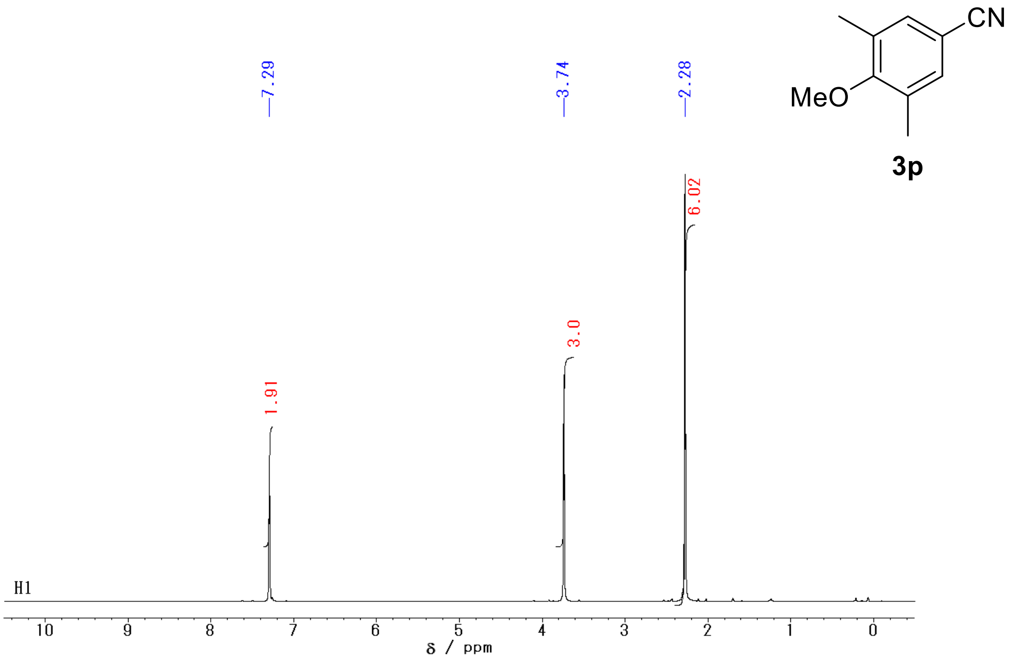

Figure S31. ^{1}H NMR spectrum of **3p**.

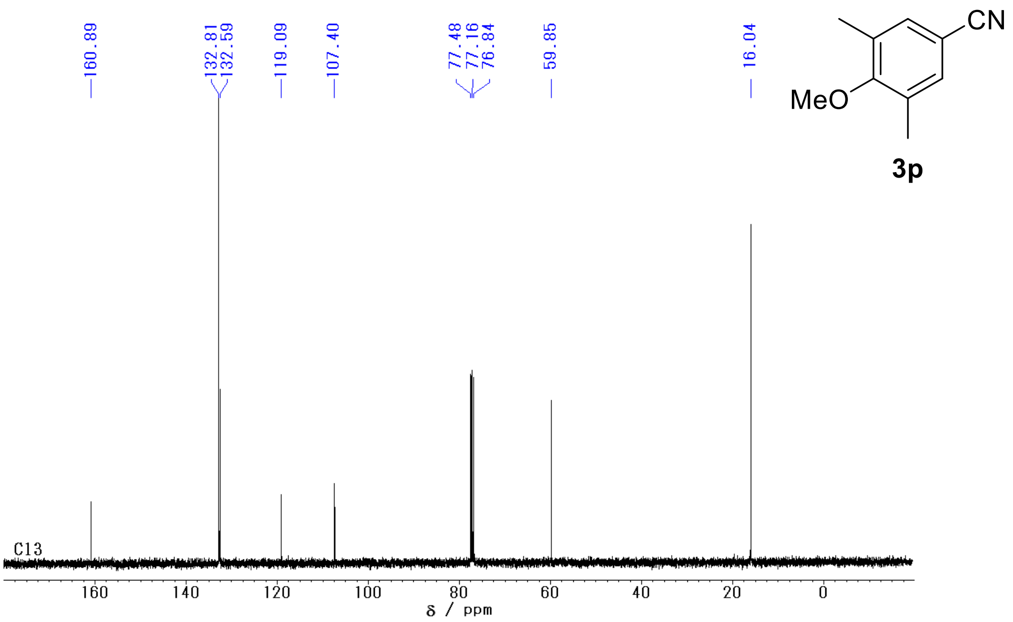

Figure S32. ^{13}C NMR spectrum of **3p**.

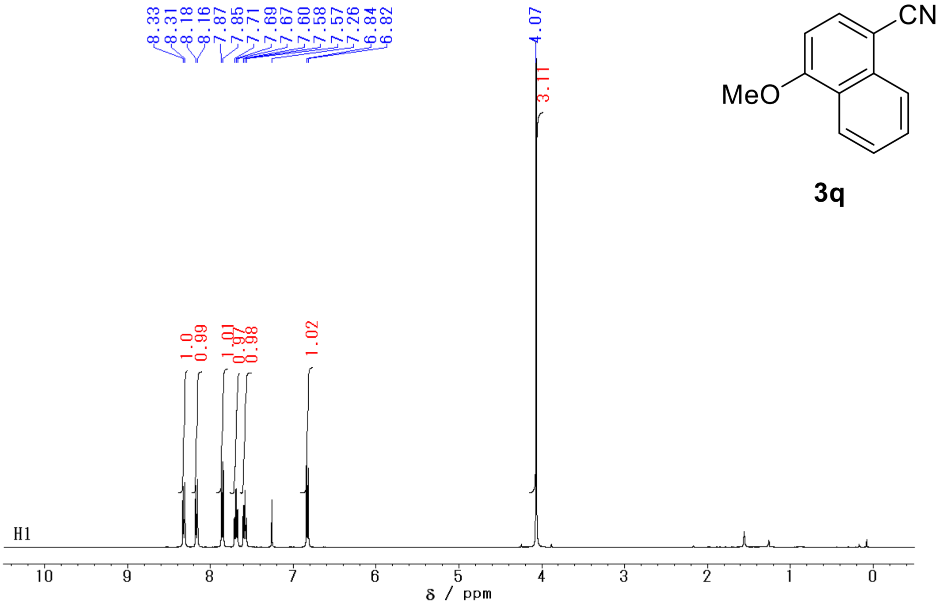

Figure S33. ^{1}H NMR spectrum of **3q**.

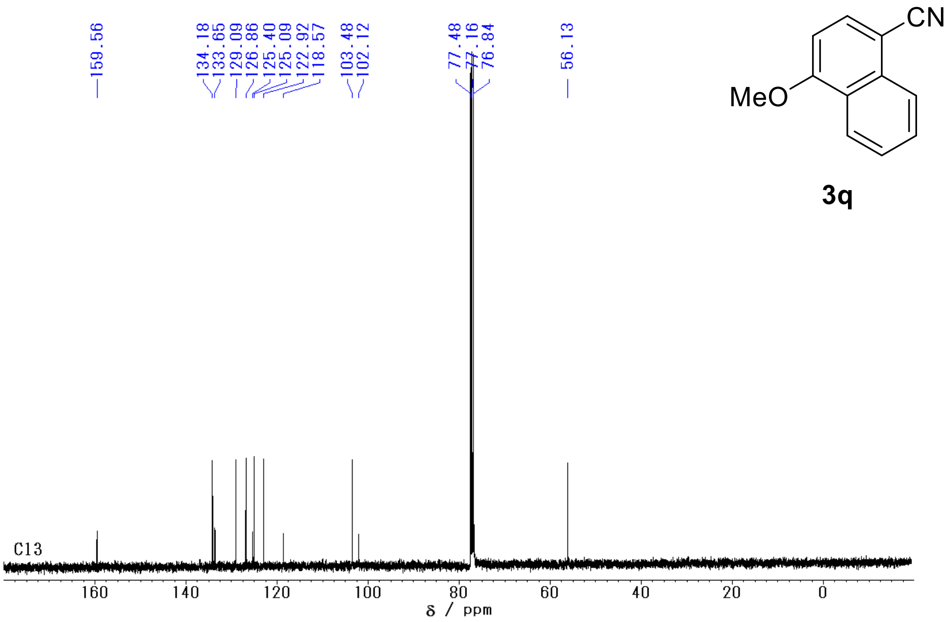

Figure S34. ^{13}C NMR spectrum of **3q**.

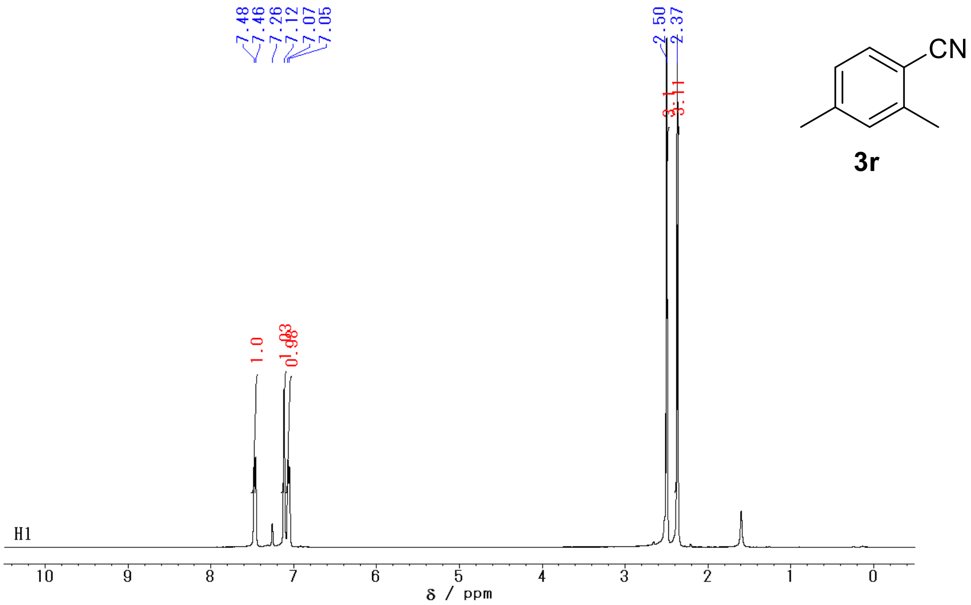

Figure S35. ^{1}H NMR spectrum of **3r**.

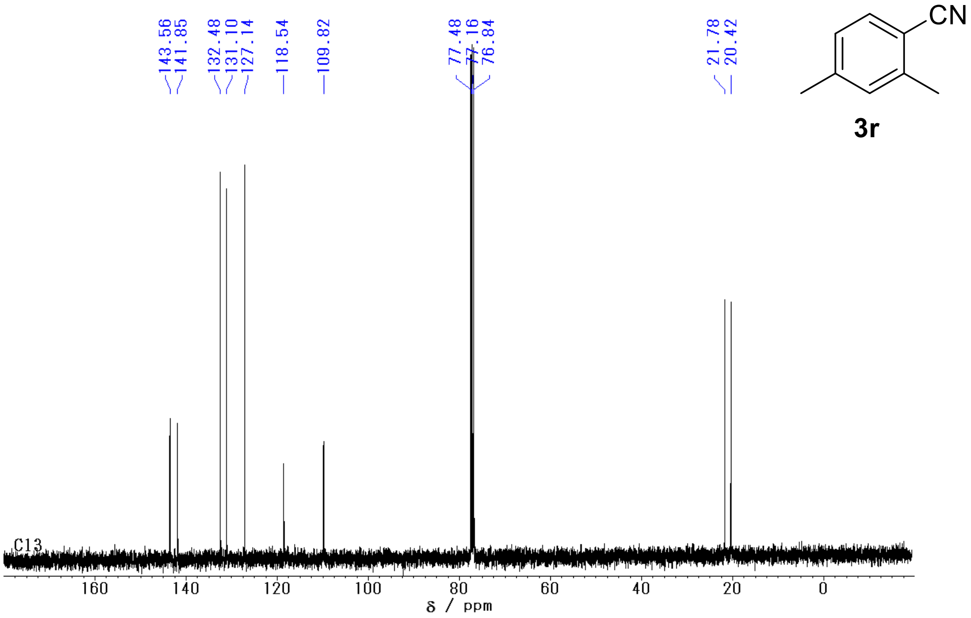

Figure S36. ^{13}C NMR spectrum of **3r**.

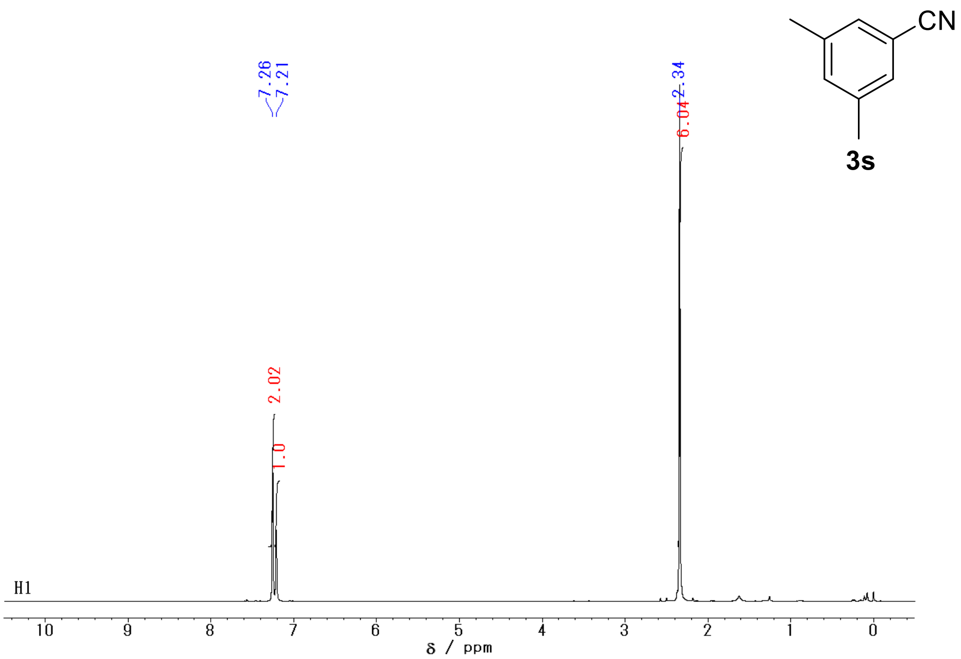

Figure S37. ^{1}H NMR spectrum of **3s**.

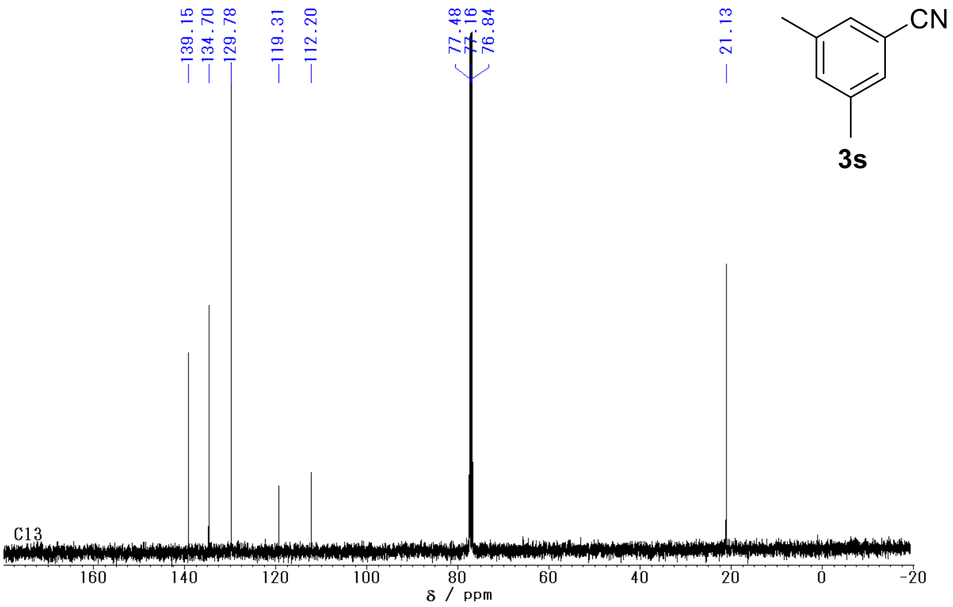

Figure S38. ^{13}C NMR spectrum of **3s**.

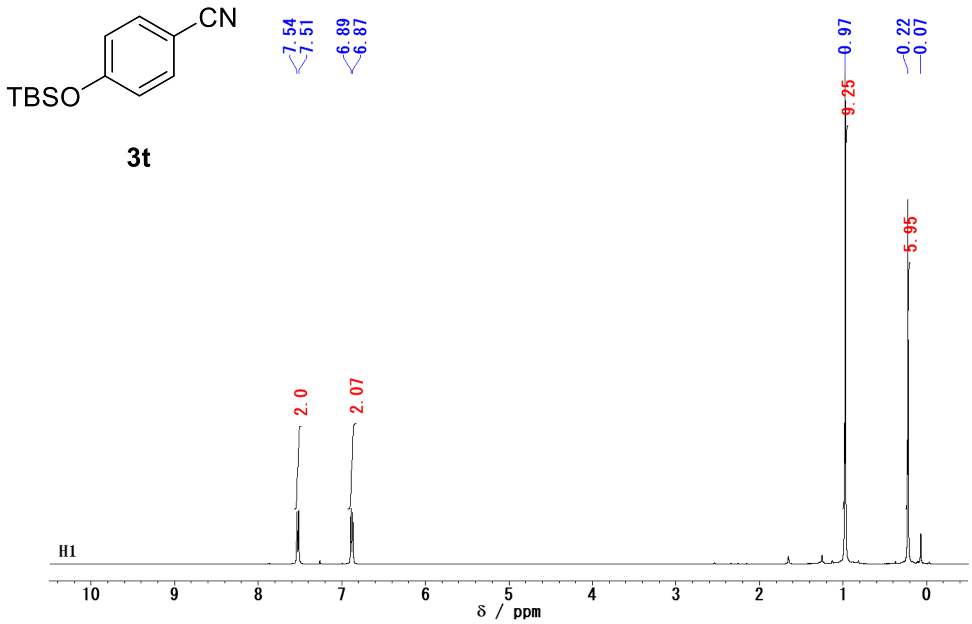

Figure S39. ^{1}H NMR spectrum of **3t**.

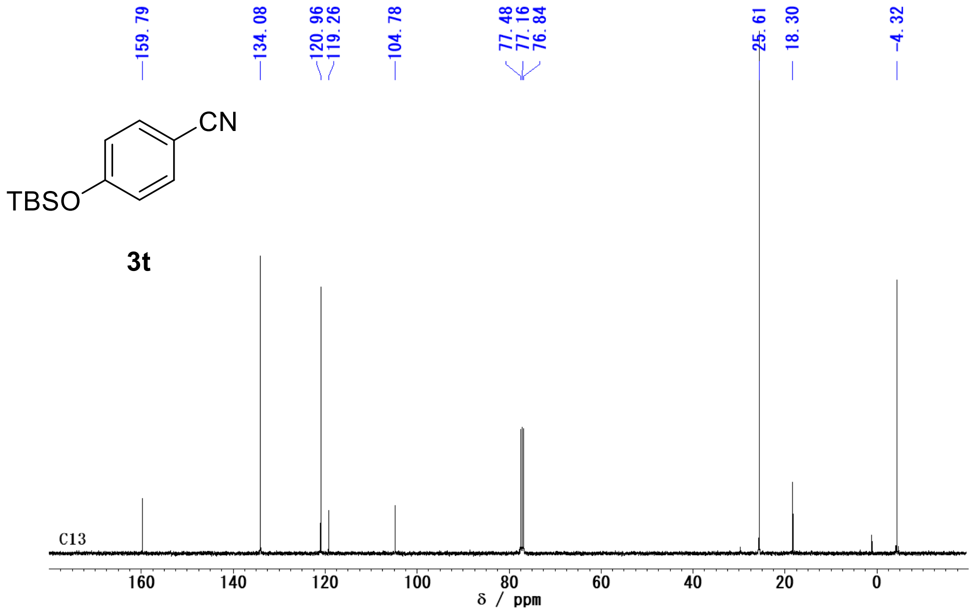

Figure S40. ^{13}C NMR spectrum of **3t**.

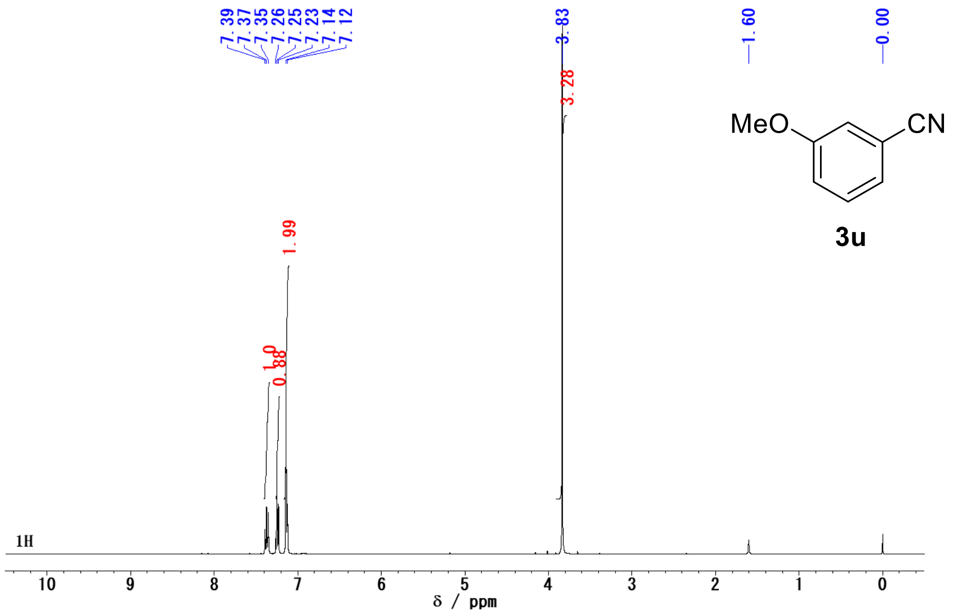

Figure S41. ^{1}H NMR spectrum of **3u**.

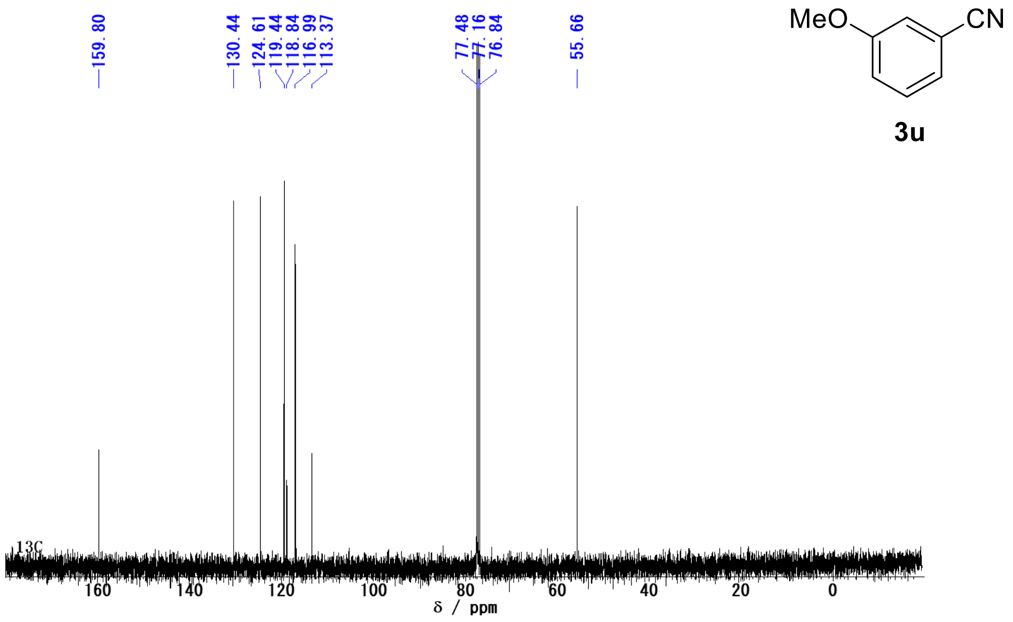

Figure S42. ^{13}C NMR spectrum of **3u**.

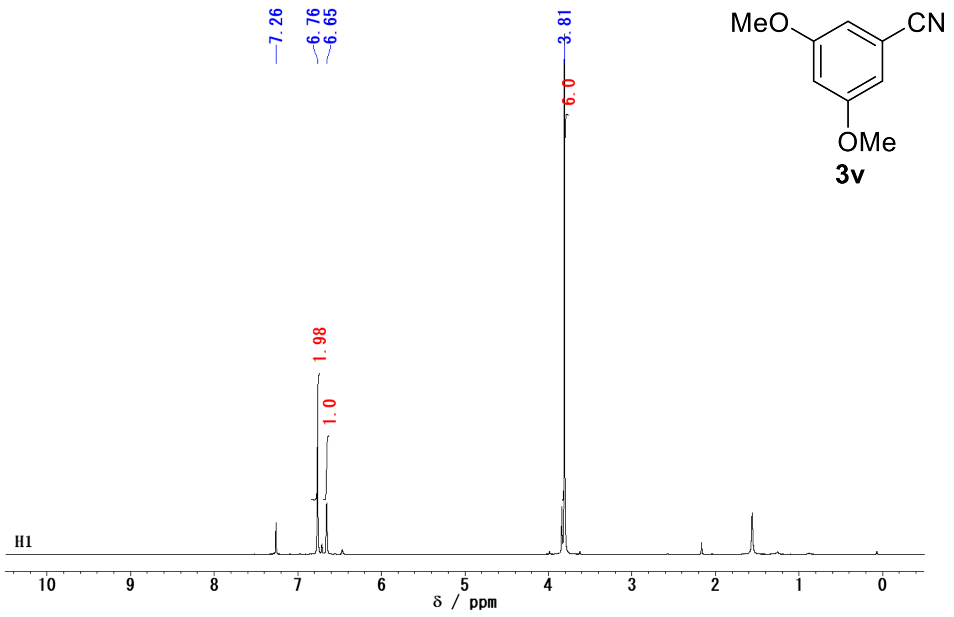

Figure S43. ^{1}H NMR spectrum of **3v**.

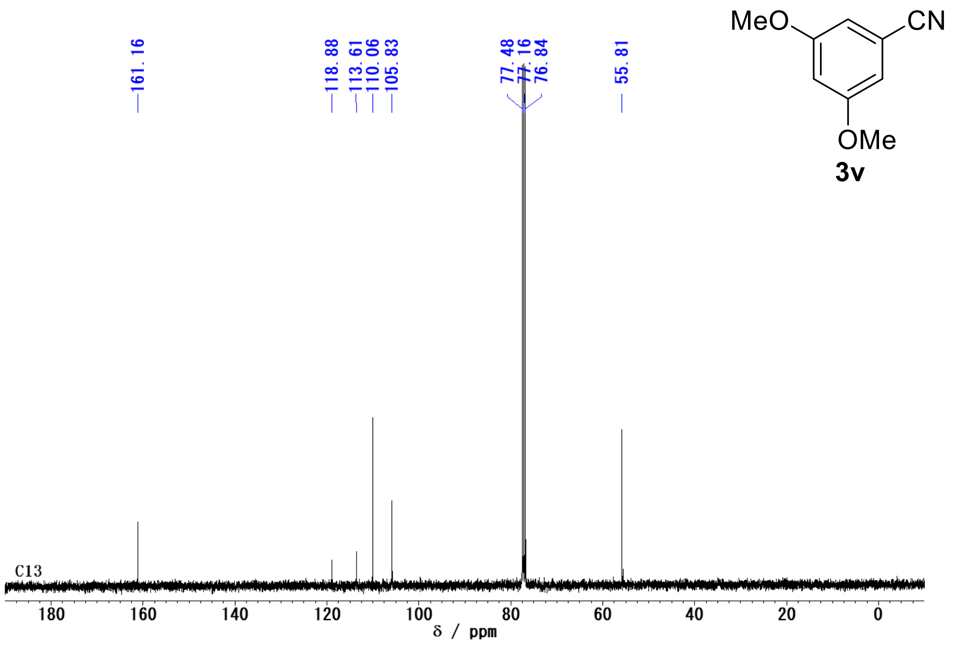

Figure S44. ^{13}C NMR spectrum of **3v**.

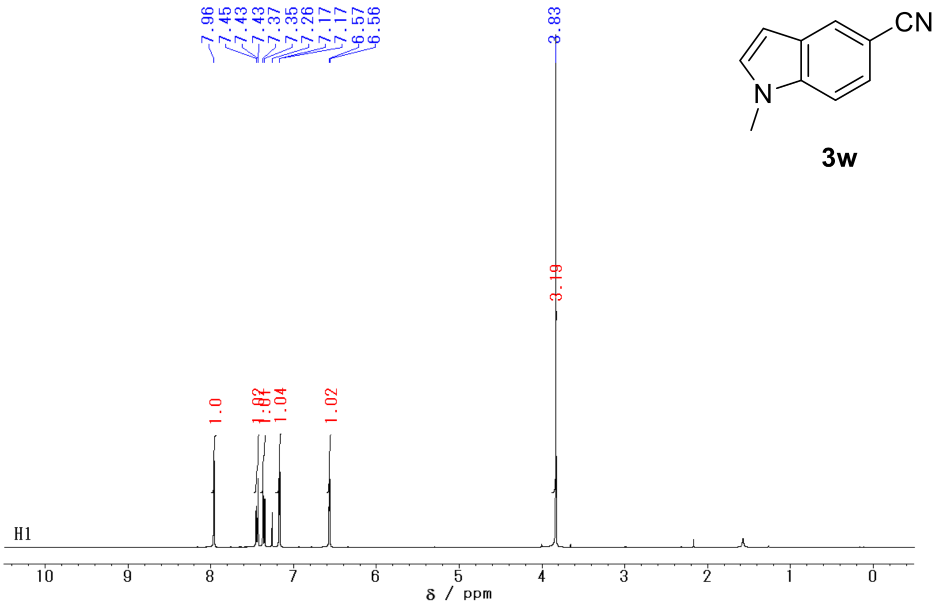

Figure S45. ^{1}H NMR spectrum of **3w**.

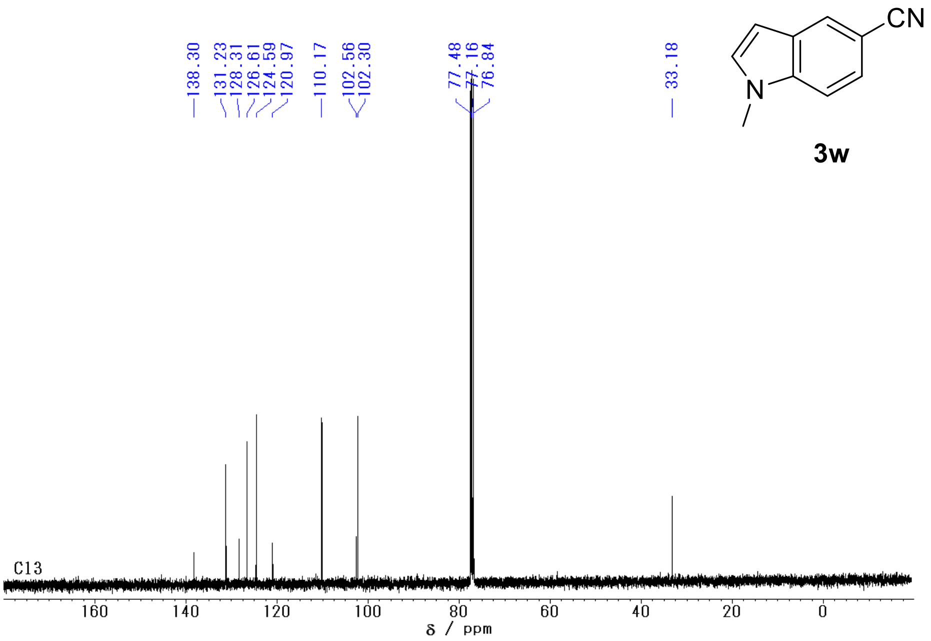

Figure S46. ^{13}C NMR spectrum of **3w**.

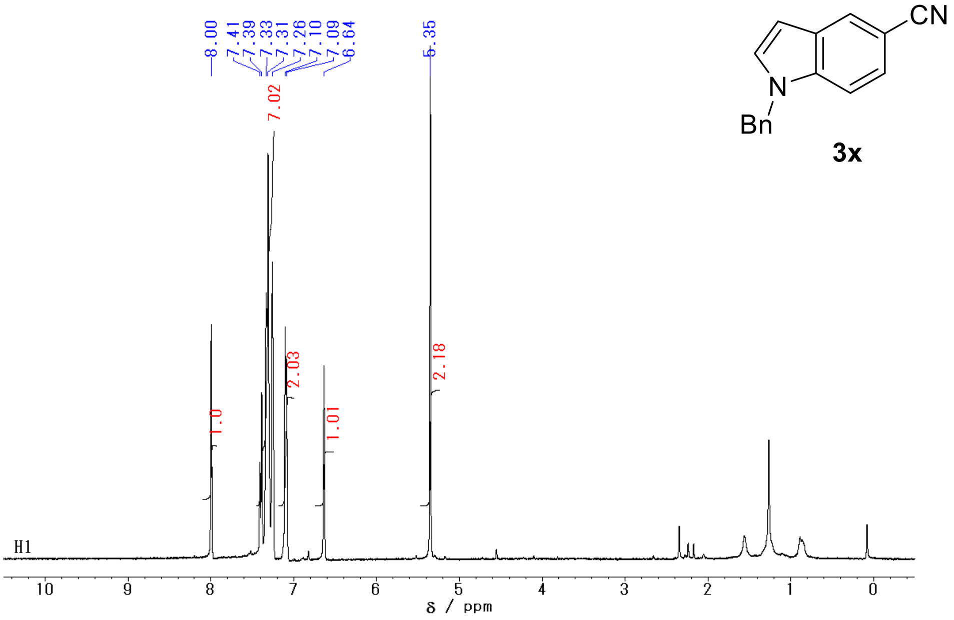

Figure S47. ^{1}H NMR spectrum of **3x**.

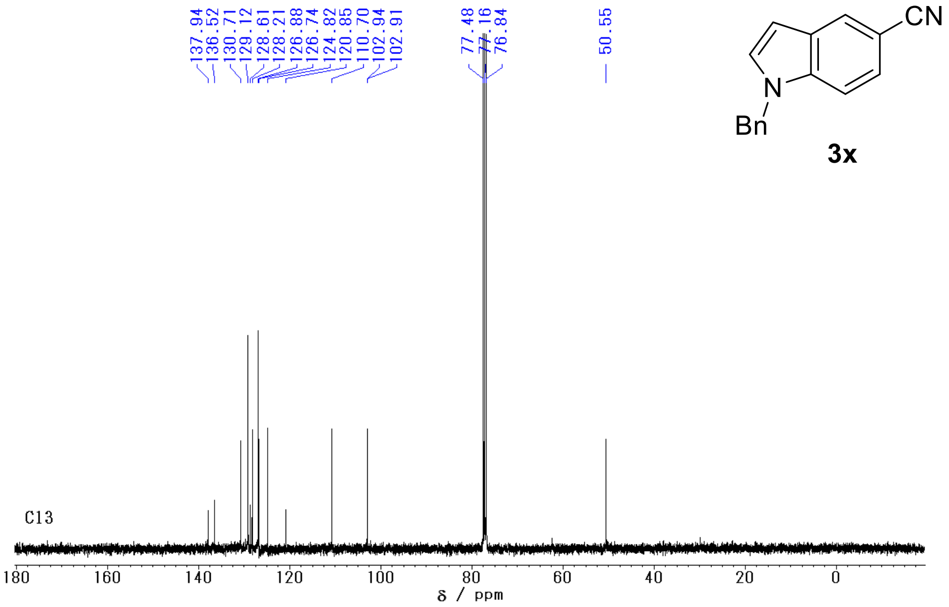

Figure S48. ^{13}C NMR spectrum of **3x**.

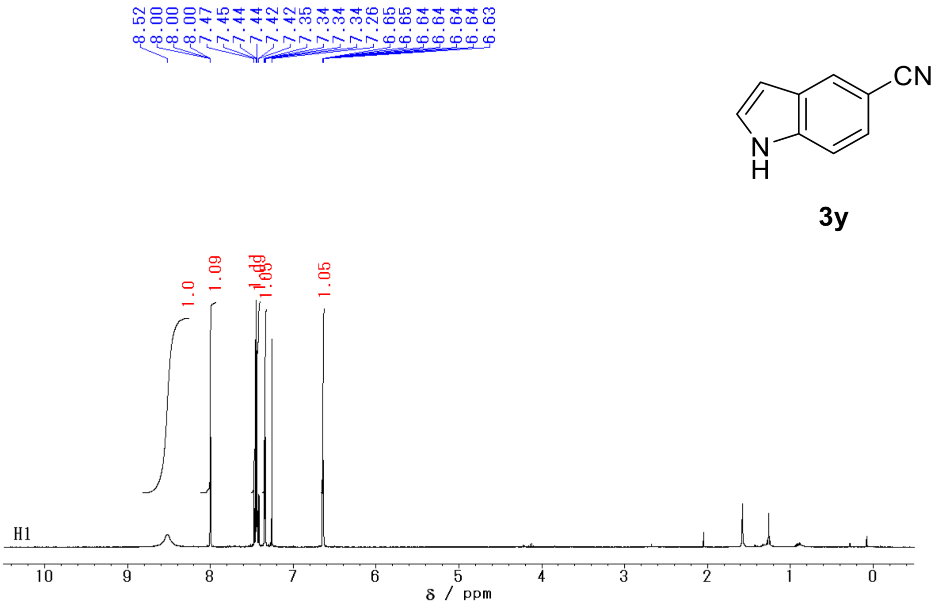

Figure S49. ^{1}H NMR spectrum of **3y**.

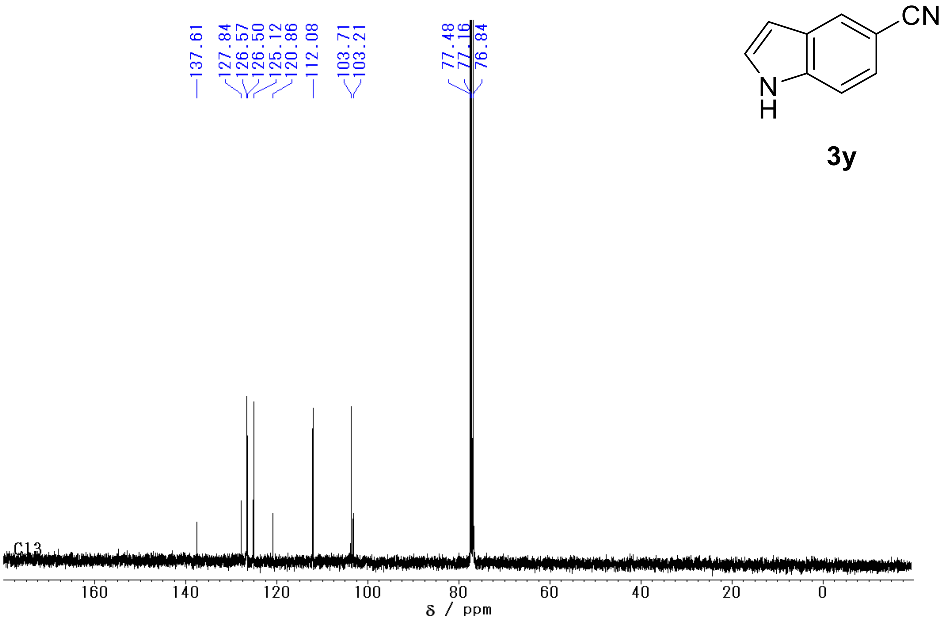

Figure S50. ^{13}C NMR spectrum of **3y**.

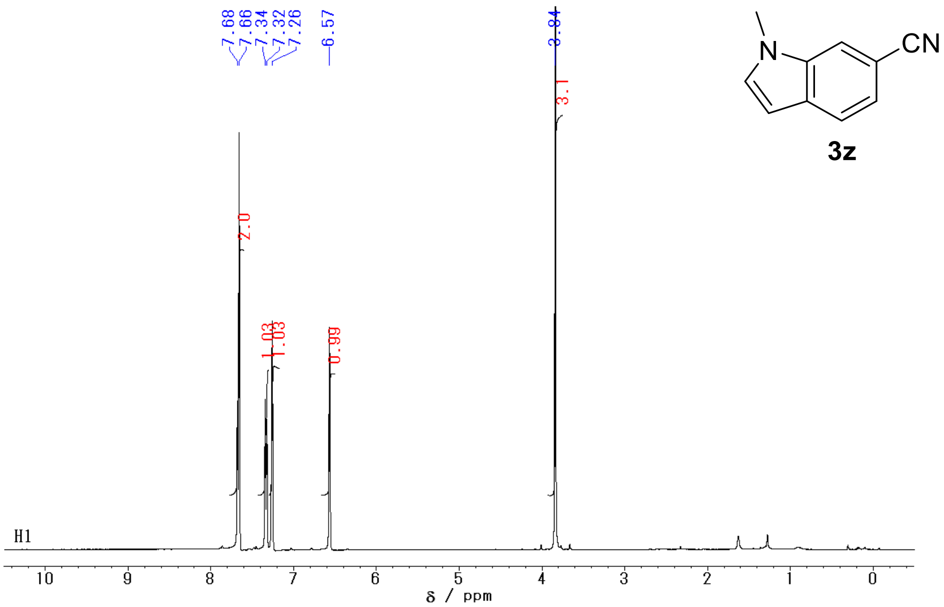

Figure S51. ^{1}H NMR spectrum of **3z**.

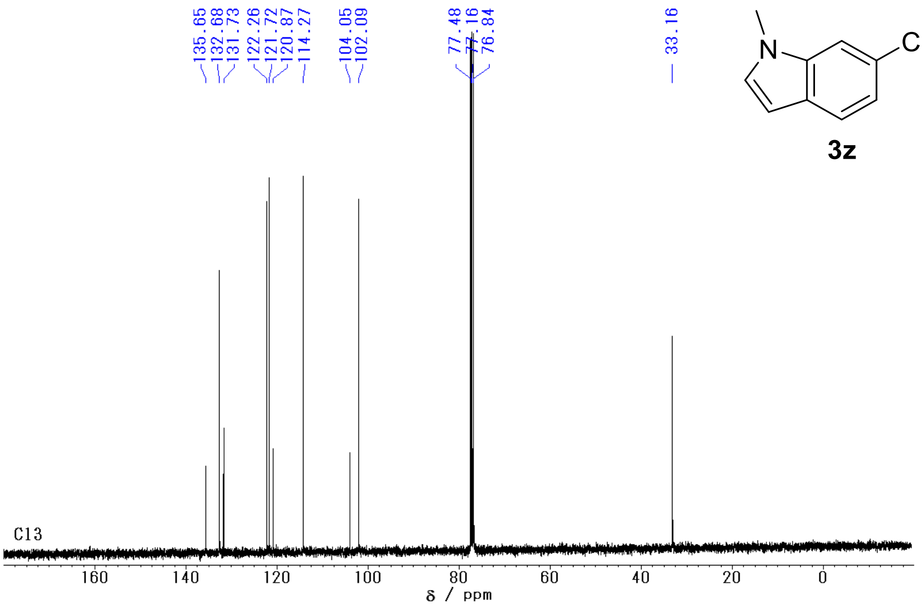

Figure S52. ^{13}C NMR spectrum of **3z**.

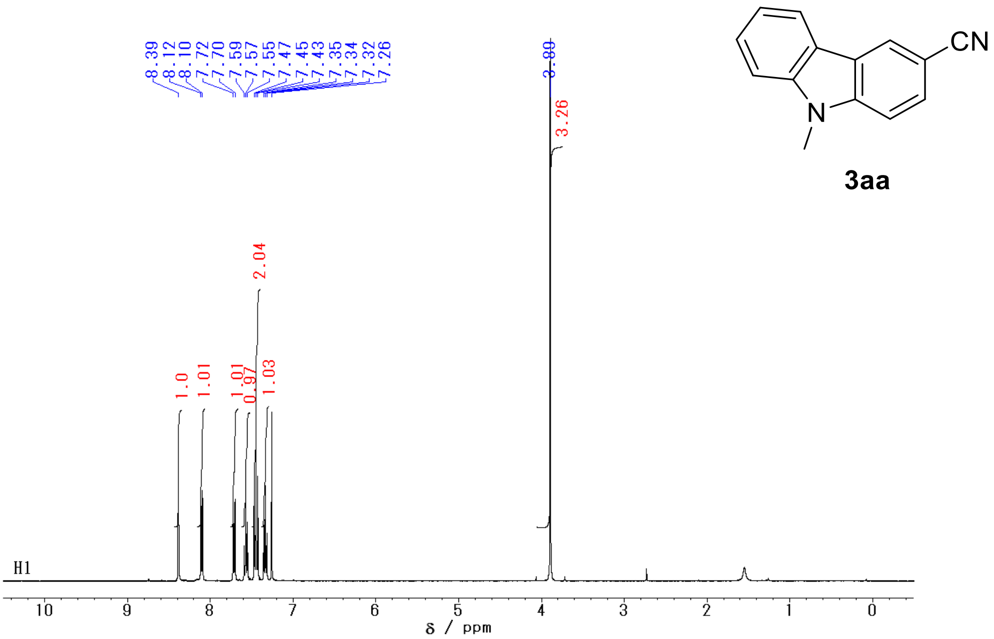

Figure S53. ^{1}H NMR spectrum of **3aa**.

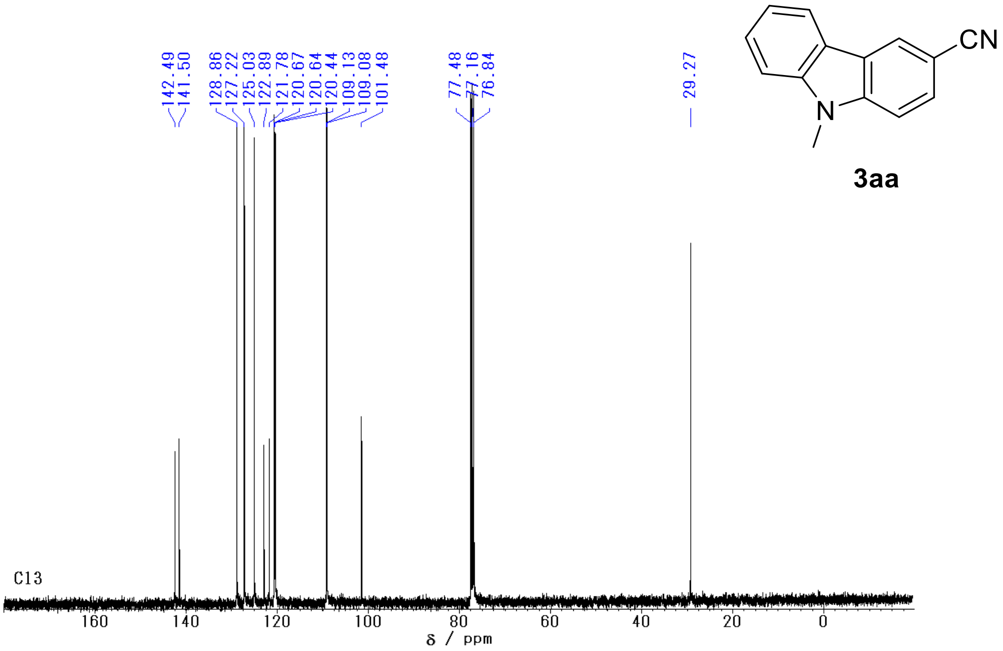

Figure S54. ^{13}C NMR spectrum of **3aa**.

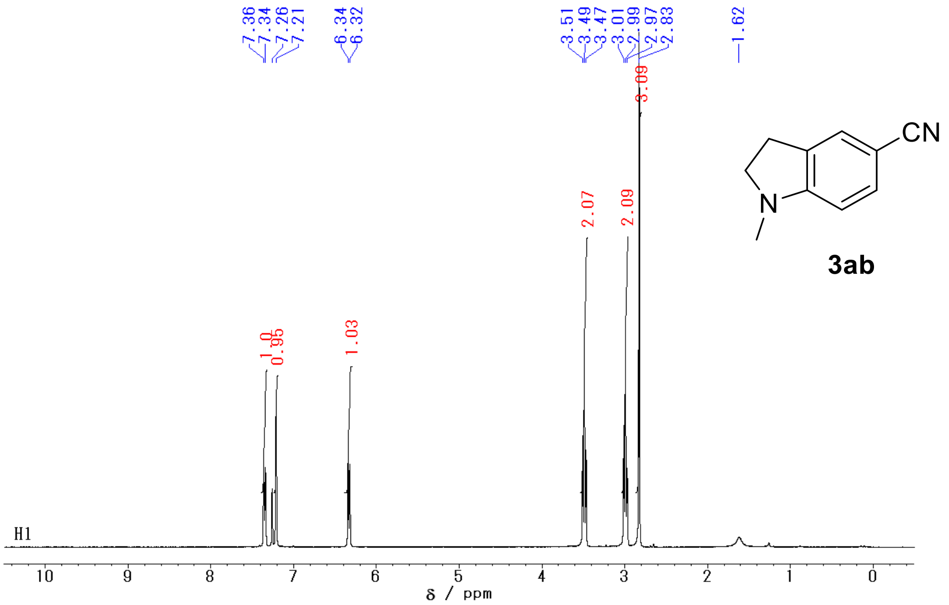

Figure S55. ^{1}H NMR spectrum of **3ab**.

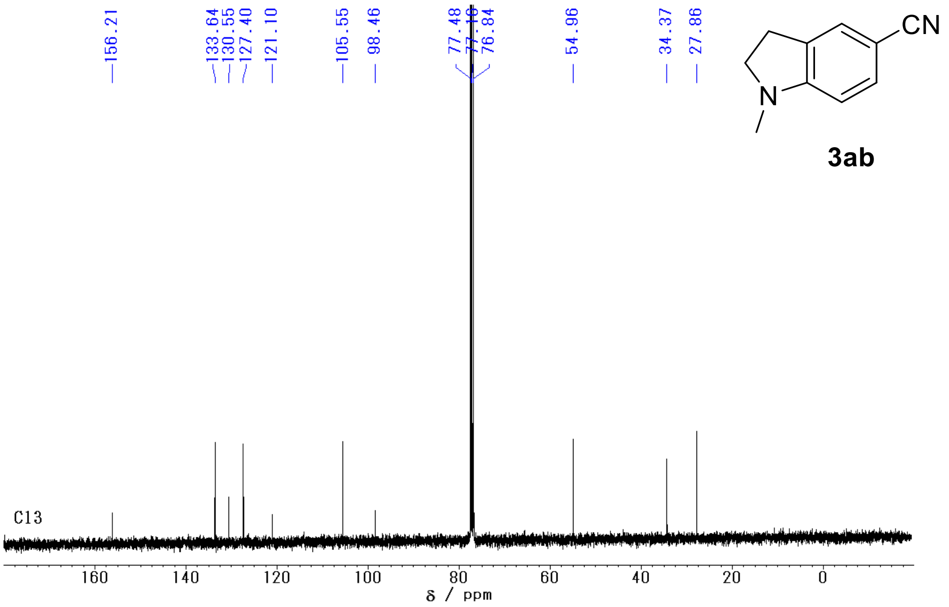

Figure S56. ^{13}C NMR spectrum of **3ab**.

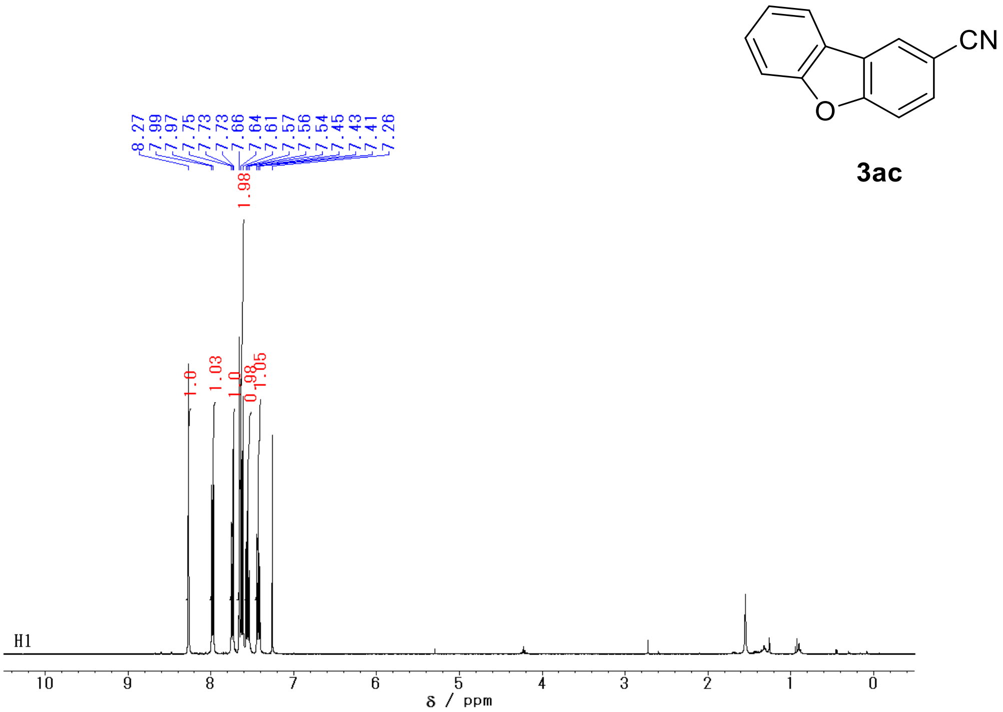

Figure S57. ^{1}H NMR spectrum of **3ac**.

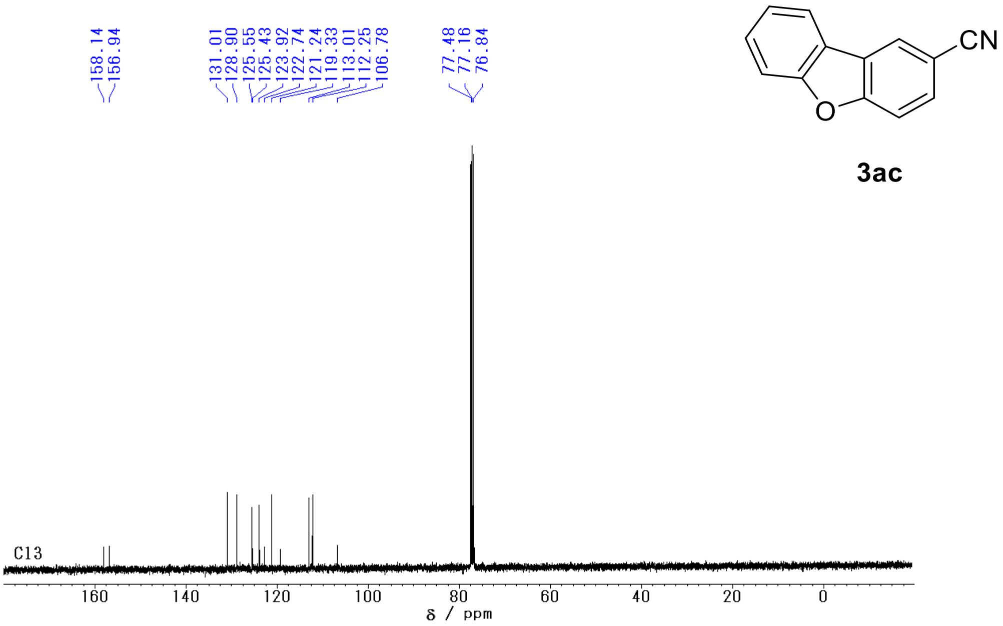

Figure S58. ^{1}H NMR spectrum of **3ac**.

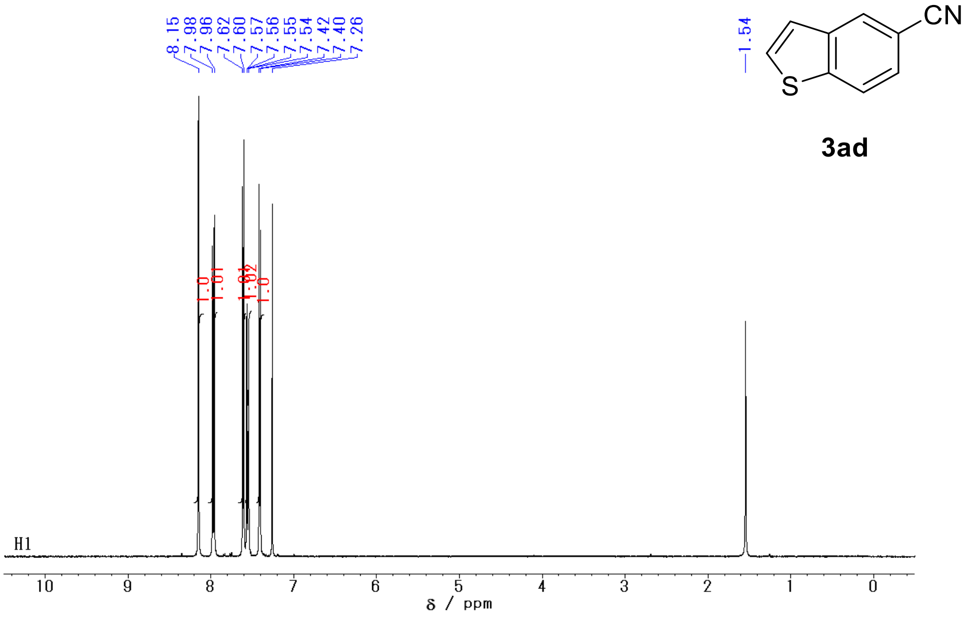

Figure S59. ^{1}H NMR spectrum of **3ad**.

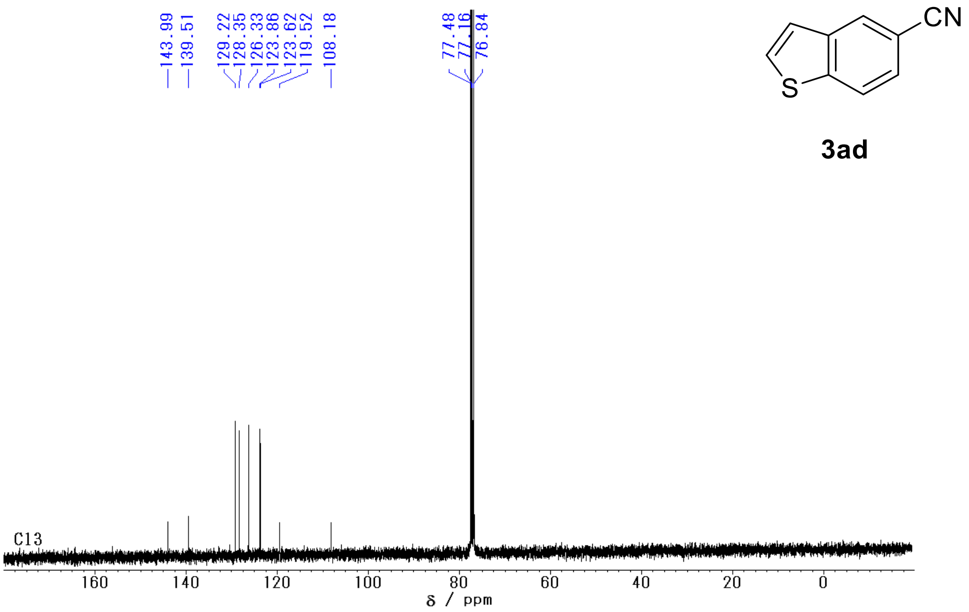

Figure S60. ^{13}C NMR spectrum of **3ad**.

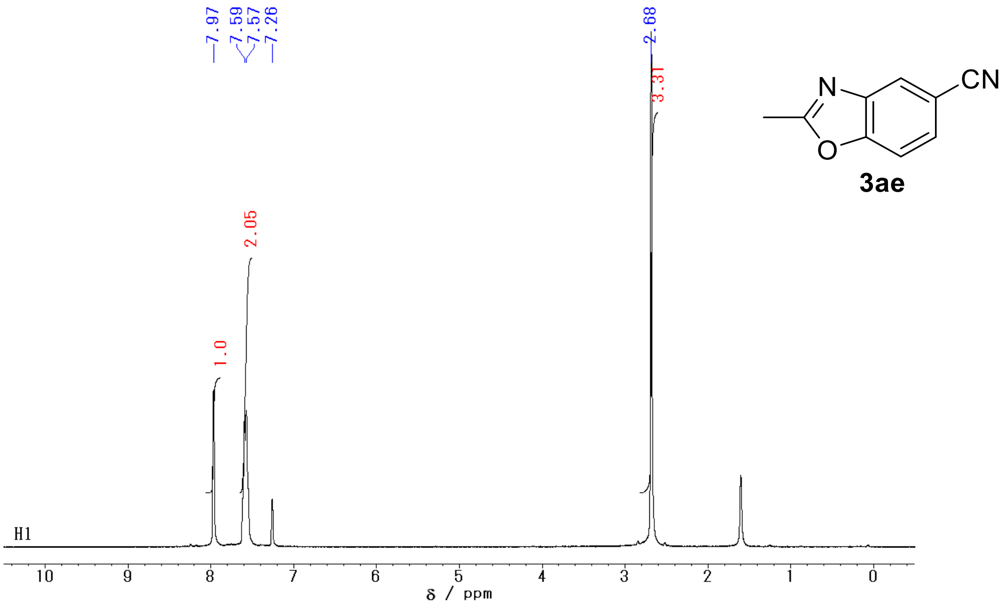

Figure S61. ^{1}H NMR spectrum of **3ae**.

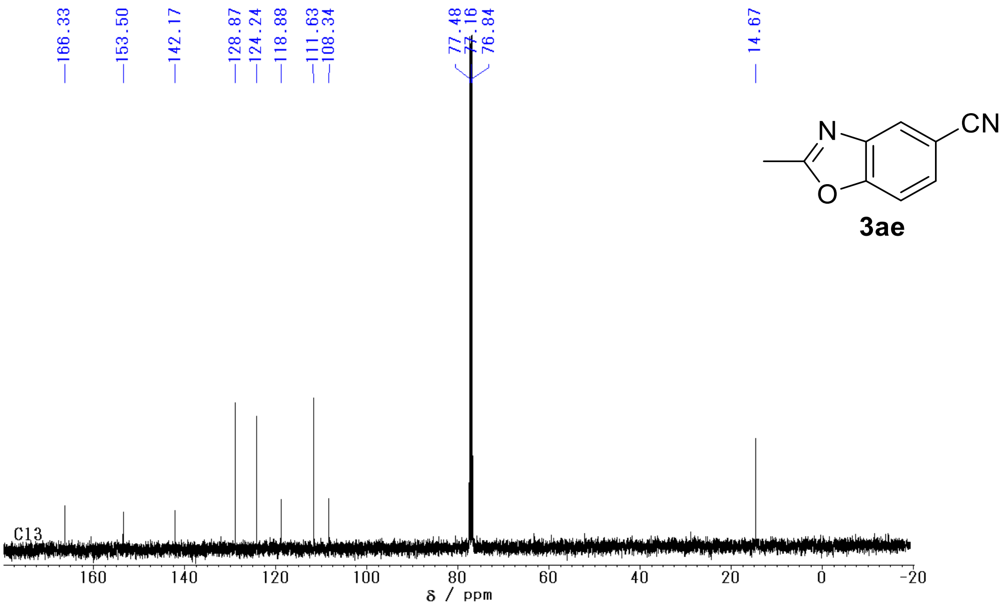

Figure S62. ^{13}C NMR spectrum of **3ae**.

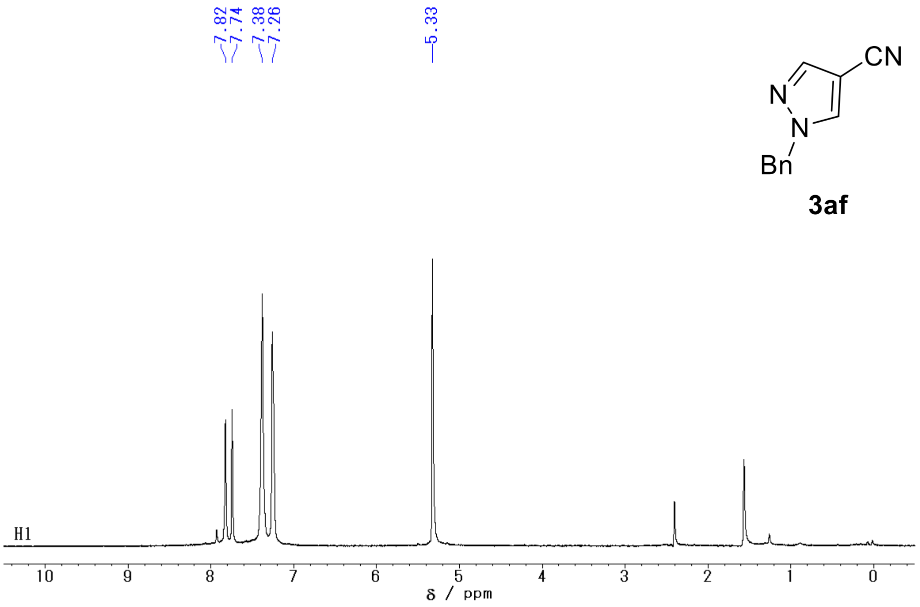

Figure S63. ^{1}H NMR spectrum of **3af**.

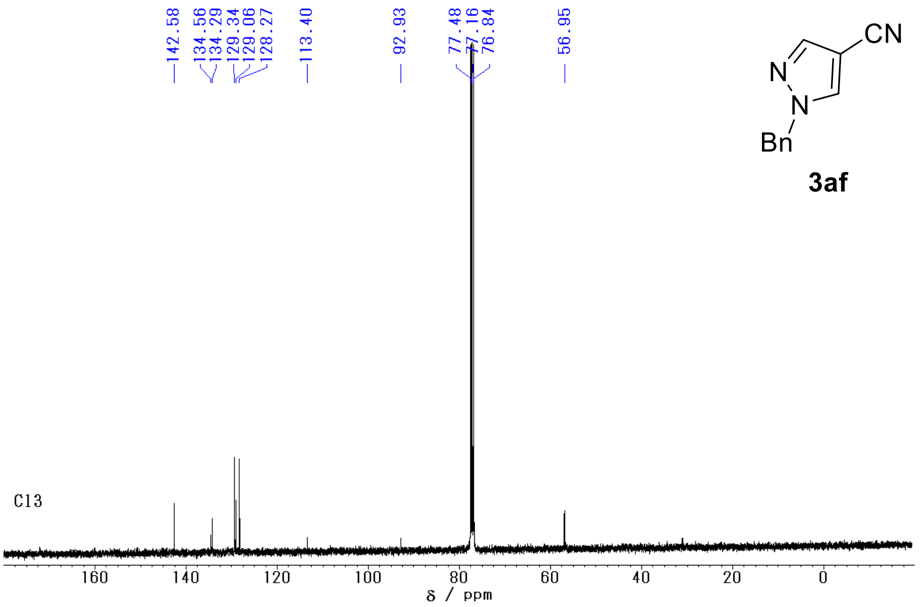

Figure S64. ^{13}C NMR spectrum of **3af**.

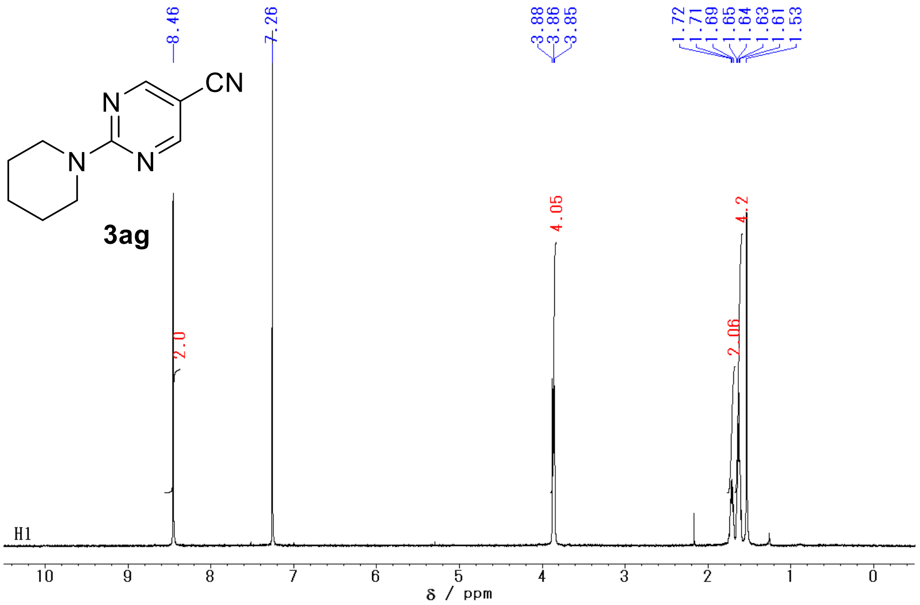

Figure S65. ^{1}H NMR spectrum of **3ag**.

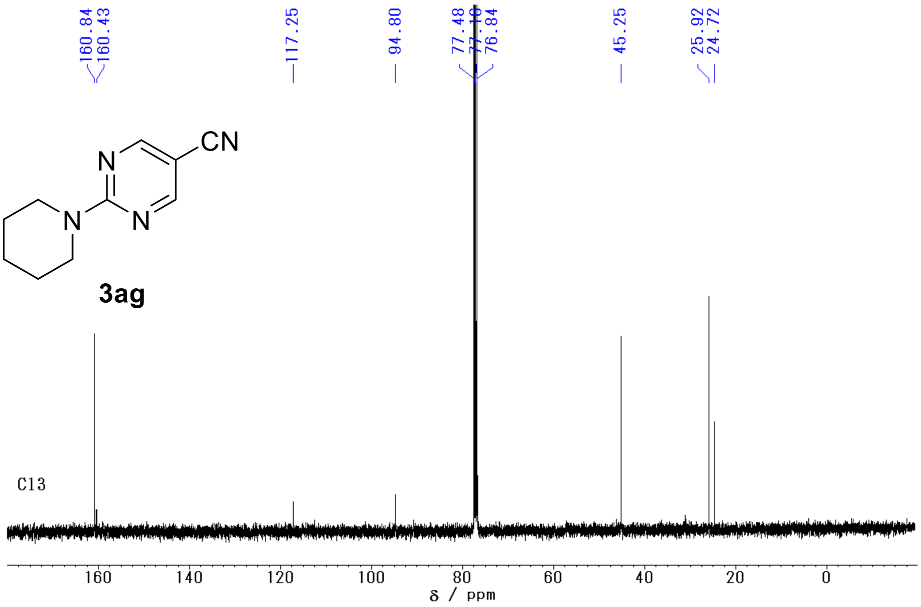

Figure S66. ^{13}C NMR spectrum of **3ag**.

11. References

1. T. Saito, H. Nishiyama, H. Tanahashi, K. Kawakita, H. Tsurugi and K. Mashima, *J. Am. Chem. Soc.,* 2014, **136**, 5161.
2. R. F. de Souza, D. Thiele and A. L. Monterio, *J. Catal.,* 2006, **241**, 232.
3. V. R. amírez-Delgado, G. Osorio-Monreal, L. F. Hernández-Ayala, Y. Reyes-Vidal, J. C. García-Ramos, L. Ruiz-Azuara, and L. Ortiz-Frade, *J. Mex. Chem. Soc.,* 2015, **59**, 294.
4. A. V. Müller, L. D. Ramos, K. P. M. Frin, K. T. de Oliveira and A. S. Polo, *RSC Adv.,* 2016, **6**, 46487.
5. H. V. Chavan, L. K. Adsul and B. P. Bandgar, *J. Chem. Sci.,* 2011, **123**, 477.
6. E. K. Edelstein, S. Namirembe and J. P. Morken, *J. Am. Chem. Soc.,* 2017, **139**, 5027.
7. (a) J. C. Tellis, D. N. Primer and G. A. Molander, *Science,* 2014, **345**, 433; (b) O. Gutierrez, J. C. Tellis, D. N. Primer, G. A. Molander and M. C. Kozlowski, *J. Am. Chem. Soc.,* 2015, **137**, 4896.
8. J. Kim, J. Choi, K. Shin and S. Chang, *J. Am. Chem. Soc.,* 2012, **134**, 2528.
9. S. Zheng, C. Yu and Z. Shen, *Org. Lett.,* 2012, **14**, 3644.
10. P. Yu and Morandi, *Angew. Chem., Int. Ed.,* 2017, **56**, 15693; *Angew. Chem.* 2017, **129**, 15899.
11. R. Möckel, J. Hille, E. Winterling, S. Weidemüller, T. M. Faber and G. Hilt, *Angew. Chem., Int. Ed.* 2018, **57**, 442.
12. T. Shen, T. Wang, C. Qin and N. Jiao, *Angew. Chem., Int. Ed.,* 2013, **52**, 6677.
13. S. D. Straight, J. Andréasson, G. Kodis, A. L. Moore, T. A. Moore and D. Gust, *J. Am. Chem. Soc.,* 2005, **127**, 2717.
14. Y. Zhu, M. Zhao, W. Lu, L. Li and Z. Shen, *Org. Lett.,* 2015, **17**, 2602.
15. T. Tamura, K. Moriyama and Togo, *Eur. J. Org. Chem.,* 2015, **9**, 2023.
16. X. Jiang, J.-M. Wang, Y. Zhang, Z. Chen, Y.-M. Zhu and S.-J. Ji, *Tetrahedron,* 2015, **71**, 4883.
17. B. V. Rokade, S. K. Malekar and K. R. Prabhu, *Chem. Commun.,* 2012, **48**, 5506.
18. R. Takise, K. Itami and J. Yamaguchi, *Org. Lett.,* 2016, **18**, 4428.
19. N. Sun, L. Hong, F. Huang, H. Ren, W. Mo, B. Hu, Z. Shen and X. Hu, *Tetrahedron,* 2013, **69**, 3927.
20. P. H.-Y. Cheong, R. S. Paton, S. M. Bronner, G.-Y. J. Im, N. K. Garg and K. N. Houk, *J. Am. Chem. Soc.,* **2010,** *132,* 1267.

21. S. Ushijima, K. Moriyama and H. Togo, *Tetrahedron,* 2012, **68**, 4588.
22. K. A. Zachariasse, S. I. Druzhinin, W. Bosch and R. Machinek, *J. Am. Chem. Soc.,* 2004, **126**, 1705.
23. J. Y. Cho, G.-B. Roh and E. J. Cho, *J. Org. Chem.,* 2018, **83**, 805.
24. Y.-L. Shi, Q. Yuan, Z.-B. Chen, F.-L. Zhang, K. Liu and Y.-M. Zhu, *Synlett,* 2018, **29**, 359.
25. X. Zhang, A. Xia, H. Chen and Y. Liu, *Org. Lett.,* 2017, **19**, 2118.
26. D. T. Cohen and S. L. Buchwald, *Org. Lett.,* 2015, **17**, 202.
27. G. M. Sheldrick, *Acta Cryst.,* 2014, **A70**, C1437.
28. G. M. Sheldrick, *Acta Cryst.,* 2008, **A64**, 112.
29. L. J. Farrugia, *J. Appl. Cryst.,* 1999, **32**, 837.
